# Enhancing the Antimicrobial Photodynamic Activity of Heteroleptic Ruthenium(II) Complexes: Role of Asymmetric *N*‑Donor-Based Ligands

**DOI:** 10.1021/acs.inorgchem.6c00420

**Published:** 2026-06-26

**Authors:** Abir Mesra-Brahmi, Sofia Martínez-Olmo, Isabel Guerrero, Xavier Fontrodona, Jordi Poater, Ariadna Verdaguer, Maria Auset Vallejo, Roger Bresolí-Obach, Santi Nonell, Rosario Núñez, Isabel Romero

**Affiliations:** † Departament de Química and Serveis Tècnics de Recerca, 16738 Universitat de Girona, C/M. Aurèlia Capmany, 69, Girona E-17003, Spain; ‡ Institut Químic de Sarrià, Universitat Ramon Llull, Barcelona 08017, Spain; § 54449 Institut de Ciència de Materials de Barcelona, ICMAB-CSIC, Carrer dels Til.lers Cerdanyola del Vallés 08193, Spain; ∥ Departament de Química Inorgànica i Orgànica & IQTCUB, Universitat de Barcelona, Martí i Franquès 1-11, Barcelona 08028, Spain; ⊥ Catalan Institution for Research & Advanced Studies, ICREA, Pg. Lluís Companys 23, Barcelona 08010, Spain

## Abstract

With their highly stability and tunable photophysical properties, Ru­(II) complexes are excellent photosensitizer candidates for antimicrobial photodynamic therapy (aPDT), offering selective bacterial targeting without driving resistance. A series of heteroleptic Ru­(II) N-donor complexes, incorporating symmetric N-bidentate ligands such as 1,10-phenanthroline and 4,4′-diphenyl-2,2′-bipyridine, together with asymmetric N-bidentate ligands including pyridine–pyrazole, iminopyridines, and iminoquinoline, have been successfully synthesized to assess their potential as photosensitizers for aPDT. All Ru­(II) complexes exhibited distorted octahedral coordination, unique ligand arrangements, and hydrophobicity, which influenced their electronic properties and photodynamic activity. TDDFT calculations reliably reproduced experimental trends. All complexes produce singlet oxygen and superoxide radical anion upon exposure to visible light and are capable of photoinactivating *S. aureus* bacteria at micromolar concentrations. Complex **2**, containing two phenanthrolines and a pyridine–pyrazole ligand, displays the highest potential for aPDT due to its optimized ligand structure, which enhances its physicochemical and optical properties and singlet oxygen production. It penetrates the bacterial cytoplasm and damages genomic DNA, efficiently neutralizing both Gram-positive and Gram-negative strains, as well as *Candida albicans* yeasts to a lesser extent. Meanwhile, it shows no toxicity toward human fibroblasts within therapeutic dose ranges. By bridging the gap between molecular structure and biological function, these results establish novel design criteria for choosing ligands to develop effective, selective Ru­(II) aPDT agents.

## Graphical abstract

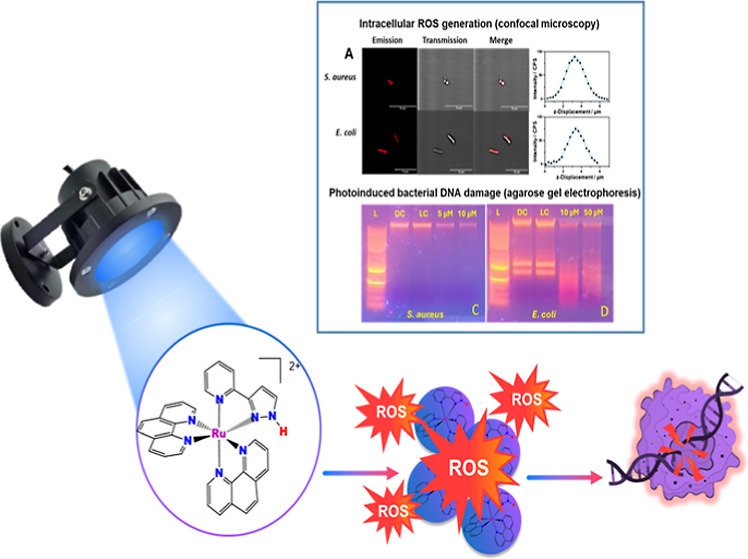

## Introduction

According to the World Health Organization (WHO), antimicrobial resistance (AMR) represents a critical threat to global development and public health.^1^ In 2019, AMR was linked to approximately 4.95 million deaths worldwide, with 1.27 million of these deaths directly caused by multidrug-resistant bacteria. The overuse and misuse of antibiotics exacerbate this problem. Looking ahead, the situation is expected to worsen, with an estimated 8.22 million deaths annually by 2050 if the current conditions persist.^2^ Although the search for new antibiotics might offer a short-term solution, it will ultimately fail in the long term because bacteria develop resistance mechanisms faster than new antibiotics can be developed. Therefore, it is imperative to develop alternative treatments to antibiotics.^3^ Antimicrobial photodynamic therapy (aPDT) could be a viable solution.^4^ In aPDT, three elements are used: a photosensitive drug or photosensitizer (PS), light, and oxygen, which are harmless on their own. However, when combined, they generate highly destructive reactive oxygen species (ROS) such as singlet oxygen (^1^O_2_) that can readily oxidize cellular components and thereby cause the death of bacterial populations. Moreover, aPDT is effective against a wide range of bacterial infections, prevents the emergence of resistance and restores antibiotic susceptibility in resistant strains after treatment due to its multitarget and nonspecific mode of action.^5^

For a PS to be effective in aPDT, it must exhibit certain photophysical characteristics, including a high absorption coefficient in the visible and a high quantum yield for producing cytotoxic ROS.^5^ Various chromophores have been employed as PSs to deactivate microbial cells, with many derived from porphyrinoid, phenothiazinium, or xanthene scaffolds. Furthermore, PSs should exhibit high water solubility, a pronounced affinity for microbial targets, and minimal binding to host cells. These essential characteristics are intimately associated with the incorporation of cationic charges within the molecular framework.^6^ The realization that PSs with positive charges at physiological pH values enhance the photoinactivation of microbial cells has driven the development of new synthetic methods to create effective cationic PSs for aPDT.^4,5^ Among the various PSs currently under investigation, Ru­(II) complexes have demonstrated considerable potential, owing to their desirable photophysical characteristics. Thus, Ru­(II) complexes have been widely studied and applied in diverse fields as photocatalysis,^7,8^ solar energy conversion,^9^ luminescent bioimaging,^10^ and photodynamic therapy (PDT).^11–14^ In the context of aPDT, these compounds offer several key advantages, (i) tunable photophysical properties through ligand modification;^15^ (ii) high photostability, low dark toxicity and quantum yields of ROS generation, especially ^1^O_2_; (iii) selectivity targeting and accumulation in bacterial cells and (iv) reduced potential for resistance development, since ROS act on multiple cellular components simultaneously, unlike conventional antibiotics that typically target a single site.^16–22^

Many Ru­(II) polypyridyl complexes designed as red-light-activated phototherapeutic agents rely on low-lying ^3^MLCT states to mediate energy or electron transfer and, in some cases, photoactivated ligand release, which ultimately governs their PDT activity.^23,24^ In contrast, π-extended ligands can generate lowest-lying intraligand (^3^IL) or IL charge-transfer (^3^ILCT) states with microsecond lifetimes, which greatly enhance ^1^O_2_ quantum yields and photocytotoxicity in vitro and in vivo, as recently shown for NIR-absorbing Ru­(II) complexes.^25,26^ Despite their promising potential, further research is required to optimize their selectivity, reduce toxicity to healthy tissues, and improve their efficacy under physiological conditions (e.g., variable pH, hypoxia).^27,28^ In addition, a deeper understanding of structure-activity relationships, along with improvements in water solubility and cellular uptake, is essential to advance these compounds toward clinical application.^29,30^

In this study, we synthesized and characterized a series of heteroleptic Ru­(II) complexes, incorporating diverse *N*-donor ligands (Scheme 1): 1,10-phenantroline (L1), 2-(1*H*-pyrazol-3-yl)­pyridine (L2), two iminopyridine-based ligands, namely (*E*)-*N*-phenyl-1-(pyridin-2-yl)­methanimine (L3) and (E)-*N*,*N*-dimethyl-4-((pyridin-2-ylmethylene)­amino)­aniline (L4), the iminoquinoline (*E*)-*N*,*N*-dimethyl-4-((quinolin-2-ylmethylene)­amino)­aniline (L5), and 4,4′-diphenyl-2,2′-bipyridine (L6). These ligands were selected for their distinct physicochemical and electronic properties, which are critical for tailoring the photochemistry and biological activity of coordination complexes. Of specific interest in this work, the ligands endow the complexes with different degrees of hydrophilicity, with complex **2** being the most hydrophilic and complex **6** being the most hydrophobic. Building on this structural diversity, we present a comprehensive study of the optical, photophysical, and photosensitizing properties of these heteroleptic Ru­(II) complexes and evaluate their potential as photoantimicrobial agents against representative bacterial and fungal strains. The experimental findings were supported by theoretical calculations performed using time-dependent density functional theory (TDDFT), providing deeper insight into their photophysical behavior. This integrated experimental-theoretical framework provides insights into the rational design of Ru­(II)-based PSs for applications in aPDT and antimicrobial materials.

**Scheme 1:**
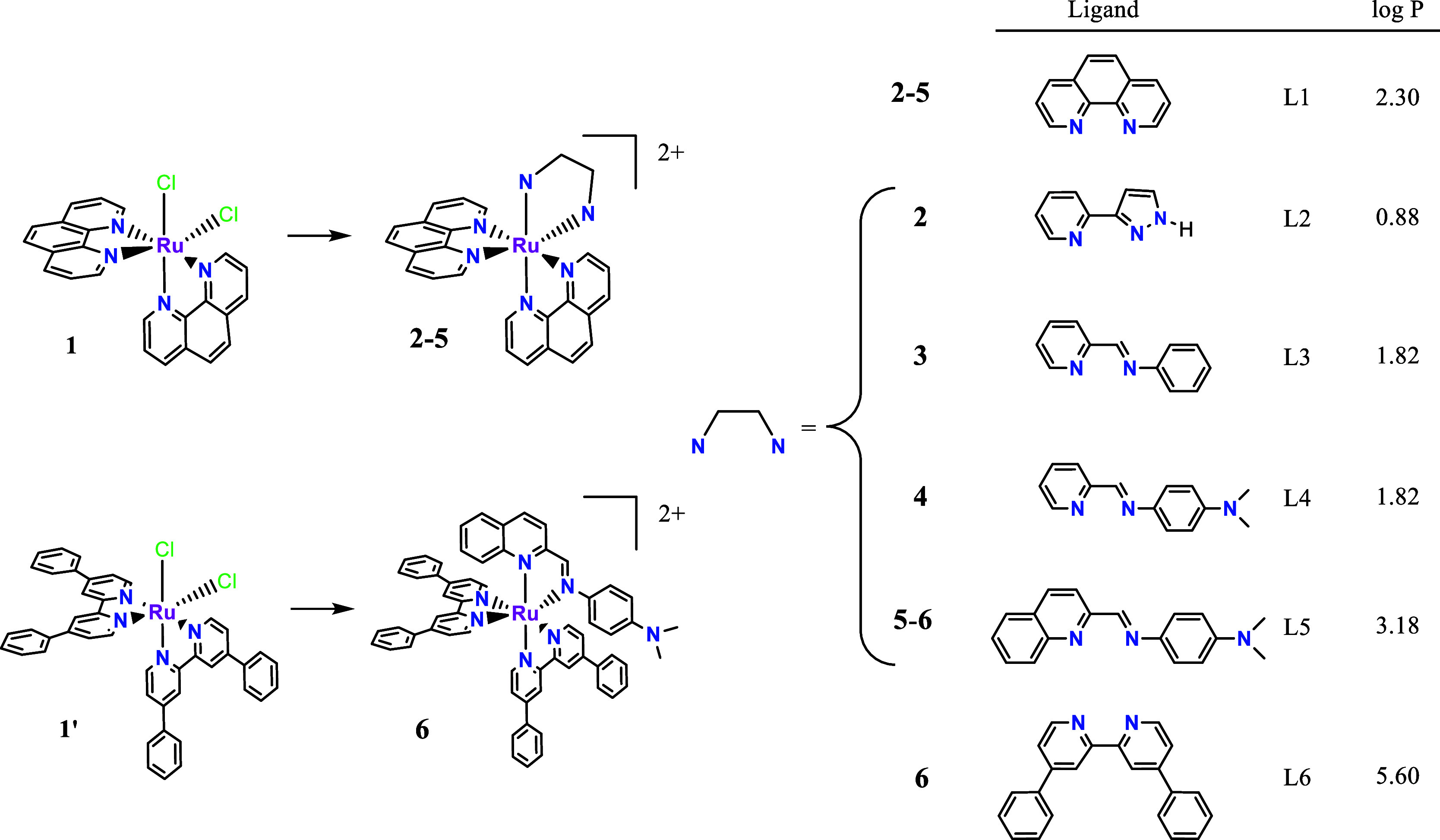
Molecular Structures of the Ru­(II) Complexes (**1-6**) and Their Corresponding Ligands (L1-L6) Studied in This Work. Calculated *n*-Octanol/Water Partition Coefficients (logP) for the Ligands, Were Determined Using the ACD/Labs Percepta Platform-PhysChem Module Version: 14 (chemspider.com)

## Experimental Section

### Materials

All chemical reagents were purchased from Sigma-Aldrich and used as received. Reagent-grade organic solvents were supplied by Carlo Erba, and high-purity deionized water was prepared by passing distilled water through a Milli-Q nanopure purification system. Ligand L*2* ^31^ and complexes **1** and **1’** ^14^ were prepared according to literature procedures. Optical properties were evaluated in anhydrous grade dichloromethane, acetonitrile and methanol purchased from Sigma-Aldrich and used without further purification.

### Synthesis and Characterization of the Complexes

#### Synthesis of Complex **2**

A mixture of **1** (0.120 g, 0.226 mmol), ligand L2 (0.033 g, 0.226 mmol), and NH_4_OAc (0.070 g, 0.914 mmol), was refluxed in 25 mL of MeOH/H_2_O (20:5) for 5 h. The reaction was conducted under a nitrogen atmosphere in the absence of light. Upon cooling to ambient temperature, a saturated aqueous solution of NH_4_PF_6_ was added, resulting in the formation of an orange precipitate. The solid was collected via filtration, washed with ice-cold water and diethyl ether, and dried under vacuum. Yield: 68% (0.138 g). Anal. Found (Calc.) for C_32_H_23_N_7_RuP_2_F_12_·CH_3_OH: 42.90(42.68); H, 2.86 (2.93); N, 10.31 (10.56). IR (ν, cm^–1^): 3300, 3100, 1600, 1510, 1427, 844. ^1^H NMR (400 MHz, CD_2_Cl_2_) δ 11.51 (s, 1H, H_7_), 8.60 (dd, *J*_c,c’_ = 8.3, 1.3 Hz, 2H, H_c,c’_), 8.51 (dd, *J*_c’’_ = 8.3, 1.3 Hz, 1H, H_c’’_), 8.45 (dd, *J*_c’’’_ = 8.3, 1.3 Hz, 1H, H_c’’’_), 8.25 (dt, *J*_a,a’_ = 5.3, 1.4 Hz, 2H, H_a,a’_), 8.22–8.14 (m, 4H, H_d,d’,d’’,d’’’_), 8.11 (dt, *J*_4_ = 8.0, 1.2 Hz, 1H, H_4_), 7.97 (td, *J*_3_ = 7.8, 1.5 Hz, 1H, H_3_), 7.94–7.84 (m, 4H, H_a’’,a’’’,b,b’_), 7.82 (d, *J*_6_ = 2.9 Hz, 1H, H_6_), 7.68 (dd, *J*_b’’_ = 8.3, 5.3 Hz, 1H, H_b’’_), 7.64–7.54 (m, 2H, H_b’’’,1_), 7.25 (ddd, *J*_2_ = 7.4, 5.7, 1.5 Hz, 1H, H_2_), 7.17 (d, *J*_5_ = 2.9 Hz, 1H, H_5_). ^13^C NMR (400 MHz, CD_2_Cl_2_) δ 136.70 (C_c’’_), 136.69 (C_c’’’_), 136.52 (C_c,c’_), 128.24 (C_a,a’_), 128 (C_d,d’,d’’,d’’’_), 126.23 (C_a’’,a’’’,b,b’_), 126.21 (C_b’’_), 126.08 (C_6_), 126.01 (C_b’’’,1_), 125.81 (C_2_), 125.25 (C_3_), 122.65 (C_4_), 105.15 (C_5_). IR (ν, cm^–1^): 3328, 3082, 1610, 1509, 1423, 820, 719. UV/vis (CH_2_Cl_2_), [λ (nm) (ε, cm^–1^ M^–1^)]: 265 (103950), 405 (15760), 454 (13230). E_1/2_(Ru^III^/Ru^II^)­(CH_3_CN + 0.1 M TBAH): 1.18 V vs SCE. ESI-MS: 606.1 [M – H^+^-2PF_6_]^+^; 303.5 [M--2PF_6_]^2+^.

#### Synthesis of Complex **3**

A mixture of aniline (55 μL, 0.6 mmol) and 2 pyridinecarboxaldehyde (57 μL, 0.6 mmol) was refluxed in 20 mL of dry MeOH for 12h under N_2_ atmosphere for in situ preparation of ligand L3. Complex **1** (0.2662 g, 0.5 mmol) was added to the resulted solution, and the new mixture was refluxed for an additional 12 h in the dark. Subsequently, the solution was allowed to cool to room temperature. The addition of a saturated aqueous solution of NH_4_PF_6_ induced the formation of an orange precipitate, which was isolated via filtration, washed with diethyl ether, and dried under vacuum.

Yield: 86.5% (0.404 g). Anal. Found (Calc.) for C_36_H_26_N_6_P_2_F_12_Ru·CH_3_OH·H_2_O: C, 45.22 (45.18); H, 3.04 (3.28); N, 8.63 (8.54). IR (ν, cm^–1^): 3078, 1580, 1427, 1204, 824. ^1^H NMR­(400 MHz, CD_2_Cl_2_) δ 9.12 (d, *J*_c_ = 4.9 Hz, 1H, H_c_), 9.09 (s, 1H, H_5_), 8.73 (d, *J*_c’_ = 8.1 Hz, 1H, H_c’_), 8.67 (d, *J*_c’’_ = 8.1 Hz, 1H, H_c’’_), 8.57 (d, *J*_c’’’_ = 8.1 Hz, 1H, H_c’’’_), 8.41 (d, *J*_4_ = 7.8 Hz, 1H, H_4_), 8.33–8.19 (m, 4H, H_d,d’,d’’,d’’’_), 8.17–8.02 (m, 5H, H_b,b’,b’’,a,3_), 7.93 (d, *J*_a’_ = 8.9 Hz, 1H, H_a’_), 7.72 (m, 3H, H_a’’,b’’’,1_), 7.45 (m, 2H, H_a’’’,2_), 6.95 (t, *J*_8_ = 7.4 Hz, 1H, H_8_), 6.83 (t, *J*_7_ = 7.6 Hz, 2H, H_7_), 6.37 (d, *J*_6_ = 7.7 Hz, 2H, H_6_). ^13^C NMR (400 MHz, CD_2_Cl_2_) δ 153.91 (C_5_), 153.69 (C_c_), 137.47 (C_c’’_), 137.41 (C_c’_), 137.18 (C_c’’’_), 130.35 (C_4_), 128.97 (C_7_), 128.28 (C_8_), 128.17, 128.4, 136.23, 153.07 (C_d,d’,d’’,d’’’_), 127.59 (C_a’_), 127.37, 127.71, 137.52, 152.72 (C_b,b’,a,3_), 125.99, 126.33, 151.81 (C_a’’,b’’’,1_), 125.76, 128.97 (C_a’’’,2_), 120.34 (C_6_). UV–vis (CH_2_Cl_2_), [λ (nm) (ε, cm^–1^ M^–1^)]: 265 (99240), 429 (14680), 479 (13210). E_1/2_(Ru^III^/Ru^II^)­(CH_3_CN + 0.1 M TBAH): 1.36 V vs SCE. ESI-MS: 789,1 [M-PF_6_]^+^; 322,1 [M-2PF_6_]^2+^.

#### Synthesis of Complex **4**

A mixture of 2-pyridinecarboxaldehyde (56.8 μL, 0.6 mmol) and *N*,*N*-dimethyl-4,4-phenylaniline (0.082 g, 0.6 mmol) was refluxed in 20 mL of dry MeOH for 12h under N_2_ atmosphere for in situ preparation of ligand L4. Complex **1** (0.266 g, 0.5 mmol) was added to the resulting solution, and the new mixture was refluxed for additional 12 h in the dark. Subsequently, the solution was allowed to cool to room temperature. The addition of a saturated aqueous solution of NH_4_PF_6_ induced the formation of an orange precipitate. The solid was purified using an alumina column (CH_2_Cl_2_: MeOH, 98:2). Yield: 44.6% (0.217 g). Anal. Found (Calc.) for C_38_H_31_N_7_P_2_F_12_Ru·1/2CH_3_OH·H_2_O: C, 45.99 (45.70); H, 3.10 (3.46); N, 9.34 (9.60). IR (ν, cm^–1^): 3078, 1580, 1427, 1204, 824. ^1^H NMR­(400 MHz, CD_2_Cl_2_) δ 9.03 (dd, *J*_*c*_ = 5.2, 1.2 Hz, 1H, H_c_), 8.96 (s, 1H, H_5_), 8.71 (dd, *J*_a_ = 8.3, 1.3 Hz, 1H, H_a_), 8.66 (dd, *J*_c’_ = 8.3, 1.2 Hz, 1H, H_c’_), 8.55 (dd, *J*_*c’’*_ = 8.2, 1.3 Hz, 1H, H_c’’_), 8.34–8.30 (m, 2H, H_c’’’,a’_), 8.30–8.22 (m, 2H, H_d,d’_), 8.17 (dd, J_4_ = 5.2, 1.2 Hz, 1H, H_4_), 8.15–7.97 (m, 6H, H_d’’,d’’’,b‑b’,a’,3_), 7.73 (dd, *J*_*a’’’*_ = 5.2, 1.3 Hz, 1H, H_a’’’_), 7.69 (dd, *J*_*b’’*_ = 8.2, 5.3 Hz, 1H, H_b’’_), 7.63 (d, J_1_ = 5.4 Hz, 1H, H_1_), 7.51 (dd, *J*_*b’’’*_ = 8.2, 5.3 Hz, 1H, H_b’’’_), 7.37 (ddd, J_2_ = 7.4, 5.6, 1.4 Hz, 1H, H_2_), 6.39 (d, J_6_ = 9.0 Hz, 2H, H_6_), 6.10 (d, J_7_ = 9.1 Hz, 2H, H_7_), 2.79 (s, 6H, H_8_). ^13^C NMR (400 MHz, CD_2_Cl_2_) δ 153.98 (C_c_), 153.68 (C_5_), 152.93 (C_4_), 151.85 (C_a’’’_), 151.40 (C_1_), 137.32 (C_c’’_), 137.14 (C_c’_), 136.99 (C_a_), 129.82, 136.39 (C_d,d’_), 128.40, 136.54 (C_c’’’,a’_), 128.29 (C_2_), 127.09, 127.84, 137.32, 152.45 (C_d’’,d’’’,b,b’,a’’,3_) 126.34, 152.30 (C_b’’_), 125.75 (C_b’’’_), 121.86 (C_6_), 111.63 (C_7_), 40.24 (C_8_). UV–vis (CH_2_Cl_2_), [λ (nm) (ε, cm^–1^ M^–1^)]: 265 (111280), 439 (22860), 475 (25150). E_pa_(Ru^III^/Ru^II^)­(CH_3_CN + 0.1 M TBAH): 1.35 V vs SCE. ESI-MS: 832.2 [M-PF_6_]^+^; 343.6 [M-2PF_6_]^2+^.

#### Synthesis of Complex **5**

A mixture of 2-quinolinecarboxaldehyde (0.095 g, 0.6 mmol) and *N*,*N*-dimethyl-4,4-phenylaniline (0.082 g, 0.6 mmol) was refluxed in 10 mL of dry MeOH for 12h under N_2_ atmosphere for in situ preparation of ligand L5. Complex **1** (0.266 g, 0.5 mmol) was added to the resulting solution, and the new mixture was refluxed for additional 12 h in the dark. The solution was allowed to cool to room temperature. The addition of a saturated aqueous solution of NH_4_PF_6_ induced the formation of a Brown precipitate. The solid was filtered, washed with diethyl ether and vacuum-dried. Yield: 81.4% (0.417 g). Anal. Found (Calc.) for C_42_H_33_N_7_P_2_F_12_Ru: C, 48.91 (49.13); H, 3.33 (3.24); N, 9.38 (9.55). IR (ν, cm^–1^): 3078, 1606, 1517, 1427, 1204, 827. ^1^H NMR (400 MHz, CD_3_CN) δ 9.40 (s, 1H, H_7_), 9.02 (dd, *J*_c_ = 5.3, 1.3 Hz, 1H, H_c_), 8.72 (dd, *J*_*c’*_ = 8.3, 1.3 Hz, 1H, H_c’_), 8.64 (dd, *J*_a_ = 8.3, 1.2 Hz, 1H, H_a_), 8.59 (dd, *J*_*c’’*_ = 8.2, 1.3 Hz, 1H, H_c’’_), 8.55 (d, J_5_ = 8.4 Hz, 1H, H_5_), 8.40 (d, J_6_ = 8.4 Hz, 1H, H_6_), 8.28 (dd, *J*_*c’’’*_ = 5.3, 1.3 Hz, 1H, H_c’’’_), 8.26 (dd, *J*_*a’*_ = 8.0, 1.6 Hz, 1H, H_a’_), 8.23–8.16 (m, 3H, H_d‑d’‑d’’_), 8.14 (dd, *J*_*a’’*_ = 5.3, 1.3 Hz, 1H, H_a’’_), 8.08 (dd, *J*_*b*_ = 8.3, 5.2 Hz, 1H, H_b_), 8.02–7.94 (m, 2H, H_d’’’,4_), 7.90 (dd, *J*_*b’*_ = 8.3, 5.2 Hz, 1H, H_b’_), 7.75 (dd, *J*_*b’’*_ = 8.3, 5.3 Hz, 1H, H_b’’_), 7.59 (ddd, J_3_ = 8.1, 6.7, 1.1 Hz, 1H, H_3_), 7.43–7.32 (m, 2H, H_b’’’,a’’’_), 7.23 (d, *J*_1_ = 8.9 Hz, 1H, H_1_), 7.15 (ddd, *J*_2_ = 8.8, 6.7, 1.5 Hz, 1H, H_2_), 6.23 (d, J_8_ = 9 Hz, 2H, H_8_), 6.03 (d, J_9_ = 9 Hz, 2H, H_9_), 2.77 (s, 6H, H_10_). ^13^C NMR (400 MHz, CD_2_Cl_2_) δ 159.72 (C_7_), 154.03 (C_c_), 139.13 (C_c’’_), 138.99 (C_5_), 137.47 (C_c’_), 137.02 (C_a_), 132.43 (C_1_), 129.43 (C_3_), 129.87, 130.17 (C_d’’’,4_), 128.24, 128.09, 136.43 (C_d,d’,d’’_), 128.23, 136.43, 153.55 (C_a’_), 128.09, 153.55 (C_c’’’_), 127.94, 151.92 (C_a’’_), 126.76 (C_b’_), 126.31 (C_b’’_), 126.61 (C_b_), 125.87, 151.62 (C_b’’’,a’’’_), 124.54 (C_6_), 123.65, 132.13 (C_2_), 121.24 (C_8_), 110.27 (C_9_), 40.78 (C_10_). UV–vis (CH_2_Cl_2_), [λ (nm) (ε, cm^–1^ M^–1^)]: 265 (96930), 435 (12280), 505 (19140). E_pa_(Ru^III^/Ru^II^)­(CH_3_CN + 0.1 M TBAH): 1.51 V vs SCE. ESI-MS: 881.7 [M-PF_6_]^+^; 368.4 [M-2PF_6_]^2+^.

#### Synthesis of Complex **6**

A mixture of 2-quinolinecarboxaldehyde (0.014 g, 0.090 mmol) and *N*,*N*-dimethyl-4,4-phenylaniline (0.013 g, 0.093 mmol) was refluxed in 10 mL of dry MeOH for 12h under N_2_ atmosphere for in situ preparation of ligand L5. Complex **1’** (0.067 g, 0.084 mmol) was added to the resulting solution, and the new mixture was refluxed for 12 h in the dark. Subsequently, the solution was allowed to cool to room temperature. The addition of a saturated aqueous solution of NH_4_PF_6_ induced the formation of a brown precipitate. The solid was filtered, washed with diethyl ether and vacuum-dried. Yield: 51% (0.055 g). Anal. Found (Calc.) for C_62_H_49_N_7_P_2_F_12_Ru·CH_3_OH·H_2_O: C, 56.96 (56.76); H, 3.94 (4.16); N, 7.71 (7.35). IR (ν, cm^–1^): 3052, 1606, 1517, 1468, 1166, 827. ^1^H- NMR (400 MHz, CD_2_Cl_2_) δ 9.40 (s, 1H, H_7_), 9.04 (s, 1H, H_a_), 8.96 (s, 1H, H_a’_), 8.71 (s, 1H, H_a’’_), 8.67 (d, *J*_5_ = 8.4 Hz, 1H, H_5_), 8.52 (d, *J*_c_ = 6.0 Hz, 1H, H_c_), 8.41 (s, 1H, H_a’’’_), 8.36 (d, *J*_6_ = 8.4 Hz, 1H, H_6_), 8.17 (d, *J*_c’_ = 6.0 Hz, 1H, H_c’_), 8.12 (dd, *J*_4_ = 8.2, 1.5 Hz, 1H, H_4_), 8.02–7.80 (m, 5H, H_d,f,f’,c’’,c’’’_), 7.80–7.67 (m, 5H, H_b,b’,3,e,e’_), 7.67–7.55 (m, 9H, H_b’’,b’’’,f’’,f’’’,e’’,e’’’,d’,d’’,d’’’_), 7.51 (d, *J*_1_ = 8.8 Hz, 1H, H_1_), 7.45 (d, *J*_2_ = 1.9 Hz, 1H, H_2_), 6.49 (d, *J*_8_ = 9 Hz, 2H, H_8_), 6.30 (d, *J*_9_ = 9 Hz, 2H, H_9_), 2.73 (s, 6H, H_10_). ^13^C NMR (400 MHz, CD_3_CN) δ 139.23 (C_5_), 129.39 (C_c’_), 129.39, 129.09, 131.66, 137.9­(C_b’’,b’’’,f’’,f’’’,e’’,e’’’,d’,d’’,d’’’_), 129.30 (C_2_), 129.20 (C_1_), 129.18–129.30 (C_b,b’,3,e,e’_), 128.58 (C_4_), 127.76 (C_6_), 127.55–127.85 (C_d,f,f’,c’’,c’’’_), 124.08 (C_c_), 123.96 (C_7_), 122.13 (C_a_), 122.03 (C_a’_), 122.01 (C_8_), 121.30 (C_a’’_), 121.00 (C_a’’’_), 110.86 (C_9_), 39.79 (C_10_). UV–vis (CH_2_Cl_2_), [λ (nm) (ε, cm^–1^ M^–1^)]: 259 (110240), 294 (91510), 449 (22200), 515 (25350). E_pa_(Ru^III^/Ru^II^)­(CH_3_CN + 0.1 M TBAH): 1.46 V vs SCE. ESI-MS: 1138.13 [M-PF_6_]^+^; 496.7 [M-2PF_6_]^2+^.

#### Spectroscopic Characterization

Infrared (IR) spectra were acquired using an Agilent Cary 630 FTIR instrument fitted with an ATR MK-II Golden Gate Single Reflection accessory. Bruker DPX 400 MHz spectrometers were employed for NMR analysis, with samples prepared in CD_2_Cl_2_and CD_3_CN. Elemental analysis data were obtained using a Fisons EA-1108 CHNS–O analyzer. Lastly, ESI-MS measurements were carried out on a Thermo Quest Finnigan Navigator LC/MS system, employing acetonitrile as the eluent.

#### Optical and Photophysical Characterization

UV–vis absorption spectra were recorded using a Cary 6000i (Varian) UV–vis spectrophotometer with 1 cm quartz cells. Emission spectra were recorded using a Spex Fluoromax-4 spectrofluorometer (Horiba Jobin-Ybon, Edison, NJ, USA).

To maximize phosphorescence emission and prevent overlapping with scattered photons, appropriate excitation wavelengths were selected for each case. No fluorescent impurities were detected upon excitation in the experimental wavelength region. Furthermore, sample concentrations were adjusted to maintain an absorbance between 0.1 and 1 at λ_exc_ to avoid inner filter effects. All emission measurements were performed in 1 cm four-sided quartz cuvettes (Hellma Analytics) after purging the samples with argon to prevent triplet-state quenching by molecular oxygen.

The phosphorescence quantum yields (Φ_P_) were determined using eq 1

1 $$\Phi_{P}=\Phi_{STD}\times \left(\frac{I}{I_{STD}}\right)\times \left(\frac{1-10^{{-Abs}_{STD}}}{1-10^{-Abs}}\right)\times \left(\frac{n^{2}}{n_{STD}^{2}}\right)$$

where Φ_STD_ is the phosphorescence quantum yield of the standard where *I,* Abs and *n* represent the integrated emission band area, absorbance at the excitation wavelength, and solvent refractive index for the sample, respectively, while the subscript “STD” denotes the corresponding values for the standard. Ru­(bpy)_3_]­(PF_6_)_2_ (Φ_P_ = 0.029 in degassed CH_2_Cl_2_) was used as standard.^32^

The ability to generate ^1^O_2_ was investigated using time-resolved near-infrared phosphorescence spectroscopy, measured with a customized Fluotime 200 time-resolved spectrophotometer (PicoQuant, Berlin, Germany).^33,34^ Excitation at 355 nm was provided by a diode-pumped Nd:YAG laser (FTSS355-Q, Crystal Laser, Berlin, Germany) operating at a 1 kHz repetition rate with a pulse energy of 0.5 μJ. To eliminate residual near-infrared emissions, a 1064 nm rugate notch filter (Edmund Optics) and an uncoated SKG-5 filter (CVI Laser Corporation) were placed in the laser beam path. The emitted 1O_2_ phosphorescence was selectively detected using a 1100 nm long-pass filter (Edmund Optics) in combination with a narrow bandpass filter centered at 1275 nm (BK-1270–70 B, Bk Interferenzoptik). Detection was performed using a thermoelectrically cooled, NIR-sensitive photomultiplier tube assembly (H10330C-45-C3, Hamamatsu Photonics, Hamamatsu, Japan). Photon counting was performed using a multichannel scaler (TimeHarp 260, PicoQuant, Berlin, Germany). The temporal profile of the ^1^O_2_ phosphorescence, *S*(*t*), follows eq 2, where τ_T_ and τ_Δ_ are the lifetimes of the PS triplet state and ^1^O_2_ respectively, and *S*(0) is a pre-exponential parameter proportional to the ^1^O_2_ quantum yield (Φ_Δ_).

2 $$S\left(t\right)=S\left(0\right)\times \left(\frac{\tau_{\Delta }}{\tau_{\Delta }-\tau_{T}}\right)\times \left(e^{-t/\tau_{\Delta }}-e^{-t/\tau_{T}}\right)$$

Φ_Δ_ values were determined using eq 3, with 1*H-*Phenalen-1-one (PN, Φ_Δ_ = 1) as the reference PS.^35^

3 $$\Phi_{\Delta }=\Phi_{\Delta ,STD}\times \left(\frac{S\left(0\right)}{{S\left(0\right)}_{STD}}\right)\times \left(\frac{1-10^{{-Abs}_{STD}}}{1-10^{-Abs}}\right)$$

#### Crystallographic Data Collection and Structure Determination

Pale-yellow plate-like crystals of **2–5** were obtained via slow diffusion of diethyl ether into a CH_2_Cl_2_ solution of the respective compounds. X-ray diffraction intensity data were collected using the APEX3 software package^36^ with a Bruker D8 QUEST ECO three-circle diffractometer. This system featured a Ceramic X-ray tube (Mo Kα, λ = 0.71076 Å) and a doubly curved silicon crystal Bruker Triumph monochromator. Frame integration was performed using Bruker SAINT.^37^ The data was corrected for absorption effects using the Multi-Scan method (SADABS).^38^ The structures were solved and refined using Bruker SHELXTL.^39^ Comprehensive crystallographic parameters, and specific details of the structure determination, are provided in the Supporting Information.

#### Theoretical Calculations

All calculations were performed using the Amsterdam Density Functional (ADF) program with dispersion-corrected density functional theory at the ZORA-BLYP-D3­(BJ)/TZP level of theory. The effect of solvation in methanol was simulated by means of the conductor like screening model (COSMO) of solvation as implemented in ADF.^40–43^ Both absorption and emission spectra were computed using TD-DFT at the CAMY-B3LYP/TZ2P level of theory in CH_2_Cl_2_.

#### Photoantimicrobial Activity

The photoantimicrobial activity of the Ru­(II) complexes was tested against representative examples of three classes of pathogens: *S. aureus* (Gram-positive bacteria; ATCC 6583), *Escherichia coli* (Gram-negative bacteria; ATCC 35218), and *Candida albicans* (yeast; ATCC 18804). Microbial survival following exposure to each Ru­(II) complex as photosensitizer (PS) was determined upon light irradiation, compared with the same treatment in the dark. All experiments used exponential-phase microbial cultures derived from overnight stationary precultures. Microbial suspensions were adjusted to an optical density at 600 nm (OD_600_) of 0.18 and incubated with different concentrations of Ru­(II) complexes for 60 min. A suspension of 300 μL of each incubated sample was transferred to a 96-well plate and irradiated in a laminar flow biosecurity cabin for 60 min at room temperature. A 12W Cameo “Studio PAR64” LED light was used as a light source using a light dose of 63 J/cm^2^ with a maximum emission of 455 nm. After light treatment, each replicate was 10-fold diluted six times, seeded on Tryptone Soy Agar (TSA) plates using the track dilution method^44^ and incubated overnight at 37 °C. After 24 h, colony-forming units (CFUs) were counted for each replicate, and the survival fraction was determined by comparison with the untreated control. PS in the absence of light and light alone were used as controls. PS were generally not toxic to microorganisms in the dark (except for Ru complex **6** in *S. aureus* and *C. albicans*), and light alone did not cause cell destruction. All experiments were performed in triplicates. Effective inactivation was defined as >99.9% or 3 log_10_ CFU mL^–1^ reduction.

#### Interaction of Ru­(II) Complexes with Bacteria

A quantity of 10^8^ CFU·mL^–1^ was incubated for 60 min with 10 μM PS in PBS. After two cycles of centrifugation, washing, and resuspension, the pellet and supernatant were collected, mineralized with nitric acid, diluted with Milli-Q water, and the Ru concentration in the solutions was determined by ICP–MS (PerkinElmer NexION 5000 Multi-Quadrupole Inductively Coupled Plasma Mass Spectrometer). For the confocal studies, the same incubation procedure was used but the PS concentration was increased to 50 μM. After 60 min, 10 μL of the bacterial suspension was transferred onto a glass slide and a glass coverslip was placed on top of the drop. The images were captured using a confocal microscope (Leica DMi8; λ_exc_. = 638 nm; λ_em_ = 680–750 nm). For visualization of the samples, an apochromatic 100 objective lens and a zoom of 10 were used. The images were analyzed using ImageJ software. The same contrast/brightness was adjusted in the emission channel to facilitate visualization. The stability of the complexes in PBS was qualitatively evaluated under physiological conditions (pH 7.4, 37 °C). Overall, the complexes remained stable, with no evident precipitation or color change was observed over several hours. Although these observations are qualitative, they indicate that the complexes remained stable in PBS over the investigated time frame.

Localization studies in *S. aureus* and *E. coli* were conducted using 50 μM of **2**. As a control, we used the lipophilic porphyrin MDPyTMPyP (5-mono­(*N*-decyl-4-pyridyl)-10,15,20-tri­(*N*-methyl-4-pyridyl)-21H,23H-porphine tetrachloride), which localizes to the external bacterial wall.^45^ After 60 min of incubation, the bacterial suspensions were immobilized by mixing them with 1% agarose at 37 °C. A drop of this mixture was placed on a slide, quickly covered with a glass coverslip, and allowed to solidify. Z-stack luminescence images of the bacteria were acquired using confocal microscopy (λ_exc_ = 488 nm; λ_em_ = 582–695 nm; pinhole size = 1.0 Airy Units (AU); objective magnification = 100×; immersion medium = oil; numerical aperture = 1.40).

#### DNA Damage Assays

Genomic DNA was extracted from *E. coli* control cells using the DNeasy Blood & Tissue Kit (Qiagen). The extracted DNA was treated *in vitro* for 10 min with different Ru­(II) complexes under both dark and light conditions, followed by agarose gel electrophoresis. Additionally, after bacterial photodynamic treatment with complex **2**, genomic DNA was extracted and analyzed under the same electrophoresis conditions. The gel was stained with ethidium bromide and visualized using a Bio-Rad ChemiDoc MP Imaging System (Bio-Rad, Hercules, CA, USA).

#### Interaction of Ru­(II) Complexes with Human Fibroblasts

The dark- and photocytotoxicity of compounds **2** and **6** against human fibroblast cells were tested using the resazurin assay. Human fibroblast cell line 1BR3G (ECACC 90011801) was seeded in 96-well plates at a cell density of 2 × 10^4^ cells/well using fresh Dulbecco’s Modified Eagle’s Medium–high glucose (DMEM) supplemented with stable pyruvate +1% fetal bovine serum (FBS) and incubated under 5% CO_2_ for 24 h at 37 °C. The old medium was replaced with fresh medium containing different concentrations of compounds **2** or **6** (0,1, 1, 10, and 100 μM) and cultured for 4 h. Thereafter, irradiation was performed for 1 h using the 12 W Cameo “Studio PAR64” LED light (455 nm emission and light dose of 63 J/cm^2^), while the control group was maintained in the dark. After the treatment, cells were incubated at 37° and 5% CO_2_ for a total of 24 h since initial exposure to the compounds. Subsequently, a resazurin assay was performed to determine cell viability. Each experiment was repeated 3 times, and the data are represented as the mean ± SD.

## Results and Discussion

### Synthesis of Ru­(II) Complexes

Ru­(II) complexes **2–6** were prepared by substituting the two chlorido ligands in the corresponding starting compounds, **1** or **1′** with the desired nitrogen bidentate ligands L2-L5, as shown in Scheme 1. The pyridinepyrazole ligand L2 was synthesized according to a method described in the literature.^31^ The iminopyridine and iminoquinoline ligands L3-L5 were synthesized via a condensation reaction between the corresponding aldehydes and amines in MeOH. Complex **2** was obtained by the reaction between the isolated L2 Iigand and **1** in methanolic solution, complexes **3–5** were prepared by adition of **1** ^14^ to the in situ synthesized L3-L5 ligands, followed by the addition of NH_4_PF_6_. Complex **6** was obtained from the reaction of ligand L5, also formed in situ, with one equivalent of compound **1’**.^14^ Complexes **2**, **3**, **5** and **6** were isolated as PF_6_^–^ salts without purification, while for complex **4**, a column chromatography led to the pure compound.

### Structural Characterization

The complexes were fully characterized by NMR, IR and UV–vis spectroscopies, elemental analysis, and electrospray ionization mass spectrometry. Redox potentials were determined by cyclic voltammetry (see Supporting Information). Crystal structures of **2–5** were determined by X-ray diffraction analysis. Unfortunately, the different attempts to crystallize compound 6, were unsuccessful. Their molecular structures are illustrated in Figure 1, and the principal crystallographic data and selected bond lengths and angles are provided in the Supporting Information (Tables S1 and S2).

**Figure 1:**
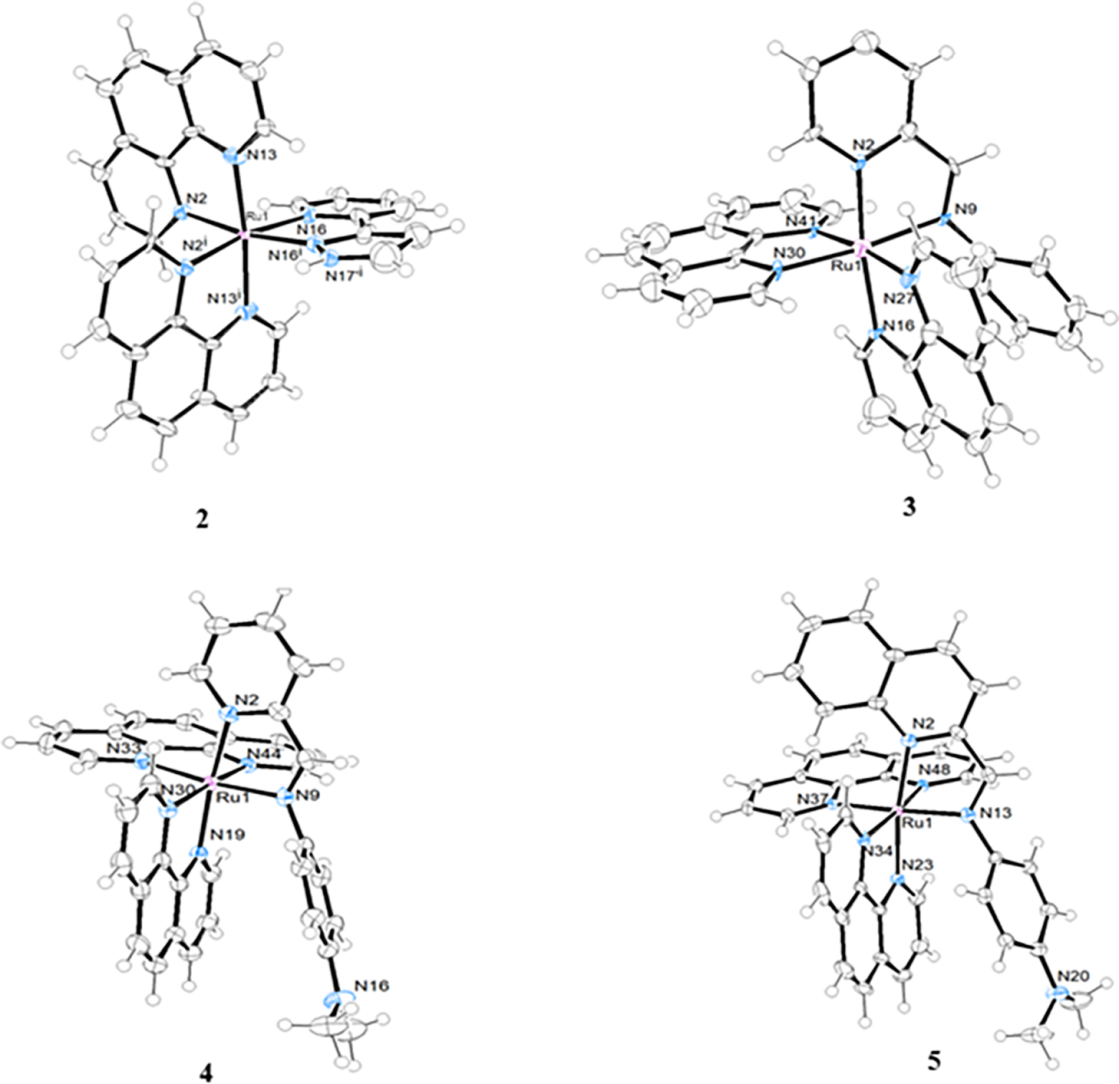
ORTEP plots and labeling schemes for the cations of complexes **2–5**.

In all complexes, the Ru metal centers displayed a distorted octahedral coordination sphere, with the bidentate ligands binding to the metal via their nitrogen atoms. This spatial distortion stems from the geometric constraints of the L1–L6 chelates, which exhibit angles of less than 90° upon coordination. Consequently, the remaining coordination angles expanded beyond the 90° expected for a perfect octahedron. All structural parameters, including bond lengths and angles, are in good agreement with typical values for related ruthenium complexes.^46,47^

It is worth mentioning that the phenyl rings in ligands L3–L5 are not coplanar with the pyridine (**3**, **4**) or quinoline (**5**) aromatic rings of the same ligand, showing torsion angles (C=N–C_ar_-C_ar_) of 59.74^ο^ (**3**), 57.03^ο^ (**4**) and 73.71^ο^(**5**) and displaying π-staking interactions with the phenanthroline aromatic rings of the same molecule (see Figure 1). The different torsion angles observed in compound **5** could be due to the intermolecular hydrogen bonds observed between the phenyl ring of the iminoquinoline ligand and the F atoms of one PF_6_^–^ anion (H18–F6S = 2.774 Å and H25–F5S = 2.494 Å), which were not observed in compounds **3** and **4**. (see Figure S1). X-ray diffraction analysis of the four complexes reveals the coexistence of both the Δ and Λ enantiomers, matching observations reported for similar systems in the literature.^14^

The supramolecular structures of the compounds show solvent molecules and anions PF_6_^–^ between the Ru­(II) cations. Rac-**2** displays π-stacking interactions between L1 aromatic rings of neighboring molecules. Unlike complexes *rac-* **3** and **4**, the structure of rac-**5** no displays π-stacking interactions between the phenyl ring of the iminoquinoline ligand L5 with one of the L1 rings of the neighboring molecule. Different views of the packing are shown in Figure S2.

The experimental geometries agree with those optimized using computational methods at the dispersion-corrected ZORA-BLYP-D3­(BJ)/TZ2P level in MeOH (Figure S3 and Table S3 in the Supporting Information).

### Spectroscopic Characterization

In the IR spectra of all complexes, the bands observed near 3100 cm^–1^ correspond to the aromatic ν­(=C–H) stretching vibrations. Additionally, signals in the 1600 −1400 cm^–1^ range are attributed to the ligand-centered ν­(C=C) stretching modes, while the peaks located between 1300 and 1110 cm^–1^ are characteristic of in-plane δ­(C–H) bending.

In the case of complex **2** the peak around 3300 cm^–1^ corresponds to the υ­(−N–H) of L2. For complexes **3–6** the bands corresponding to the stretching vibration of the azomethine group, ν­(C=N), appear around 1600–1650 cm^–1^. All spectra show a peak around 845 cm^–1^ that can be assigned to the anion PF_6_^–^. The IR spectra of **2** and **6** are displayed in Figure S4.

1D and 2D ^1^H NMR spectra for complexes **2–6** were acquired in either CD_2_Cl_2_ or CD_3_CN, and are presented in Figures S5–S9. The pattern of resonances indicates that all the complexes are asymmetric molecules, which was in excellent agreement with the solid-state structures. ^1^H NMR of **2** and **3** showed one sets of resonances in the aromatic region corresponding to the protons of the L1-L3 ligands (Figures S5 and S6). In the case of complex **2**, it is worth mentioning the singlet observed at δ = 11.5 ppm, that is assigned to the pyrazolic proton (N–H). As expected, the ^1^H NMR spectra of **4–6** (Figures S7–S9) displayed two distinct sets of resonances, one in the aromatic region attributable to the ring protons of ligands, and the other in the aliphatic region, at δ = 3.50–2.5 ppm, due to the CH_3_ substituents of the imine ligands L4 and L5. The presence of asymmetric ligands (L2-L5) reduces molecular symmetry, leading to an increased number of resonances. This indicates distinct chemical environments for all protons of the two L1 (in complexes **2–5**) or L6 (in complex 6) ligands that are chemical and magnetically nonequivalent. 2D COSY and NOESY NMR correlations enabled the assignment of the nonequivalent protons, whereas signal overlap was observed in some instances.

The ESI-MS of compound **2** reveals peaks corresponding to [M-(L2)-PF_6_^–^]^+^ and [M-(L2)-2PF_6_^–^]^2+^, whereas compounds **3–6** display peaks corresponding to [M-PF_6_]^+^ monocations and [M-2PF_6_]^2+^ dications. The ESI-MS spectra of the compounds are shown in Figure S10.

### Optical and Photophysical Properties

All the optical and photophysical results are collected in Table 1. To determine the optical properties of compounds, UV–visible spectra were recorded in CH_2_Cl_2_ and MeOH (Figures 2 and S11). All complexes displayed similar absorption profiles, with a group of ligand-based π- π* bands below 350 nm and a series of dπ­(Ru)-π*­(L) MLCT transitions, along with other transitions involving intraligand π–π * bands, between 454 a 515 nm (see infra).

**Table 1:** Photophysical Data of Ru­(II) Complexes **2-6** ^a^

complexes	solvent	λ_abs_ (nm)	ε (M^–1^ cm^–1^)	λ_P_ (nm)^b^	Φ_P_ ^c^/10^–3^	τ_P,Ar_ (ns)^d^	τ_P,air_ (ns)^d^	*k*_q_^O2^ (M^–1^ s^–1^)	*P*_T_^O2^	Φ_Δ_ ^e^	Φ_Δ_/P^T^ ^O2^ (%)
**2**	CH_2_Cl_2_	450	13 230	590	0.6	120	105	3.0 × 10^8^	0.13	0.13	100%
	MeOH	484		620	0.2	56	41	3.0 × 10^9^	0.28	0.28	100%
**3**	CH_2_Cl_2_	475	13 210	584	0.5	163	123	5.1 × 10^8^	0.25	0.04	16%
	MeOH	472		594	0.2	260	109	2.4 × 10^9^	0.58	0.05	9%
**4**	CH_2_Cl_2_	470	25 150	590	0.4	152	100	8.7 × 10^8^	0.34	0.30	88%
	MeOH	467		590	0.2	250	105	2.5 × 10^9^	0.58	0.27	47%
**5**	CH_2_Cl_2_	509	19 140	590	0.3	127	112	2.7 × 10^8^	0.12	0.11	92%
	MeOH	504		610	0.1	301	150	1.5 × 10^9^	0.50	0.15	30%
**6**	CH_2_Cl_2_	518	25 350	613^c^	3.5	1610	620	7.0 × 10^8^	0.61	0.08	13%
	MeOH	513		617	1.2	1330	269	1.3 × 10^9^	0.84	0.09	11%

a λ_abs_, absorption band maximum; ε, molar absorption coefficient; λ_P_, phosphorescence band maximum; Φ_P_, phosphorescence quantum yield; τ_P,Ar_, phosphorescence lifetime in argon-saturated solutions; τ_P,air_, phosphorescence lifetime in air-saturated solutions; *k*_q_^O2^, rate constant for oxygen quenching of the complexes’ triplet state; *P*_T_^O2^, efficiency of oxygen trapping of the complexes’ triplet state; Φ_Δ_, singlet oxygen quantum yield; Φ_Δ_/P_T_^O2^, efficiency of singlet oxygen formation per oxygen quenching event.

b Excited at 450 nm.

c Using [Ru­(bpy)_3_]^2+^ as standard.

d Excited at 375 nm.

e Using 1*H-*phenalen-1-one as standard.

**Figure 2:**
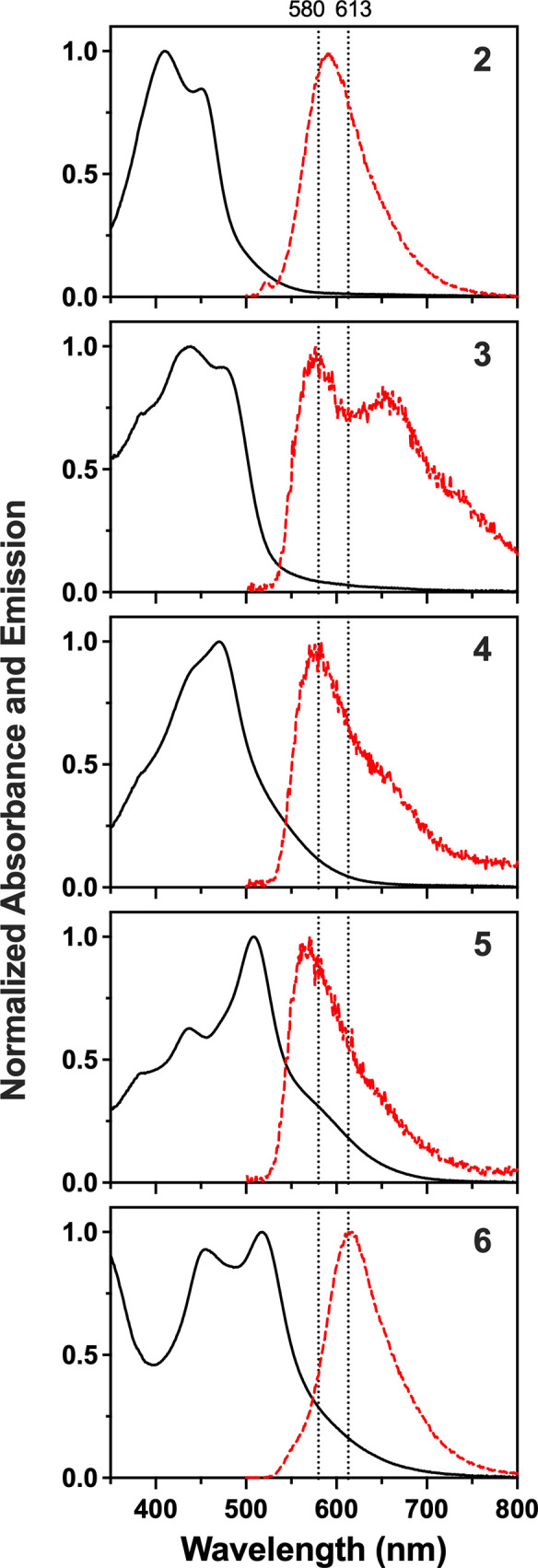
Normalized Absorption and emission spectra of Ru­(II) complexes **2–6** in CH_2_Cl_2_. As a visual aid, vertical dashed lines mark 580 and 613 nm, corresponding to the main emission maxima associated with ligand L1 (**2–5**) and ligand L6 (**6**), respectively.

In CH_2_Cl_2_, a red shift of the lowest-energy band was observed in complex **3** in comparison to **2**, probably due to the stabilization of the LUMO by the iminopyridine moiety, which reduces the HOMO–LUMO energy gap. Iminopyridine ligands are known to have lower-lying π* orbitals compared to pyridinepyrazole ligands due to their superior π accepting capability. This is observed in the Figure S12, in which energies of LUMO and LUMO + 1 for **2** are −2.19 and −2.09 eV, whereas those for **3** are −2.57 and −2.22 eV, respectively.

In compound **6**, the presence of the L6 ligands induced a bathochromic shift of the lowest-energy absorptions with respect to the analogous complex **5** bearing the L1 ligand, which is consistent with the weaker σ-donor capacity of the L1 ligand and with the weaker π-acceptor character of L6. Such shift was further supported by the energies of the involved Frontier orbitals involving H-2 → L in case of complex **5** and H → L for **6**.

The above absorption spectra were further rationalized by TD-DFT calculations at CAMY-B3LYP/TZ2P level in CH_2_Cl_2_ (Figures S13–S15). Both HOMO and LUMO orbitals for the five complexes under analysis, together with their HOMO–LUMO gaps appear in Figure S16 including the energies of the frontier molecular orbitals in the main bands.

Despite the computed electronic transitions were slightly blue-shifted compared to the experimental values, the red-shifting trend going from **2** to **3** to **4** to **5** and to **6** was quite well reproduced. It must be taken into account that computations have been performed on the dicationic Ru complexes without the counteranions. The strongest lowest-energy bands correspond to the excitation from d orbitals of Ru to antibonding π orbitals of the ligand, i.e., metal-to-ligand charge transfer. Noticeably, there is also some intraligand π–π* transfer in all cases (Figures S13–S15). It must also be noted that the HOMO–LUMO bands, despite also present, show a quite low intensity in all cases. However, they followed the expected red-shift trend, i.e., HOMO–LUMO band increases from 415 to 482 to 503 nm from **2** to **3** to **4**, respectively. In this case, there was a clear difference between the HOMO of complexes **2** and **3**, with the density mainly based on the d orbitals of Ru atom, thus implying MLCT transitions to the π* LUMO. At difference, in case of systems **4**, **5** and **6**, density is located on one of the ligands, thus this transition clearly involving intraligand π–π* between two different ligands of the Ru complex, without the participation of the d orbitals of Ru. In addition, the HOMO–LUMO energy gap was also reduced in the same order, **2** > **3** > **4** > **5** > **6** (Supporting Information), with a destabilization of the HOMO and a stabilization of the LUMO from **2** to **6**.

The solvent had a minor influence on the position and intensity of the absorption bands of all complexes, with the exception of complex **2**, for which the visible bands shifted to the red upon increasing the solvent polarity and proticity (Figure S11).

On another hand, we evaluated the photostability of complexes **2–6** in aerated CH_2_Cl_2_ and MeOH under blue-light irradiation (Figures S17 and S18). In CH_2_Cl_2_, all complexes showed <10% photobleaching at 45 J cm^–2^; in MeOH, only complex **4** exceeded 10% under the same light fluence. Notably, the two most promising photoantimicrobials (see below), **2** and **6**, were essentially photostable (absorbance loss <2%).

All complexes showed broad yet very weak emission in degassed CH_2_Cl_2_ (Figures 2 and S19) with maxima in the 570–620 nm range. Complexes **2–5**, bearing two L1 ligands and different *N*-donor based ligands, exhibited a maximum around 570 nm and a shoulder (a second maximum in the case of complex **3**) at 660 nm, corresponding to two different emissive states as a consequence of heteroleptic coordination. In contrast, the emission spectrum of complex **6**, lacking the L1 ligand, was very different, showing the maximum at 620 nm. The emission spectra computed at the CAMY-B3LYP/TZ2P level in CH_2_Cl_2_ confirm the blue-shifted emission bands of compounds **2** and **4** compared to **5** and **6** (Figure S20).

Consistent with the distinct emission behavior, we found that the emission decay kinetics was also very different for complexes **2–5** (average lifetime 140 ± 25 ns in degassed CH_2_Cl_2_ solutions) compared to complex **6** (1.6 μs; ∼10-fold longer). Representative decays are shown in Figure S21. These values are consistent with literature data for [Ru­(L1)_3_]^2+^ (∼440 ns) and [Ru­(L6)_3_]^2+^ (∼1.7 μs) in deaerated acetonitrile,^48,49^ supporting assignment of the emissions to the L1-and L6-based systems, respectively. The same trend was observed for the emission quantum yields (Φ_P_), which ranged from 3 × 10^–4^ for complex **5** to 3.5 × 10^–3^ for complex **6** in degassed CH_2_Cl_2_ solutions, one and 2 orders of magnitude, respectively, smaller than for [Ru­(bpy)_3_]­(PF_6_)_2_.^32^ Replacing the nonpolar solvent CH_2_Cl_2_ by MeOH induced a clear shortening of the emission lifetime for complexes **2** and **6**, while the opposite effect was observed for complexes **3–5**. However, the Φ_*P*_ values were lower for all compounds.

### Oxygen Quenching and Generation of ROS

For all compounds, the emission could be quenched by oxygen, resulting in shorter decay lifetimes (Figure S21). From the lifetime values and the solubility of oxygen in the different solvents, the quenching rate constants (*k*_q_^O2^) were calculated, as well as the fraction of triplets trapped by oxygen (*P*_T_^O2^). For compounds **2–5** in CH_2_Cl_2_, the combination of short lifetimes and small *k*_q_^O2^ values lead to oxygen trapping efficiencies in the 12%–34%. Compound **6** was, again, an exception, 61% of the triplet excited states being trapped by oxygen in air-saturated solutions. In MeOH the quenching rate constants were higher, resulting in higher efficiency of oxygen trapping (28%–54% for complexes **2–5** and 84% for complex **6**).

Oxygen quenching of excited states can lead to the production of the cytotoxic ^1^O_2_. The quantum yield of ^1^O_2_ production (Φ_Δ_) was determined by time-resolved near-infrared phosphorescence spectroscopy. All compounds produced ^1^O_2_, albeit the Φ_Δ_ values are smaller than the *P*_T_^O2^ for some complexes and solvents (Figure S22). This indicates that either the production of the complexes’ triplet excited occurs with quantum yield smaller than unity in such cases, or the oxygen quenching event leads to the production of species other than ^1^O_2_ (e.g., the superoxide radical anion) or just to physical deactivation of the excited states.^50^ It is worth noting that only for complex **2** the ratio of efficiencies is close to 100% in both CH_2_Cl_2_ and MeOH. This behavior may be attributed to the structural rigidity of its pyridine–pyrazole ligand, which stabilizes the excited state by suppressing nonradiative decay pathways. In turn, a lower Φ_*p*_ value is observed for complex **3** compared to complex **4**, despite both containing one iminopyridine-type ligand and two phenanthrolines, suggesting that factors other than ligand rigidity may play a role. The enhanced efficiency of complex **4** could result from a positive contribution of intraligand transitions in its excited state resulting from the electron-donating effect of the *p-*dimethylamino substituent. Similarly, complex **6** exhibited lower singlet oxygen production compared to complex **5**; in this case, the presence of the less rigid L6 ligands (versus L1 ligands) appears to reduce efficiency, indicating that ligand rigidity exerts a stronger influence than the presence of intraligand bands. Production of superoxide radical anion (O_2_^•–^) was ascertained using dihydroethidium (DHE) as a selective fluorescent trap. DHE oxidation was monitored under our irradiation conditions with appropriate controls (Figure S23). As expected, all complexes produce O_2_^•–^ upon illumination, albeit with different levels. Notably, the complexes **2** and **6**, which showed the highest photoantimicrobial activity, produced the highest levels of O_2_^•–^, suggesting a role of electron transfer in the photoantimicrobial mechanism.

Finally, guided by our theoretical calculations, we can propose a schematic energy–transfer diagram that summarizes the relevant Ru-centered/ligand-centered (LC) and MLCT excited states involved in ROS formation (Figure S24).

### Photoantimicrobial Activity

The antimicrobial activity of Ru­(II) complexes is shown in Figure 3. Irradiation in the absence of the PS had no significant effect on the microorganisms’ viability. Likewise, no activity was observed for any compound in the absence of light, with the notable exception of complex **6** for *S. aureus*.

**Figure 3:**
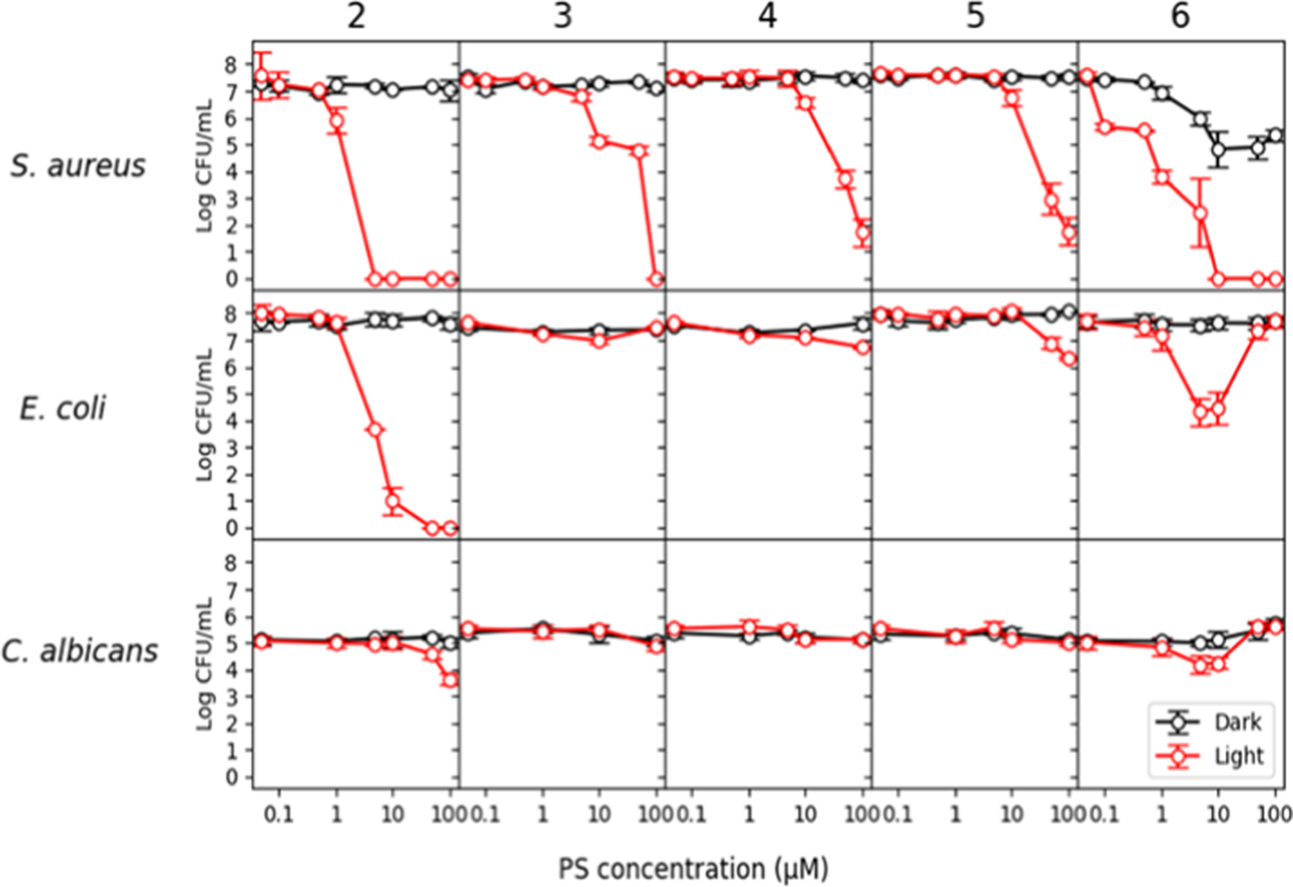
*Staphylococus aureus*, *Escherichia coli* and *Candida albicans* photodynamic inactivation (PDI) using increasing concentrations of the Ru­(II) complexes **2–6** exposed to 63 J/cm^2^ of blue light (455 nm, red symbols). Black symbols correspond to the dark toxicity. The concentrations tested were 0.05, 0.1, 0.5, 1, 5, 10, 50, and 100 μM.

While all complexes were active against *S. aureus*, complex **6** showed the highest efficiency, but it also showed the highest dark toxicity. The second most-active compound was complex **2**, achieving 7 log_10_ (99.99999%) reduction of Colony Forming Units (CFU) at 5.0 μM. The results were different for *E. coli*, where only complex **2**, and to a lesser extent complexes **5** and **6̧** showed activity. Susprisingly, complex **6** showed 3-log_10_ reduction at 5 and 10 μM, but was ineffective at higher concentrations. Based on Student’s *t* tests (significance threshold *p* < 0.05), *Candida albicans* showed no statistically significant response to any compound tested, except complex **2** at concentrations ≥50 μM. This may be explained by the low permeability of the rigid fungal wall, composed of a β-glucan-chitin skeleton and mannoproteins.^51^

Mechanistic insight into the above results was obtained by uptake studies, confocal microscopy, and DNA photodamage studies. The association of Ru­(II) complexes with bacteria, including cell–wall adsorption and cellular internalization, was assessed by measuring the Ru concentration in the bacteria after 60 min of incubation. The results are shown in Tables 2 and S4.

**Table 2:** Concentration of Ru­(II) Complexes in the Bacteria. The Concentration of the Different Ru Complexes was of 10 μM Using Pre-Prepared Stock Solutions in DMSO at 1 mg/mL

complex		2	3	4	5	6
[Ru]/μg L^–1^	*S. aureus*	2.06	1.58	0.53	1.59	105
	*E. coli*	1.39	<0.5	<0.5	0.96	82

The results showed that all complexes associate to *S. aureus*, consistent with the photoantimicrobial activity observed (Figure 3). Notably, complex **6** exhibited the strongest association, 50 times higher than the others, but this did not result in a corresponding 50-fold increase in activity. In *E. coli*, complexes **2**, **5**, and most notably complex **6**, which again showed a 50-fold stronger association, exhibited the highest binding affinity. As in *S. aureus*, these results are broadly in agreement with their photoantimicrobial activity. The overwhelming association of complex **6** may be accounted for by its structure. Being the most hydrophobic among the complexes tested, it is poorly soluble in the aqueous phase and sticks to the bacterial cell wall. The high hydrophobicity prevents it from crossing the cell membrane. The loss of activity observed at the highest concentrations (Figure 3) may be the result of aggregation.

Being complex **2** the compound with the broadest activity, additional studies were conducted to understand its underlying mechanism. Confocal microscopy confirmed that **2** was internalized by both *S. aureus* and *E. coli* (Figure 4). A quantitative analysis of the z-stack luminescence images, considering multiple subdiffraction-sized emitters, further revealed that it localizes within the cytoplasm of both species, in contrats to the lypophilic porphyrin control MDPyTMPyP, which localized on the external bacterial wall.^45^

**Figure 4:**
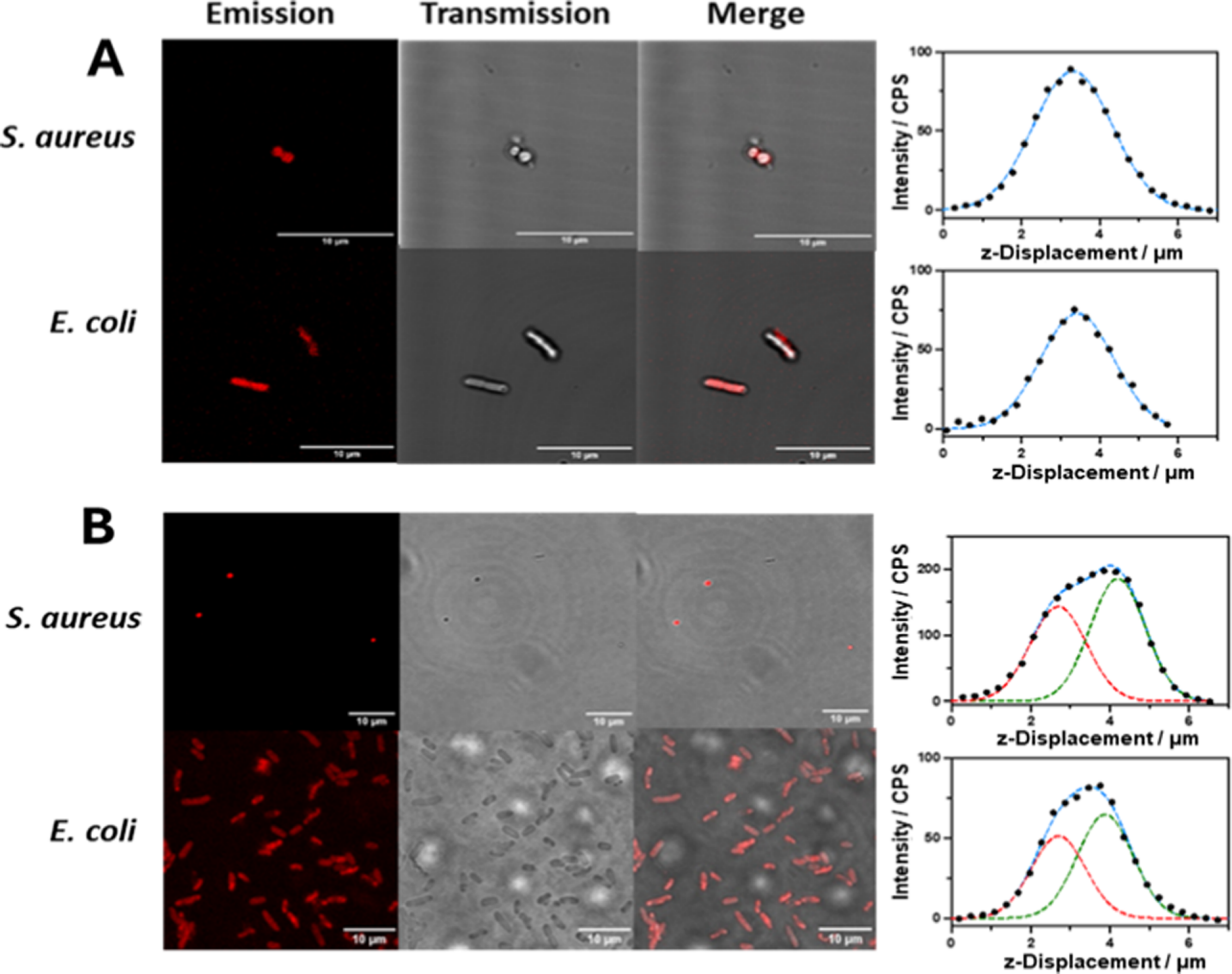
Representative confocal microscopy images showing the emission channel of *S. aureus* and *E. coli* incubated with (A) 50 μM of complex **2**, and (B) 50 μM MDPyTMPyP porphyrin for 60 min in the dark. The graphs on the right show luminescence intensity profiles measured across the axial direction for each set of images (black dots). The intensity profiles for MDPyTMPyP were best fitted using two Gaussian functions (red and green dashed lines), whereas a single Gaussian function (blue dashed lines) sufficed for complex **2**, indicating distinct localization of the two chromophores within the bacterial species.

We investigated the interaction of the Ru­(II) complexes with calf thymus DNA (ctDNA) and assessed whether DNA binding influences their photochemical behavior and downstream genotoxic effects. First, UV–Vis absorption spectroscopy revealed that complex **2** undergoes a substantial red shift in the presence of ctDNA, consistent with an intercalative binding mode (Figure S25),^52^ whereas the remaining Ru complexes showed a much smaller spectral perturbation, indicating weaker and/or less extensive DNA association. Next, we examined whether ctDNA modulates the photodegradation of the complexes and found that adding ctDNA produces no statistically significant changes in the photodegradation profiles (Figure S26), suggesting that DNA does not measurably alter the ligand photorelease process under the conditions tested. Finally, we evaluated DNA integrity using two complementary damage assays: (i) direct exposure of purified genomic DNA to the Ru complexes (Figure 5a,b), and (ii) treatment of bacterial cells with **2** followed by genomic DNA extraction and analysis (Figure 5c,d). The agarose gel electrophoresis images revealed the effects of the different complexes on genomic DNA under dark (Figure 5a) and light (Figure 5b) conditions. In the dark, all samples displayed a clear, crown-shaped band suggesting that the DNA remains largely intact in the absence of light activation. Upon irradiation, however, a reduction in band intensity was observed, with complex **2** producing the weakest DNA signal, consistent with a higher degree of disruption of genomic DNA integrity.

**Figure 5:**
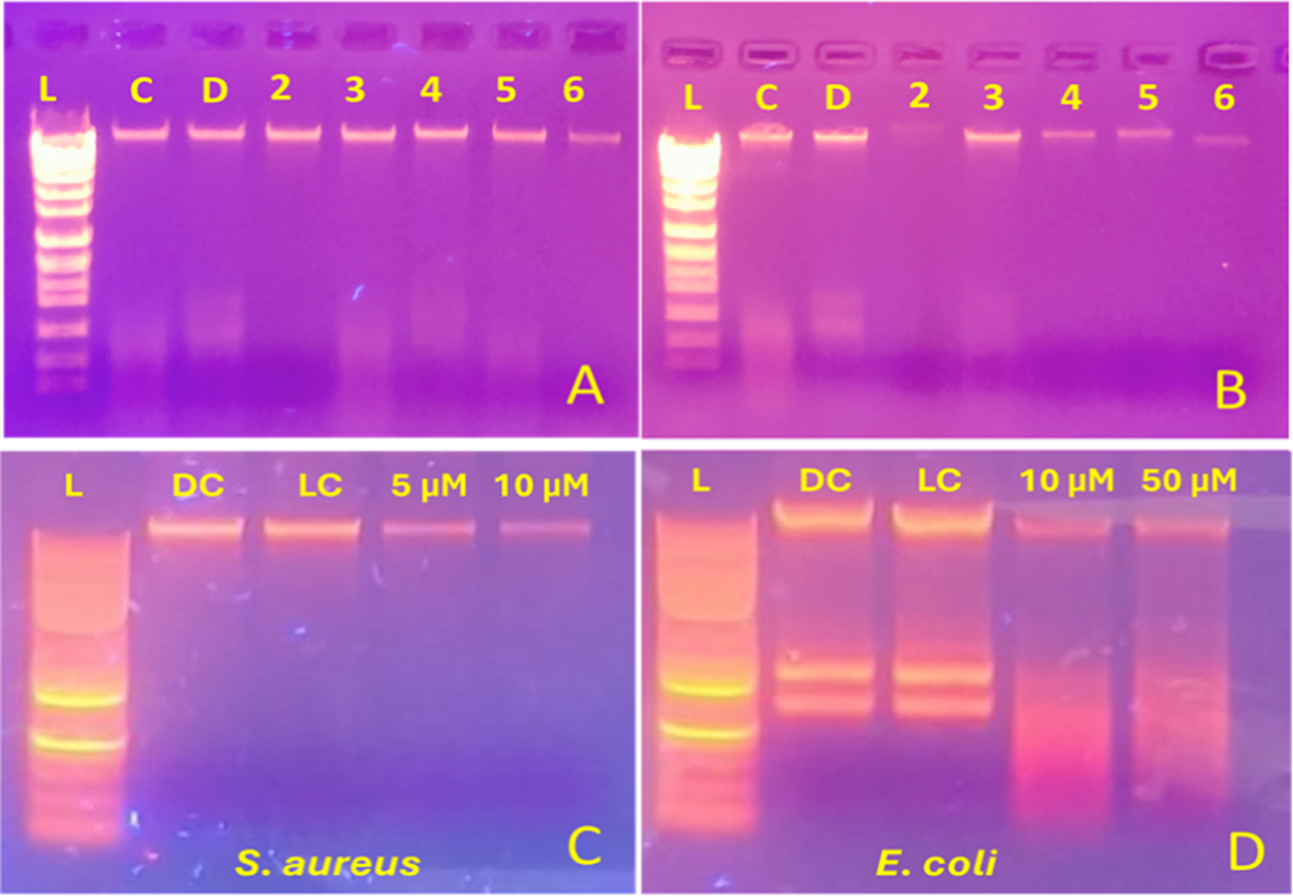
Agarose gel electrophoresis of: (A) in vitro DNA treated with Ru complexes under dark conditions; (B) in vitro DNA treated under light exposure: (Lane L—ladder of known base pairs DNA size; Lane C—control; Lane D—DMSO control; Lanes 2–6—treatment with 10 μM of complexes **2–6**, respectively); (C) DNA extracted from phototreated *S. aureus* cells with 5 μM and 10 μM of complex 2 and (D) DNA extracted from phototreated *E. coli* cells with 10 μM and 50 μM of complex 2. (Lane L—ladder of known base pairs DNA size; Lane DC—dark control; Lane LC—light control).

Agarose gels of extracted DNA from phototreated bacteria with complex **2** showed DNA damage in both *S. aureus* and *E. coli* species (Figure 5c,d). The lower intensity of the band relative to the controls reveals a decrease of genomic DNA concentration upon the photodynamic treatment. In addition, RNA degradation was also observed in *E. coli*, as reflected by a characteristic smear corresponding to the fragmented nucleic acid (Figure 5d).

### Interaction with Human Fibroblasts

A key requirement for photoantimicrobials intended for human and animal use is to establish their safety. To this end, the dark- and phototoxicity of complex **2** and complex **6** against human fibroblasts was studied in vitro using the resazurin cell survival assay (Figure 6). Complex **2** showed negligible cell death in the dark within the range of administration dosage, indicating excellent biocompatibility. However, blue light irradiation induced cell phototoxicity at the highest concentration (100 μM), showing a significant decrease of viability. On the other hand, complex **6** showed some cellular toxicity in the dark at the highest concentration with a decrease of viability (80%). After exposure to light at λ = 455 nm, significantly reduced cell viabilities were observed in a concentration-dependent manner. It is worth noting that the decrease in viability at the highest concentration of complex **6** was less pronounced than expected, which may reflect the poor aqueous solubility, and potential aggregation at higher concentration.

**Figure 6:**
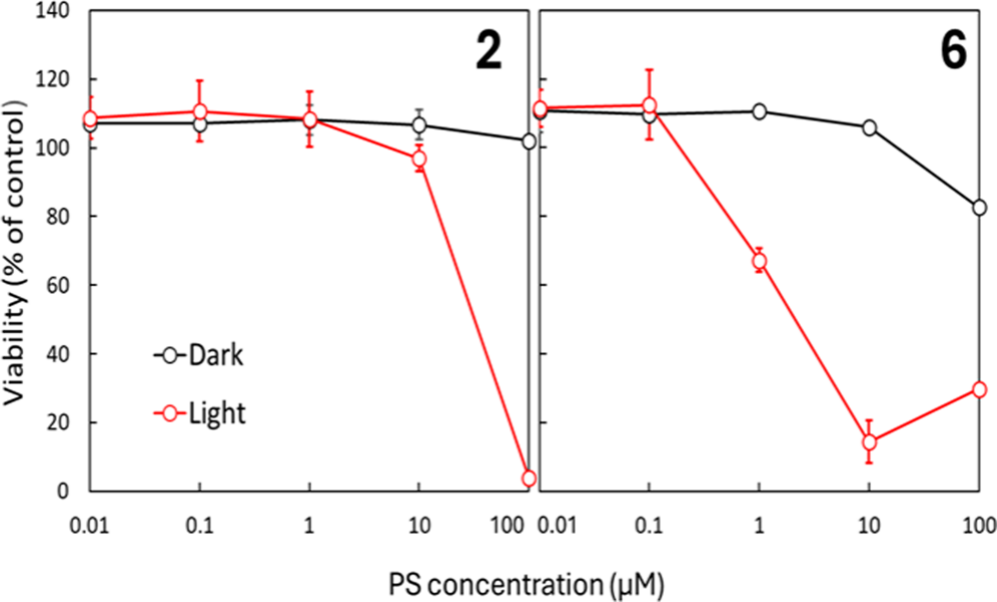
Dark and light cytotoxicity of complexes **2** and **6** against human fibroblast cells by the Resazurin assay. Data are expressed as means ± SD based on three measurements.

## Conclusions

A series of heteroleptic Ru­(II) N-donor complexes (**2–6**), incorporating symmetric *N*-bidentate ligands such as 1,10-phenanthroline (L1) and 4,4′-diphenyl-2,2′-bipyridine (L6), together with asymmetric *N*-bidentate ligands including pyridine–pyrazole (L2), iminopyridines (L3 and L4), and iminoquinoline (L5), have been successfully synthesized and characterized. The strategic incorporation of these asymmetric N-donor ligands emerges as a key design principle in this study. Beyond simply breaking molecular symmetry, these ligands enable a multimodal optimization of the Ru­(II) complexes, balancing light-harvesting properties, singlet oxygen production, and cellular uptake through the fine-tuning of the complex’s electronic and amphiphilic character. The complexes adopt distorted octahedral geometries and are weakly luminescent. Upon irradiation, they photosensitize the production of different ROS, including ^1^O_2_ and O_2_^•–^, with efficiencies that depend on the Ru complex, and they showed photobactericidal activity against *S. aureus*. The results demonstrated that **2** is the complex with the highest potential for aPDT. The synergy between its two 1,10-phenanthroline ligands and the extended pyridine–pyrazole ligand creates an optimized coordination environment that endows this complex with appropriate physicochemical, optical, photophysical and photochemical properties, particularly high hydrophilicity, enhanced conjugation and high singlet oxygen production ability. Complex **2** photoinactivates both Gram-positive and Gram-negative bacteria with high efficiency, owing to its extensive internalization into the bacterial cytoplasm, which enables genomic DNA damage. Cytotoxicity assays on human fibroblasts showed that complex **2** is nontoxic at the concentrations used for antimicrobial purposes, which underscores its therapeutic safety.

Altogether, the unique synergy of strong photophysical performance, broad-spectrum antibacterial activity, efficient bacterial uptake, and high dark biocompatibility highlights complex **2** as a highly promising scaffold for advanced Ru­(II)-based PSs. By directly correlating molecular structure with biological function, these findings establish new design principles for efficient and selective aPDT agents and represent a key step toward innovative antimicrobial strategies to combat antibiotic.

## Supplementary Material



## Notes

Crystallographic information, electrochemical characterization, computational data. IR, NMR, absorption and emission spectra, time-resolved emission kinetic traces of complexes. Determination of ^1^O_2_ formation quantum yield (Φ_Δ_). This material is available free of charge on the ACS Publications Web site (PDF)
