# Supplementary material for: Enhancing the Antimicrobial Photodynamic Activity of Heteroleptic Ruthenium(II) Complexes: Role of Asymmetric N‑Donor-Based Ligands
Source: Inorg Chem. 2026 Jun 26;65(27):15389–401. doi: 10.1021/acs.inorgchem.6c00420 (PMC13370854; doi:10.1021/acs.inorgchem.6c00420)

SUPPORTING INFORMATION

Enhancing the antimicrobial photodynamic activity of heteroleptic ruthenium(II) complexes: role of asymmetric *N*-donor-based ligands

Abir Mesra-Brahmi,[a,b] Sofia Martínez-Olmo,[a] Isabel Guerrero,[c] Xavier Fontrodona,[a] Jordi Poater,[d,e] Ariadna Verdaguer,[b] Maria Auset Vallejo,[b] Roger Bresolí-Obach,[b] Santi Nonell,[b,e] * Rosario Núñez,[c] * and Isabel Romero[a] *

[a] Departament de Química and Serveis Tècnics de Recerca, Universitat de Girona, C/ M. Aurèlia Capmany, 69, E-17003 Girona, Spain.
[b] Institut Químic de Sarrià, Universitat Ramon Llull, 08017 Barcelona, Spain
[c] Institut de Ciència de Materials de Barcelona, ICMAB-CSIC, Campus UAB, E-08193 Bellaterra, (Cerdanyola del Vallés), Barcelona, Spain.
[d] Departament de Química Inorgànica i Orgànica & IQTCUB, Universitat de Barcelona, Martí i Franquès 1-11, 08028 Barcelona, Spain.
[e] Catalan Institution for Research & Advanced Studies, ICREA, Pg. Lluís Companys 23, 08010 Barcelona, Spain

*Authors to whom correspondence should be addressed. I.R.: marisa.romero@udg.edu; R.N.: rosario@icmab.es; S.N.: santi.nonell@iqs.url.edu

1. Crystallographic information

2. Electrochemical characterization

3. Supplementary Tables

Table S1. Crystallographic data for the X-ray diffraction of **2-5** complexes.

Table S2. Selected bond lengths (Å) and angles (°) for **2-5** complexes.

Table S3. List with Cartesian coordinates (in Å), ADF electronic energies (in parentheses, in kcal mol^{-1}), of all complexes optimized at ZORA-BLYP-D3(BJ)/TZ2P level in methanol (COSMO). Charge of all complexes is +2, counterions have not been considered.

Table S4. Concentration of Ru(II) in *S. aureus* and *E. coli* bacteria.

4. Supplementary Figures

Figure S1. Intermolecular hydrogen bonds observed in compound **5**.

Figure S2. Packing of **2** along a) *b* and b) *c* axis; packing of **3** along c) *b* and d) *c* axis; packing of **4** along e) *a* axis; packing of **5** along f) *b* axis.

Figure S3. Geometries of the ruthenium clusters under analysis (without counterions), optimized at ZORA-BLYP-D3(BJ)/TZ2P level in methanol (COSMO). Bond lengths Ru-N are also enclosed (in Å).

Figure S4. IR spectra of complexes **2-6**.

Figure S5. NMR spectra of $[Ru(phen)_2(pypz\text{-}H)](PF_6)_2$ **2**, 400 MHz, CD_2Cl_2: a) ^{1}H-NMR; b) COSY; c) NOESY; d) ^{1}H-^{13}C HSQC; e) ^{1}H-^{13}C HMBC.

Figure S6. NMR spectra of $[Ru(phen)_2(L1)](PF_6)_2$ **3,** 400 MHz, CD_2Cl_2: a) ^{1}H-NMR b) ^{13}C-NMR; c) COSY; d) NOESY; e) ^{1}H-^{13}C HSQC; f) ^{1}H-^{13}C HMBC.

Figure S7. NMR spectra of $[Ru(phen)_2(L2)](PF_6)_2$ **4,** 400 MHz, CD_2Cl_2: a) ^{1}H-NMR b) ^{13}C-NMR; c) COSY; d) NOESY; e) ^{1}H-^{13}C HSQC; f) ^{1}H-^{13}C HMBC.

Figure S8. NMR spectra of $[Ru(phen)_2(L3)](PF_6)_2$ **5,** 400 MHz, CD_2Cl_2: a) ^{13}C-NMR; b) COSY; c) NOESY; d) ^{1}H-^{13}C HSQC; e) ^{1}H-^{13}C HMBC.

Figure S9. NMR spectra of $[Ru(dpbpy)_2(L3)](PF_6)_2$ **6,** 400 MHz, CD_2Cl_2: a) ^{1}H-NMR b) ^{13}C-NMR; c) COSY; d) NOESY; e) ^{1}H-^{13}C HSQC; f) ^{1}H-^{13}C HMBC.

Figure S10. ESI-MS spectra of complexes, a) **2**; b) **3**; c) **4**; d) **5**; e) **6**.

Figure S11. Normalized absorption and emission spectra of complexes **2–6** in air-saturated solvents

Figure S12. Frontier molecular orbitals of complexes 2 and 3, including their energies (in eV).

Figure S13. Computed UV-Vis spectra of **2** and **3** at CAMY-B3LYP/TZ2P level in DCM (COSMO). Involved molecular orbitals in the main absorption bands are also included, together with their energies (in eV), oscillator strengths, and their contributions of single orbital transitions.

Figure S14. Computed UV-Vis spectra of **4** and **5** at CAMY-B3LYP/TZ2P level in DCM (COSMO). Involved molecular orbitals in the main absorption bands are also included, together with their energies (in eV), oscillator strengths, and their contributions of single orbital transitions.

Figure S15. Computed UV-Vis spectra of **6** at CAMY-B3LYP/TZ2P level in DCM (COSMO). Involved molecular orbitals in the main absorption bands are also included, together with their energies (in eV), oscillator strengths, and their contributions of single orbital transitions. As a reference, HOMO-LUMO gaps amount to 5.4, 5.2, 4.5, 4.2 and 4.2 eV for **2**, **3**, **4**, **5** and **6**, respectively, computed at the same level of theory, being HOMO destabilized (-7.58, -7.76, -6.96, -6.98, -6.99 eV from **2** to **6**) and LUMO stabilized (-2.19, -2.57, -2.49, -2.78, -2.75 eV from **2** to **6**) from **2** to **6**.

Figure S16. HOMO and LUMO orbitals of compound **2** – **6** together with their energies (in eV), computed at CAMY-B3LYP/TZ2P level in DCM (COSMO). HOMO-LUMO gaps amount to 5.40, 5.20, 4.48, 4.20 and 4.24 eV for **2**, **3**, **4**, **5**, and **6**, respectively, being HOMO destabilized (-7.58, -7.76, -6.96, -6.98, -6.99 eV from **2** to **6**) and LUMO stabilized (-2.19, -2.57, -2.49, -2.78, -2.75 eV from **2** to **6**) from **2** to **6**.

Figure S17. Photodegradation of Ru(II) complexes **2-6** in CH_2Cl_2 under increasing blue-light fluence (0–45 J cm^{-2}). Panels: (A) complex **2**, (B) complex **3**, (C) complex **4**, (D) complex **5**, and (E) complex **6**. (F) Evolution of normalized absorbance versus blue-light fluence for all complexes.

Figure S18. Photodegradation of Ru(II) complexes **2-6** in CH_3OH under increasing blue-light fluence (0–45 J cm^{-2}). Panels: (A) complex **2**, (B) complex **3**, (C) complex **4**, (D) complex **5**, and (E) complex **6**. (F) Evolution of normalized absorbance versus blue-light fluence for all complexes.

Figure S19. Emission spectra of complexes **2–6** in air-saturated dichloromethane (left) and methanol (right). All complexes were excited at 450 nm, where they have the same absorbance value.

Figure S20. Involved molecular orbitals in the main emission bands (in nm) of the computed emission spectra of compounds **2** – **6** at TD-DFT BLYP-D3(BJ)/TZ2P level in DCM (COSMO).

Figure S21. Time-resolved emission kinetic traces of Ru(II) complexes **2–6** in dichloromethane (left) and methanol (right), measured under argon-saturated (red lines) and air-saturated (blue lines) conditions. λ_{exc} = 520 nm; λ_{obs} = 620 nm.

Figure S22 Determination of $^1O_2$ formation quantum yield (Φ_Δ) of complexes **2-6**.

Figure S23. Detection of superoxide radical anion ($O_2^{\bullet -}$) sensitized by complexes **2-6** under blue-light irradiation.

Figure S24. Jablonski diagrams for different excited states in the set of ruthenium complexes.

Figure S25. Normalized absorption spectra of the Ru(II) complexes (**2-6**) in the absence and presence of 50 μg/mL calf thymus DNA (ctDNA).

Figure S26. Photodegradation of the Ru(II) complexes (**2-6**) in the absence and presence of 50 μg/mL ctDNA upon irradiation with blue light (40 J/cm^2).

1. Crystallographic information

[Ru(phen)$_2$(pypz-H)](PF$_6$)$_2$, 2

A total of 327 frames were collected. The total exposure time was 0.91 hours. The frames were integrated with the Bruker SAINT software package using a narrow-frame algorithm. The integration of the data using a monoclinic unit cell yielded a total of 10772 reflections to a maximum θ angle of 21.95° (0.95 Å resolution), of which 1993 were independent (average redundancy 5.405, completeness = 99.7%, R_{int} = 15.58%, R_{sig} = 10.20%) and 1542 (77.37%) were greater than 2σ(F^2). The final cell constants of a = 24.457(6) Å, b = 10.438(3) Å, c = 17.424(5) Å, β = 132.154(5)°, volume = 3297.5(16) Å^3, are based upon the refinement of the XYZ-centroids of 1644 reflections above 20 σ(I) with 6.304° < 2θ < 41.93°. Data were corrected for absorption effects using the Multi-Scan method (SADABS). The ratio of minimum to maximum apparent transmission was 0.195. The calculated minimum and maximum transmission coefficients (based on crystal size) are 0.8490 and 0.9800.
The structure was solved and refined using the Bruker SHELXTL Software Package, using the space group C 1 2/c 1, with Z = 4 for the formula unit, $C_{32}H_{23}F_{12}N_7P_2Ru$. The final anisotropic full-matrix least-squares refinement on F^2 with 244 variables converged at R1 = 9.68%, for the observed data and wR2 = 20.22% for all data. The goodness-of-fit was 1.155. The largest peak in the final difference electron density synthesis was 0.998 e^-/Å^3 and the largest hole was -1.480 e^-/Å^3 with an RMS deviation of 0.158 e^-/Å^3. On the basis of the final model, the calculated density was 1.806 g/cm^3 and F(000), 1784 e^-.

[Ru(phen)$_2$(L1)](PF$_6$)$_2$, 3

A total of 301 frames were collected. The total exposure time was 8.36 hours. The frames were integrated with the Bruker SAINT software package using a narrow-frame algorithm. The integration of the data using a monoclinic unit cell yielded a total of 49148 reflections to a maximum θ angle of 23.47° (0.89 Å resolution), of which 6239 were independent (average redundancy 7.878, completeness = 98.6%, R_{int} = 53.00%, R_{sig} = 27.31%) and 3229 (51.76%) were greater than 2σ(F^2). The final cell constants of a = 38.030(10) Å, b = 12.955(3) Å, c = 17.387(4) Å, β = 91.782(9)°, volume = 8562.(4) Å^3, are based upon the refinement of the XYZ-centroids of 3954 reflections above 20 σ(I) with 5.094° < 2θ < 46.73°. Data were corrected for absorption effects using the Multi-Scan method (SADABS). The ratio of minimum to maximum apparent transmission was 0.489. The calculated minimum and maximum transmission coefficients (based on crystal size) are 0.7450 and 0.9920.
The structure was solved and refined using the Bruker SHELXTL Software Package, using the space group C 1 2/c 1, with Z = 8 for the formula unit, $C_{38}H_{30}Cl_4F_{12}N_6P_2Ru$. The final anisotropic full-matrix least-squares refinement on F^2 with 538 variables converged at R1 = 26.21%, for the observed data and wR2 = 57.96% for all data. The goodness-of-fit was 1.170. The largest peak in the final difference electron density synthesis was 3.476 e^-/Å^3 and the largest hole was -2.785 e^-/Å^3 with an RMS deviation of 0.303 e^-/Å^3. On the basis of the final model, the calculated density was 1.712 g/cm^3 and F(000), 4400 e^-.

[Ru(phen)$_2$(L2)](PF$_6$)$_2$, 4

A total of 1050 frames were collected. The total exposure time was 1.26 hours. The frames were integrated with the Bruker SAINT software package using a narrow-frame algorithm. The integration of the data using a monoclinic unit cell yielded a total of 89536 reflections to a maximum θ angle of 27.54° (0.77 Å resolution), of which 9350 were independent (average redundancy 9.576, completeness = 99.8%, R_{int} = 3.01%, R_{sig} = 1.39%) and 8584 (91.81%) were greater than 2σ(F^2). The final cell constants of a = 9.7432(3) Å, b = 25.0577(7) Å, c = 16.9869(6) Å, β = 101.5870(10)°, volume = 4062.7(2) $Å^3$, are based upon the refinement of the XYZ-centroids of 9564 reflections above 20 σ(I) with 5.159° < 2θ < 55.05°. Data were corrected for absorption effects using the Multi-Scan method (SADABS). The ratio of minimum to maximum apparent transmission was 0.874. The calculated minimum and maximum transmission coefficients (based on crystal size) are 0.8190 and 0.9600.
The structure was solved and refined using the Bruker SHELXTL Software Package, using the space group P 1 21/c 1, with Z = 4 for the formula unit, $C_{39}H_{33}Cl_2F_{12}N_7P_2Ru$. The final anisotropic full-matrix least-squares refinement on F^2 with 570 variables converged at R1 = 3.86%, for the observed data and wR2 = 9.46% for all data. The goodness-of-fit was 1.084. The largest peak in the final difference electron density synthesis was 1.209 $e^-/Å^3$ and the largest hole was -1.182 $e^-/Å^3$ with an RMS deviation of 0.083 $e^-/Å^3$. On the basis of the final model, the calculated density was 1.736 g/cm^3 and F(000), 2128 e^-.

[Ru(phen)$_2$(L3)](PF$_6$)$_2$, 5

A total of 674 frames were collected. The total exposure time was 1.87 hours. The frames were integrated with the Bruker SAINT software package using a narrow-frame algorithm. The integration of the data using a triclinic unit cell yielded a total of 82705 reflections to a maximum θ angle of 27.58° (0.77 Å resolution), of which 9515 were independent (average redundancy 8.692, completeness = 99.7%, R_{int} = 5.90%, R_{sig} = 3.21%) and 8173 (85.90%) were greater than 2σ(F^2). The final cell constants of a = 11.9025(4) Å, b = 13.1126(5) Å, c = 15.6079(5) Å, α = 65.9480(10)°, β = 76.5930(10)°, γ = 68.3350(10)°, volume = 2057.79(13) $Å^3$, are based upon the refinement of the XYZ-centroids of 9836 reflections above 20 σ(I) with 5.889° < 2θ < 54.99°. Data were corrected for absorption effects using the Multi-Scan method (SADABS). The ratio of minimum to maximum apparent transmission was 0.952. The calculated minimum and maximum transmission coefficients (based on crystal size) are 0.9070 and 0.9700.
The structure was solved and refined using the Bruker SHELXTL Software Package, using the space group P -1, with Z = 1 for the formula unit, $C_{85}H_{68}Cl_2F_{24}N_{14}P_4Ru_2$. The final anisotropic full-matrix least-squares refinement on F^2 with 652 variables converged at R1 = 3.72%, for the observed data and wR2 = 8.85% for all data. The goodness-of-fit was 1.058. The largest peak in the final difference electron density synthesis was 1.084 $e^-/Å^3$ and the largest hole was -1.477 $e^-/Å^3$ with an RMS deviation of 0.089 $e^-/Å^3$. On the basis of the final model, the calculated density was 1.726 g/cm^3 and F(000), 1074 e^-.

2. Electrochemical characterization

The electrochemical properties of complexes 2-6 were investigated by cyclic voltammetry experiments in an IJ-Cambria 660C potentiostat using a three-electrode cell. Glassy carbon electrode (3 mm diameter) from BAS was used as working electrode, platinum wire as auxiliary and saturated calomel electrode (SCE) as the reference electrode. The complexes were dissolved in solvents containing the necessary amount of n-$Bu_4N^+PF_6^-$ (TBAH) as supporting electrolyte to yield a 0.1 M ionic strength solution. All redox potentials are referred to SCE. $E_{1/2}$ values were estimated as the average of the oxidative and reductive peak potentials (E_{pa}+E_{pc})/2. The CV profiles of the complexes are shown below:

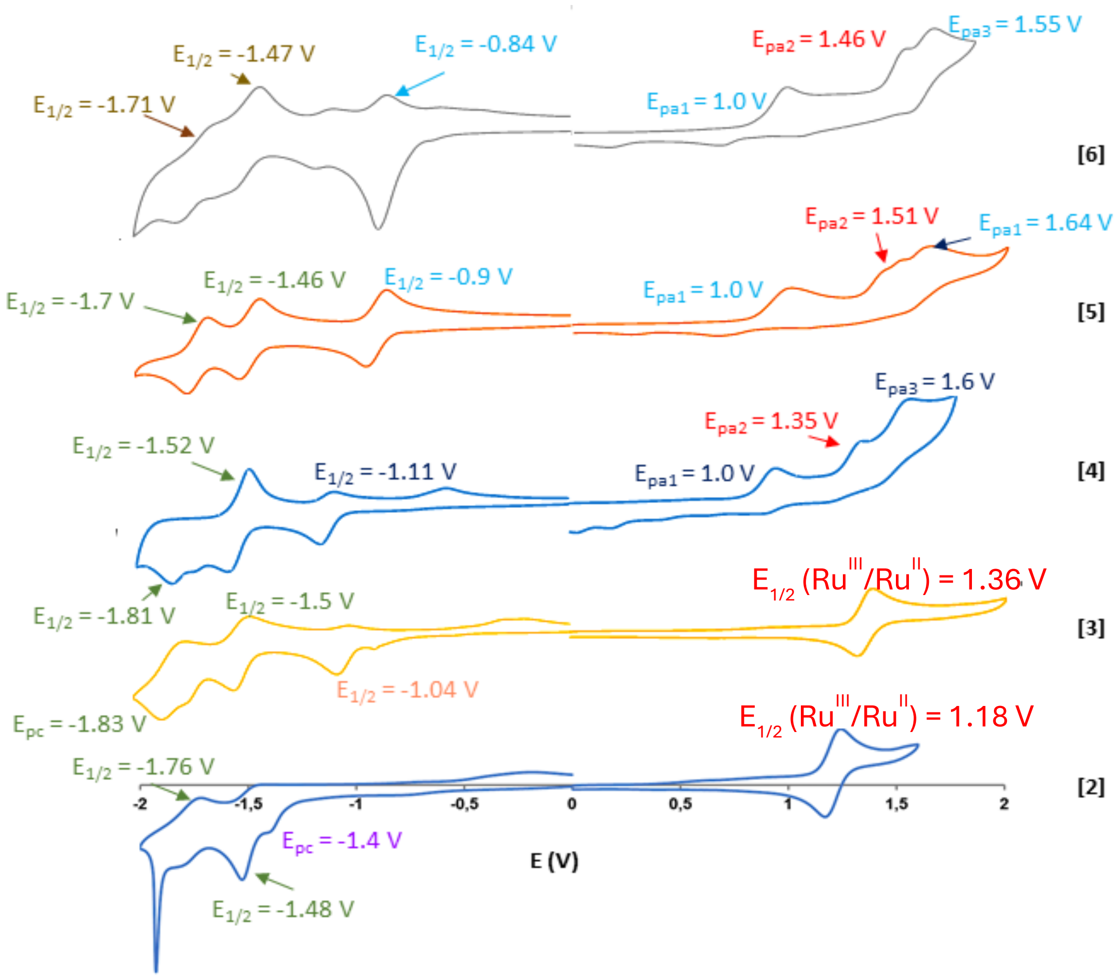

Cyclic voltammograms of Ru(II) complexes 2-6 in CH_3CN (TBAH 0.1 M) *vs* SCE

Compounds **2-5** show successive cathodic waves between -1.45 and -1.9 V, attributed to the *L1* ligands; some of these processes are reversible.[1] In addition, all complexes exhibit further reductive processes: Compound **2** shows an irreversible wave at E_{pc} = -1.4 V, tentatively assigned to the reduction of the *L2* ligand. For compounds **3-5** reversible reduction waves are observed a less negative potentials, $E_{1/2}$ = -1.04

[1] M. N. Ackermann and L. V. Interrante, Ruthenium (II) complexes of modified 1, 10-phenanthrolines. 1. Synthesis and properties of complexes containing dipyridophenazines and a dicyanomethylene-substituted 1, 10-phenanthroline, *Inorg. Chem.*, 1984, **23**, 3904-3911, DOI: 10.1021/ic00192a014

V (**3**), -1.11 V (**4**), -0.9 V (**5**); these signals are assigned to the reductions of the iminopyridine ligands *L3* and *L4* in complexes **3** and **4** and to the iminoquinoline ligand *L5* in complex **5**.[2] For compound **6**, the first reduction peak appears at -0.84 V, along with additional reductive processes at more negative potentials, attributed to the reduction of the *L6* ligands.
Anodic scans reveal that compounds **2** and **3** exhibit one-electron reversible redox waves at $E_{1/2}$ = 1.18 V (**2**) and 1.36 V (**3**) vs SCE, respectively, corresponding to the Ru^{III}/Ru^{II} redox couple. In contrast, the oxidation processes for complexes **4-6** display greater complexity, probably due to the oxidation of the tertiary amine substituents present on the iminopyridine and iminoquinoline ligands. A series of irreversible peaks are observed at E_{pa1} = 1.0 V, E_{pa2} = 1.35 V and E_{pa3} = 1.6 V for (**4**); E_{pa1} = 1.0 V, $E_{pa1'}$ = 1.44 V, E_{pa2} = 1. 51 V, and E_{pa3} = 1.64 V for (**5**) and E_{pa1} = 1.02 V, E_{pa2} = 1. 46 V, and E_{pa3} = 1.55 V for (**6**). These peaks are attributed to ligand-centred oxidation processes involving the iminopyridine *L4* and iminoquinoline *L5* ligands, as well as to metal-centred oxidation. Moreover, weak reduction peaks can be observed on the reverse scan. The processes labelled E_{pa1}, $E_{pa1'}$ and E_{pa3} for every compound, are tentatively assigned to oxidation of the imine and quinoline-based ligands, in agreement with the electrochemical behaviour of tertiary amine-containing ligands.[3] In contrast, the peaks at E_{pa2} = 1.35 V for (**4**), E_{pa2} = 1.51 V for (**5**) and E_{pa2} = 1.46 V for (**6**) are assigned to the oxidation of Ru^{II} to Ru^{III}. A clear trend is observed in the redox potentials across complexes **5** to **6**, with more positive values in the presence of *L1* compared to *L6* ligands, in full agreement with the HOMO's energy level (HOMO is destabilized when we go from **2** to **3** to **4** to **5** to **6**, **(Fig. S15-S14)**. and the blue shift in the lowest energy transitions observed in the absorption spectra. These findings suggest that the *L1* ligand is more electron-withdrawing than *L6*. On the other hand, the lower oxidation potential observed for complex **2**, which contains a pyridine pyrazole ligand, indicates that this ligand has stronger σ-donor properties than the iminopyridine and iminoquinoline ligands. As a result, it increases the electron density at the Ru(II) centre, facilitating its oxidation.

---

[2] F. D. Lesh, M. M. Allard, R. Shanmugam, L. M. Hryhorczuk, J. F. Endicott, H. B. Schlegel and C. N. Verani, Investigation of the Electronic, Photosubstitution, Redox, and Surface Properties of New Ruthenium(II)-Containing Amphiphiles, *Inorg. Chem.*, 2011, **50**, 969-977, DOI: 10.1021/ic1015934

[3] M. N. C. Dunand-Sauthier, A. Deronzier and I. Romero, Electrochemical generation of binuclear complexes $[Mn_2^{III,III}(\mu\text{-}O)(\mu\text{-}OAc)2(bpea)_2]^{2+}$ and $[Mn_2^{IV,IV}(\mu\text{-}O)_2(\mu\text{-}OAc)(bpea)_2]^{3+}$ from the mononuclear $[MnII(bpea)_2]^{2+}$ complex *J. Electroanalytical Chem.*, 1997, **436**, 219-225, DOI: 10.1016/S0022-0728(97)00314-8

3. Supplementary Tables

Table S1. Crystallographic data for the X-ray diffraction of the **2-5** complexes.

	**2**	**3**	**4**	**5**
Empirical formula	$C_{32}H_{23}F_{12}N_7P_2Ru$	$C_{68}H_{55.67}F_{12}N_6O_{1.83}RuP_2$	$C_{39}H_{33}Cl_2F_{12}N_7P_2Ru$	$C_{85}H_{68}Cl_2F_{24}N_{14}P_4Ru_2$
Formula weight	896.58 g/mol	1103.49 g/mol	1061.63	2138.45
Crystal system	Monoclinic	Monoclinic	Monoclinic	Triclinic
Space group	C 1 2/c 1	C 1 2/c 1	P 121/c1	P -1
a[Å]	24.457(6)	38.030(10)	9.7432(3)	11.9025(4)
b[Å]	10.438(3)	12.955(3)	25.0577(7)	13.1126(5)
c[Å]	17.424(5)	17.387(4)	16.9869(6)	15.6079(5)
α[º]	90	90	90	65.9480(10)
β[º]	132.154(5)	91.782(9)°	101.5870(10)	76.5930(10)
γ[º]	90	90	90	68.3350(10)
V [$Å^3$]	3297.5(16)	8562.(4)	4062.7(2)	2057.79(13)
Formula Units/ cell	4	8	4	1
Temp. [K]	100(2)	100(2)	100(2)	100(2)
ρ_{calc}, [Mg/m^{-3}]	1.806	1.712	1.736	1.726
μ[mm^{-1}]	0.679	0.781	0.693	0.622
Final R indices, [I>2σ(I)]	R_1 = 0.0968<br>wR_2 = 0.1869	R_1= 0.2621<br>wR^2= 0.5131	R_1= 0.0386<br>wR^2=0.0916	R_1= 0.0372<br>wR^2=0.0827
R indices [all data]	R_1 = 0.1308<br>wR_2 = 0.2022	R_1= 0.3829<br>wR^2= 0.5796	R_1= 0.0428<br>wR^2= 0.0946	R_1= 0.0488<br>wR^2= 0.0885

Table S2. Selected bond lengths (Å) and angles (°) for of the **2-5** complexes.

**2**		**3**	
Ru(1)-N(13)	2.028(9)	Ru(1)-N(2)	2.04(3)
Ru(1)-N(13#1)	2.028(9)	Ru(1)-N(9)	2.06(3)
Ru(1)-N(16#1)	2.051(10)	Ru(1)-N(16)	2.09(3)
Ru(1)-N(16)	2.051(10)	Ru(1)-N(27)	2.04(3)
Ru(1)-N(2)	2.064(10)	Ru(1)-N(41)	2.07(3)
Ru(1)-N(2#1)	2.064(10)	Ru(1)-N(30)	2.09(3)
N(13)-Ru(1)-N(13#1)	174.1(7)	N(27)-Ru(1)-N(2)	96.7(12)
N(13)-Ru(1)-N(16#1)	89.4(4)	N(27)-Ru(1)-N(9)	83.8(11)
N(13#1)-Ru(1)-N(16#1)	95.2(4)	N(27)-Ru(1)-N(41)	173.2(12)
N(13)-Ru(1)-N(16)	95.2(4)	N(2)-Ru(1)-N(16)	173.5(11)
N(13#1)-Ru(1)-N(16)	89.4(4)	N(9)-Ru(1)-N(16)	96.8(10)
N(16#1)-Ru(1)-N(16)	78.4(6)	N(2)-Ru(1)-N(30)	96.6(11)
N(13)-Ru(1)-N(2)	82.8(4)	N(9)-Ru(1)-N30)	173.3(11)
N(13#1-Ru(1)-N(2)	93.0(4)	N(16)-Ru(1)-N(30)	89.7(11)
N(16#1)-Ru(1)-N(2)	170.2(4)	N(2)-Ru(1)-N(9)	76.9(11)
N(16)-Ru(1)-N(2)	96.5(4)	N(2)-Ru(1)-N(41)	89.5(11)
N(13)-Ru(1)-N(2#1)	93.0(4)	N(9)-Ru(1)-N(41)	100.3(11)
N(13#1)-Ru(1)-N(2#1)	82.8(4)	N(27)-Ru(1)-N(16)	81.0(11)
N(16#1)-Ru(1)-N(2#1)	96.5(4)	N(41)-Ru(1)-N(16)	93.1(11)
N(16)-Ru(1)-N(2#1)	170.2(4)	N(27)-Ru(1)-N(30)	95.6(11)
N(16)-Ru(1)-N(2#1)	89.8(6)	N(41)-Ru(1)-N(30)	81.0(11)

4		5	
Ru(1)-N(9)	2.058(2)	Ru(1)-N(32)	2.056(2)
Ru(1)-N(2)	2.063(2)	Ru(1)-N(37)	2.083(2)
Ru(1)-N(19)	2.063(2)	Ru(1)-N(2)	2.119(2)
Ru(1)-N(44)	2.068(2)	Ru(1)-N(34)	2.059(2)
Ru(1)-N(30)	2.077(2)	Ru(1)-N(48)	2.084(2)
Ru(1)-N(33)	2.066(2)	Ru(1)-N(13)	2.035(2)
N(9)-Ru(1)-N(19)	94.83(8)	N(13)-Ru(1)-N(34)	93.11(8)
N(9)-Ru(1)-N(33)	173.46(8)	N(13)-Ru(1)-N(37)	173.08(8)
N(19)-Ru(1)-N(33)	91.61(8)	N(34)-Ru(1)-N(37)	93.68(8)
N(2)-Ru(1)-N(44)	90.77(8)	N(23)-Ru(1)-N(48)	96.70(8)
N(33)-Ru(1)-N(44)	79.91(8)	N(37)-Ru(1)-N(48)	79.31(8)
N(2)-Ru(1)-N(30)	95.99(8)	N(23)-Ru(1)-N(2)	169.53(8)
N(33)-Ru(1)-N(30)	97.61(8)	N(37)-Ru(1)-N(2)	102.65(8)
N(2)-Ru(1)-N(19)	172.38(8)	N(13)-Ru(1)-N(23)	92.16(8)
N(2)-Ru(1)-N(33)	95.20(8)	N(23)-Ru(1)-N(34)	80.16(9)
N(9)-Ru(1)-N(44)	98.49(8)	N(23)-Ru(1)-N(37)	87.74(8)
N(19)-Ru(1)-N(44)	93.8(8)	N(13)-Ru(1)-N(48)	93.84(8)
N(9)-Ru(1)-N(30)	84.69(8)	N(34)-Ru(1)-N(48)	172.47(8)
N(19)-Ru(1)-N(30)	79.68(8)	N(13)-Ru(1)-N(2)	77.69 (9)
N(44)-Ru(1)-N(30)	173.0(8)	N(34)-Ru(1)-N(2))	97.66(8)
N(9)-Ru(1)-N(2)	78.45(9)	N(48)-Ru(1)-N(2)	86.64(8)

Table S3. List with Cartesian coordinates (in Å), ADF electronic energies (in parentheses, in kcal mol^{-1}), of all complexes optimized at ZORA-BLYP-D3(BJ)/TZ2P level in methanol (COSMO). Charge of all complexes is +2, counterions have not been considered.

[2]$^{2+}$ (-9568.30)

1	Ru	1.17329146	-0.59330499	-0.13743807
2	H	-3.89549503	-1.27994710	-1.34478268
3	H	0.78191224	1.16054778	-4.98717075
4	C	-3.18905539	-1.11485379	-0.53753181
5	H	-4.66759203	-1.09622871	1.03301034
6	H	-1.46923989	-1.08197397	-1.86819722
7	H	1.15349178	-1.54646421	-5.30852594
8	C	4.94948188	-4.30907122	0.42428024
9	C	2.98673268	-2.85907686	0.18806622
10	C	3.52089684	-4.15625511	0.39327277
11	C	5.78209730	-3.23531604	0.25699941
12	C	5.44807726	0.46437528	-0.33750423
13	C	4.04658071	0.55736467	-0.35151598
14	N	3.25574252	-0.51455710	-0.17216522
15	C	3.85343363	-1.74089091	0.01662622
16	C	5.26029095	-1.91309369	0.04565421
17	C	6.06200594	-0.76244743	-0.13756772
18	C	2.60415619	-5.22086007	0.55493575
19	H	6.03286906	1.36689744	-0.48404416
20	H	3.54745386	1.50778056	-0.50194612
21	H	0.51348872	-5.74938271	0.62991521
22	N	1.63292434	-2.61059358	0.13750886
23	H	7.14471756	-0.85021329	-0.12060157
24	C	0.78893870	-3.64377847	0.29946504
25	C	1.24418128	-4.95624855	0.50762559
26	H	6.86089711	-3.36506799	0.28115818
27	H	2.97212935	-6.23049282	0.71469948
28	H	5.35733207	-5.30388116	0.58318850
29	H	-0.27009564	-3.41496980	0.25966122
30	N	1.01017759	-0.37945160	1.92702712
31	C	-0.28521107	-0.47410479	2.38519398
32	C	1.99031715	-0.18123161	2.82498988
33	C	-0.62382889	-0.35514749	3.75675541
34	C	-1.29991280	-0.69335931	1.40830056
35	C	1.73209888	-0.05757548	4.20006653
36	H	2.99921708	-0.11325519	2.43460046
37	C	0.43177590	-0.14034517	4.67304746
38	C	-2.00764392	-0.45841238	4.13010026
39	C	-2.65952196	-0.79377828	1.79718579
40	N	-0.88892457	-0.80431845	0.09877124
41	H	2.56598899	0.10394862	4.87560401
42	H	0.21494714	-0.04319079	5.73313839
43	C	-2.98220462	-0.66829254	3.19194557
44	H	-2.26521695	-0.36600016	5.18190894
45	C	-3.61497244	-1.01235682	0.77764795
46	C	-1.82300591	-1.00548460	-0.84630835
47	H	-4.02518683	-0.74469985	3.48784290
48	N	0.88655836	1.44202536	-0.59424004

49	C	0.77027436	2.44239619	0.30956581
50	C	0.57116136	3.76675707	-0.06609799
51	C	0.48391082	4.08496611	-1.42562261
52	C	0.59737371	3.06388820	-2.36686523
53	C	0.79652098	1.75002165	-1.93462504
54	H	0.84636599	2.15640670	1.35213725
55	H	0.48719038	4.53031188	0.70051418
56	H	0.32975105	5.11105001	-1.74555383
57	H	0.53330244	3.27075853	-3.43033269
58	H	1.36667888	-2.49341732	-2.93983698
59	C	0.93195328	0.60473552	-2.81708273
60	C	0.91229548	0.41244003	-4.21940598
61	C	1.09748103	-0.94888791	-4.41077020
62	N	1.21675673	-1.51926194	-3.17676895
63	N	1.11711164	-0.58998377	-2.19586270

[3]$^{2+}$ (-10434.79)

1	Ru	1.17160340	-0.48611182	-0.31143549
2	H	-3.92880828	-1.07831868	-1.45864241
3	H	3.09562192	-2.09652890	-2.58945988
4	C	-3.20738316	-0.97258977	-0.65485516
5	H	-4.64995923	-1.09071110	0.94412993
6	H	-1.52046701	-0.82591482	-2.01227065
7	N	1.07396367	-0.37930088	-2.37900010
8	C	4.83944951	-4.30421041	0.27897870
9	C	2.91914512	-2.80482325	0.00277034
10	C	3.41847350	-4.12159316	0.16302073
11	C	5.69974787	-3.24143317	0.21263883
12	C	5.47256240	0.47664362	-0.31992571
13	C	4.07641977	0.59903808	-0.41494346
14	N	3.25677395	-0.45828841	-0.29028467
15	C	3.81607968	-1.69972137	-0.07885084
16	C	5.21565308	-1.90210230	0.01862550
17	C	6.04853350	-0.76493505	-0.10046999
18	C	2.47703521	-5.17698855	0.17955825
19	H	6.08271950	1.36804474	-0.42348927
20	H	3.60583157	1.56001730	-0.58809896
21	H	0.38171469	-5.66988990	0.03558431
22	N	1.57900112	-2.52784921	-0.13351717
23	H	7.12656242	-0.87604892	-0.02641132
24	C	0.71170196	-3.55181371	-0.11858884
25	C	1.13027922	-4.88417200	0.03380020
26	H	6.77263411	-3.39364439	0.29407809
27	H	2.81748625	-6.20171658	0.29848242
28	H	5.21921461	-5.31368314	0.41216980
29	H	-0.33676649	-3.30204665	-0.23410774
30	N	1.04090053	-0.40890257	1.77902816
31	C	-0.24422663	-0.54591245	2.24971505
32	C	2.03464957	-0.26486929	2.67179346
33	C	-0.55869659	-0.53378753	3.63256556
34	C	-1.27640130	-0.69436605	1.27750594
35	C	1.80111398	-0.24470425	4.05645376
36	H	3.03636131	-0.15875829	2.27266541
37	C	0.51103383	-0.37785145	4.54403586

38	C	-1.93343562	-0.67864607	4.02355608
39	C	-2.62745381	-0.83517050	1.68495141
40	N	-0.89463794	-0.69734069	-0.04505750
41	H	2.64618315	-0.12336180	4.72638942
42	H	0.31163658	-0.36301838	5.61185992
43	C	-2.92410043	-0.82350888	3.09069098
44	H	-2.17009259	-0.67011895	5.08420333
45	C	-3.60416048	-0.97838299	0.67251417
46	C	-1.84914554	-0.82853122	-0.98092785
47	H	-3.96083775	-0.93213209	3.39795453
48	N	0.90582030	1.55193920	-0.62625877
49	C	0.84669489	2.51845982	0.31559697
50	C	0.63026003	3.85664646	-0.00741277
51	C	0.46404401	4.22584680	-1.34656262
52	C	0.52122020	3.23790411	-2.32751563
53	C	0.74219994	1.90920457	-1.94875816
54	H	0.97848334	2.19861747	1.34275493
55	H	0.59276610	4.59300010	0.78889118
56	H	0.29358010	5.26292297	-1.61826835
57	H	0.39813165	3.47574649	-3.37955789
58	H	0.73851874	0.96966716	-3.94746297
59	C	0.83997121	0.81004521	-2.87660629
60	C	1.13109351	-1.51261166	-3.25457525
61	C	0.05589047	-1.80447207	-4.10699863
62	C	0.10107968	-2.94725345	-4.91146564
63	C	1.21691969	-3.79006359	-4.87594400
64	C	2.29703901	-3.48152099	-4.03815786
65	C	2.25545215	-2.34945796	-3.22439403
66	H	-0.81232893	-1.15177004	-4.12935154
67	H	-0.73965278	-3.17758531	-5.56076362
68	H	1.24749514	-4.68044937	-5.49853885
69	H	3.17446063	-4.12280932	-4.01886281

[4]$^{2+}$ (-11426.39)

1	Ru	1.19613509	-0.49395725	-0.32233754
2	H	-3.89730174	-1.12311932	-1.48423584
3	H	2.80598993	-2.36892985	-2.50033397
4	C	-3.17921602	-1.00280425	-0.67942332
5	H	-4.63091464	-1.08839609	0.91350937
6	H	-1.48410552	-0.88665681	-2.03136087
7	N	1.04694541	-0.36068217	-2.40469850
8	C	4.88603726	-4.27925136	0.32184795
9	C	2.95662439	-2.79815208	0.01427109
10	C	3.46405943	-4.10849615	0.20142787
11	C	5.73936652	-3.21235924	0.23194860
12	C	5.48755780	0.49279224	-0.37224783
13	C	4.09054663	0.60522624	-0.46439530
14	N	3.27629878	-0.45425337	-0.31855946
15	C	3.84587187	-1.68896081	-0.09015607
16	C	5.24699741	-1.88048274	0.00979152
17	C	6.07271873	-0.74082876	-0.13183559
18	C	2.52912687	-5.16946360	0.23769826
19	H	6.09201852	1.38601443	-0.49323404
20	H	3.61326231	1.56052065	-0.65035721

21	H	0.43723793	-5.67810407	0.09923123
22	N	1.61438564	-2.53214824	-0.13239698
23	H	7.15159728	-0.84336183	-0.05715028
24	C	0.75436958	-3.56154236	-0.09885850
25	C	1.18084769	-4.88774843	0.08301219
26	H	6.81323376	-3.35631535	0.31611943
27	H	2.87538572	-6.18947223	0.37856861
28	H	5.27262965	-5.28305973	0.47684303
29	H	-0.29522065	-3.32138756	-0.22459503
30	N	1.06044630	-0.42828192	1.76568643
31	C	-0.22840271	-0.54690081	2.23265105
32	C	2.05285304	-0.29352237	2.66169282
33	C	-0.54760776	-0.52303524	3.61421930
34	C	-1.25786357	-0.69618882	1.25749979
35	C	1.81428841	-0.26120460	4.04530420
36	H	3.05730533	-0.20260297	2.26526239
37	C	0.52047468	-0.37373743	4.52888572
38	C	-1.92524892	-0.65259246	4.00081630
39	C	-2.61110858	-0.82798436	1.66084690
40	N	-0.86973669	-0.71613399	-0.06318338
41	H	2.65802517	-0.14711955	4.71826241
42	H	0.31761001	-0.34931464	5.59587547
43	C	-2.91325038	-0.79935477	3.06523998
44	H	-2.16646032	-0.63282752	5.06032400
45	C	-3.58325160	-0.98362803	0.64586653
46	C	-1.81910296	-0.86643726	-1.00172199
47	H	-3.95172649	-0.89863662	3.36995199
48	N	0.92220432	1.54826963	-0.59499413
49	C	0.89225149	2.49879714	0.36552281
50	C	0.66491649	3.84148444	0.07562503
51	C	0.45416676	4.23248141	-1.25305713
52	C	0.48027328	3.26344794	-2.25145107
53	C	0.71600924	1.92458595	-1.90618005
54	H	1.05856777	2.16092636	1.38189828
55	H	0.65320727	4.56398900	0.88532840
56	H	0.27359199	5.27401913	-1.50107204
57	H	0.32164525	3.52042996	-3.29438337
58	H	0.64229611	1.03940041	-3.91656694
59	C	0.78486736	0.84928207	-2.85566400
60	C	1.01852066	-1.46223759	-3.29654617
61	C	0.01398176	-1.58625132	-4.27772969
62	C	-0.03091818	-2.68615504	-5.12209881
63	C	0.95021209	-3.71650041	-5.03938183
64	C	1.97766385	-3.56185186	-4.06279378
65	C	1.99580884	-2.47027475	-3.21083307
66	H	-0.76175583	-0.82867184	-4.35527191
67	H	-0.84126825	-2.75784089	-5.83804585
68	N	0.90477180	-4.81356526	-5.85894589
69	H	2.76788155	-4.29854069	-3.97862545
70	H	-1.13290580	-4.93147163	-6.45438335
71	H	2.92687856	-5.46887172	-6.00348870
72	C	1.92923442	-5.85864892	-5.75859304
73	H	1.96061314	-6.28347276	-4.74706729
74	H	1.68738393	-6.65685648	-6.46041539

75	C	-0.12758782	-4.92398966	-6.89538839
76	H	-0.06631854	-4.09127995	-7.60897270
77	H	0.01767953	-5.85805896	-7.43796493

[5]$^{2+}$ (-12386.13)

1	Ru	1.21438271	-0.44226076	-0.28438841
2	H	-3.91710674	-0.61885961	-1.47966179
3	H	3.06872740	-2.07727181	-2.52683472
4	C	-3.19735055	-0.60235711	-0.66766622
5	H	-4.66083694	-0.61879684	0.91650071
6	H	-1.49507237	-0.59486154	-2.00799797
7	N	1.04284857	-0.35315879	-2.35073632
8	C	4.78506369	-4.35119480	0.21616344
9	C	2.90361159	-2.80112810	-0.03199191
10	C	3.37038603	-4.13421461	0.08102874
11	C	5.66852198	-3.30499574	0.20665966
12	C	5.52394056	0.43479046	-0.20025687
13	C	4.13281348	0.58921278	-0.31678180
14	N	3.29040306	-0.45578006	-0.24213863
15	C	3.82416163	-1.71418093	-0.06150850
16	C	5.21717355	-1.94874027	0.05283561
17	C	6.07326197	-0.82467702	-0.01222757
18	C	2.40503219	-5.16653156	0.02983763
19	H	6.15355198	1.31642800	-0.26387915
20	H	3.68091288	1.56257990	-0.47011665
21	H	0.30328684	-5.60421051	-0.18232001
22	N	1.57293884	-2.48768820	-0.18182459
23	H	7.14761402	-0.95838640	0.07606979
24	C	0.68177807	-3.48928726	-0.23179918
25	C	1.06836957	-4.83616111	-0.13247218
26	H	6.73605283	-3.48404185	0.30242234
27	H	2.71875642	-6.20347563	0.11024762
28	H	5.14174614	-5.37274928	0.31706096
29	H	-0.35757058	-3.20812795	-0.35777632
30	N	1.06000927	-0.56269237	1.81557778
31	C	-0.23639350	-0.58732867	2.26913877
32	C	2.05032570	-0.66644439	2.71913854
33	C	-0.56438738	-0.65146624	3.64835754
34	C	-1.26681644	-0.56974185	1.28437088
35	C	1.80536336	-0.74522080	4.09871635
36	H	3.06351324	-0.65869219	2.33508529
37	C	0.50314553	-0.72245822	4.57222741
38	C	-1.95067065	-0.65532443	4.02441655
39	C	-2.62957631	-0.59547955	1.67740492
40	N	-0.87250853	-0.56243588	-0.03476020
41	H	2.64996703	-0.81479226	4.77651305
42	H	0.29331530	-0.76873245	5.63705234
43	C	-2.93987910	-0.62538560	3.07983849
44	H	-2.19692066	-0.69136935	5.08225895
45	C	-3.60647358	-0.60335273	0.65542008
46	C	-1.82812004	-0.58466241	-0.97883524
47	H	-3.98585865	-0.63508576	3.37455350
48	N	0.91968132	1.64900710	-0.61884621
49	C	0.95767345	2.70618209	0.27300766

50	C	0.45602491	4.00504972	-0.09806629
51	C	-0.02484341	4.20160410	-1.41924100
52	C	0.02780661	3.16544485	-2.31667016
53	C	0.50495792	1.89823470	-1.89615096
54	H	3.36494025	-5.33237894	-5.90321675
55	H	-0.75058311	-5.35047445	-6.04157754
56	H	-0.41014797	5.17585691	-1.70803319
57	H	-0.29919744	3.28355004	-3.34504823
58	H	0.36703983	0.93561380	-3.86410925
59	C	0.60663723	0.80030227	-2.81214477
60	C	1.11411780	-1.47790311	-3.21016330
61	C	0.07292358	-1.78547457	-4.10818924
62	C	0.12495029	-2.92229084	-4.90117806
63	C	1.24323628	-3.80490373	-4.84898282
64	C	2.30878636	-3.45617694	-3.96675445
65	C	2.23169768	-2.33076964	-3.16501023
66	H	-0.80518386	-1.14722155	-4.15968453
67	H	-0.71424570	-3.13953811	-5.55129066
68	N	1.29016138	-4.94461491	-5.60764498
69	H	3.20142067	-4.06826770	-3.91726569
70	C	1.00163665	4.85422655	2.11865257
71	C	0.47792526	5.05485418	0.85642669
72	H	1.94990385	1.59155629	1.82812317
73	H	1.98243420	3.44396768	3.44517806
74	C	1.51705764	2.54269908	1.56360694
75	C	1.53982932	3.59129527	2.46337675
76	H	0.08371368	6.02563960	0.56534860
77	H	1.01701130	5.66508990	2.84192806
78	C	0.21352582	-5.24900841	-6.55652002
79	H	0.11878818	-4.46481149	-7.31980450
80	H	0.43904452	-6.19223132	-7.05365182
81	C	2.45533507	-5.83325487	-5.54336766
82	H	2.63194466	-6.17726696	-4.51650972
83	H	2.26844421	-6.70485752	-6.17044105

[6]$^{2+}$ (-17666.98)

1	Ru	1.22521067	-0.48509825	-0.23371875
2	H	-3.82997851	-0.62670228	-1.54512604
3	H	3.24879753	-1.98948992	-2.55458439
4	C	-3.14738208	-0.58787119	-0.70319279
5	H	-4.88898681	-1.64018868	2.77581620
6	H	-1.40965215	-0.64369321	-1.96317318
7	N	1.09737703	-0.43715654	-2.30133685
8	C	1.39563481	2.54088432	1.55990311
9	C	2.93003515	-2.87217869	-0.06701538
10	C	3.32138837	-4.20574777	-0.17034577
11	C	1.37329683	3.60826327	2.43707066
12	C	5.46144969	0.50469163	0.01775420
13	C	4.09174158	0.58431720	-0.17456066
14	N	3.29122512	-0.50838493	-0.16479559
15	C	3.86822002	-1.74381445	0.01687824
16	C	5.23990941	-1.87326144	0.22507146
17	C	6.07610222	-0.74514848	0.23444801
18	C	2.37787484	-5.21152582	-0.42835124

19	H	6.04700488	1.41673738	-0.02041712
20	H	3.59760372	1.53381500	-0.34353791
21	H	0.24508187	-5.53249219	-0.72252463
22	N	1.61166914	-2.51145762	-0.17493890
23	H	7.72457414	1.03948025	1.46555762
24	C	0.68726611	-3.47937107	-0.36925280
25	C	1.02899667	-4.81372305	-0.51067514
26	H	-0.03313384	5.98735895	0.43388189
27	H	4.69840598	-6.09916166	-1.54130183
28	H	0.81819255	5.68440517	2.75030911
29	H	-0.34155822	-3.14966689	-0.44881436
30	N	1.04318847	-0.57997709	1.85668403
31	C	-0.23448176	-0.59085826	2.35115922
32	C	2.06916221	-0.68340088	2.73689071
33	C	-0.47778096	-0.65487439	3.72316649
34	C	-1.29432871	-0.56765571	1.33423897
35	C	1.88127984	-0.75446561	4.10663870
36	H	3.06729074	-0.67825721	2.31816939
37	C	0.57913704	-0.72721712	4.64267949
38	H	1.82178414	1.59810798	1.86339370
39	C	-2.65514315	-0.54057322	1.63618864
40	N	-0.85467647	-0.59138161	0.03404742
41	H	2.75299303	-0.80305543	4.74999304
42	H	2.06244539	-2.01006209	6.53472281
43	H	1.77628457	3.48560121	3.43911879
44	H	5.65663135	-2.85669167	0.40792617
45	C	-3.62326804	-0.54809345	0.62058861
46	C	-1.78425346	-0.60557268	-0.95013372
47	H	4.37115910	-4.45863653	-0.08222427
48	N	0.89799475	1.59793344	-0.62651611
49	C	0.88787596	2.67290314	0.24406857
50	C	0.38461911	3.95750116	-0.17243356
51	C	-0.05456965	4.11899628	-1.51250568
52	C	0.03561930	3.06270427	-2.38365580
53	C	0.51587234	1.81242775	-1.92039045
54	H	3.46982952	-5.79234337	-5.12276730
55	H	-0.61565040	-6.06745656	-4.95026666
56	H	-0.44080771	5.08199102	-1.83571536
57	H	-0.26298495	3.15327835	-3.42341492
58	H	0.42952308	0.78880007	-3.86731217
59	C	0.65398095	0.69135482	-2.80745045
60	C	1.20453568	-1.60181698	-3.11130773
61	C	0.11723428	-2.06996548	-3.86970407
62	C	0.17473772	-3.29888275	-4.51602916
63	C	1.34243740	-4.11046504	-4.44857694
64	C	2.45981704	-3.58586489	-3.73624697
65	C	2.38193022	-2.36825651	-3.08269114
66	H	-0.80260265	-1.49309754	-3.91436531
67	H	-0.70345644	-3.64431899	-5.04832106
68	N	1.39314590	-5.35020268	-5.03895488
69	H	3.38813239	-4.14214231	-3.68776570
70	C	0.83821420	4.85854417	2.04432261
71	C	0.36033837	5.02736887	0.75960175
72	H	-1.50001995	-0.67811667	4.07991201

73	H	-2.96637750	-0.49070528	2.67229964
74	C	0.33290883	-0.78068334	6.09969422
75	C	-0.78168775	-0.12921403	6.66474615
76	C	-1.01136045	-0.17672804	8.04109532
77	C	-0.13585895	-0.88054776	8.87713881
78	C	0.97388438	-1.53374613	8.32697563
79	C	1.20924188	-1.48202820	6.95165118
80	H	-1.45591597	0.43975162	6.03015267
81	H	-1.86989116	0.34119185	8.46175089
82	H	-0.31649463	-0.91906790	9.94867638
83	H	1.65377341	-2.08890137	8.96875603
84	C	2.78631237	-6.61354755	-0.66065153
85	C	1.92794886	-7.68140570	-0.33193355
86	C	2.30625860	-9.00149637	-0.58420692
87	C	3.54526760	-9.27947312	-1.17459124
88	C	4.40635483	-8.22700439	-1.50736377
89	C	4.03207962	-6.90677883	-1.25124643
90	H	0.97126120	-7.48061986	0.14225879
91	H	1.63552414	-9.81403644	-0.31564346
92	H	3.83655468	-10.30770035	-1.37471972
93	H	5.36551579	-8.43312427	-1.97593980
94	C	7.53236555	-0.86829121	0.45810261
95	C	8.23280316	-2.01301942	0.02773174
96	C	9.60824734	-2.12655960	0.23845647
97	C	10.30809391	-1.10228025	0.88807308
98	C	9.62277445	0.03921779	1.32181868
99	C	8.24839951	0.15799997	1.10605657
100	H	7.70646997	-2.80475656	-0.49850627
101	H	10.13494712	-3.01149686	-0.11047481
102	H	11.37888860	-1.19249377	1.05401307
103	H	10.15787152	0.83532010	1.83387241
104	C	-5.06869983	-0.51953338	0.93025853
105	C	-5.97632352	0.11316817	0.05758902
106	C	-7.34099300	0.14460799	0.35143964
107	C	-7.82469913	-0.46113203	1.51765716
108	C	-6.93287618	-1.09664047	2.39034800
109	C	-5.56698735	-1.12354203	2.10186648
110	H	-5.61075656	0.60465386	-0.83990322
111	H	-8.02616624	0.64719450	-0.32698083
112	H	-8.88792142	-0.43854862	1.74428671
113	H	-7.30181457	-1.57756777	3.29306733
114	C	0.19682477	-5.91017934	-5.67559692
115	H	-0.16993521	-5.24764758	-6.46800273
116	H	0.45381098	-6.86962345	-6.12494180
117	C	2.52994265	-6.24089454	-4.77930668
118	H	2.62753241	-6.47338958	-3.70932037
119	H	2.37911799	-7.17182275	-5.32590936

Table S4. Concentration of Ru(II) in *S. aureus* and *E. coli* bacteria. A preinoculum was prepared by inoculating a single bacterial colony into 5 mL of TSB medium. The following day, 280 µL of this preinoculum was added to 10 mL of fresh TSB in each of six 50 mL tubes, which were then incubated at 37ºC with shaking until the cultures reached an OD_{600} of approximately 0.3, typically within 60 to 90 minutes. Once the desired optical density was achieved, the cultures were washed three times with PBS by centrifugation at 6000g for 10 minutes each. The resulting pellets were resuspended in approximately one-third of the original volume to concentrate the samples and reach an OD_{600} of around 0.9 to 1 (~10^8 CFU·mL^{-1}). All tubes were then pooled to create a homogeneous bacterial suspension, which was subsequently divided into 1 mL aliquots in individual Eppendorf vials. To each vial, compounds were added to reach a final concentration of 10 µM using pre-prepared stock solutions in DMSO at 1 mg/mL. The samples were incubated in the dark for 60 minutes. After incubation, 100 µL from each sample was transferred to a 96-well plate for later dilutions, plating, and colony counting. The remaining 900 µL in each vial was centrifuged at 8000g for 10 minutes and washed with PBS. Three cycles of centrifugation and washing were carried out. Following the final centrifugation, supernatants and pellets were mineralized with 100 µL of concentrated nitric acid, diluted with milliQ water to 5 mL, and the Ru concentration in the solutions was determined by ICP-MS. Appropriate controls were included.

Sample ID	Bacteria	Complex	Sample	[Ru] (µg/L)	m(Ru) (µg)
1A	*S. aureus*	blank	pellet	<0.5	<0.0025
1B	*S. aureus*	blank	pellet	<0.5	<0.0025
1C	*S. aureus*	blank	supernatant	<0.5	<0.0025
1D	*S. aureus*	blank	supernatant	<0.5	<0.0025
2A	*S. aureus*	**2**	pellet	2.05	0.0102
2B	*S. aureus*	**2**	pellet	2.07	0.0103
2C	*S. aureus*	**2**	supernatant	<0.5	<0.0025
2D	*S. aureus*	**2**	supernatant	<0.5	<0.0025
3A	*S. aureus*	**3**	pellet	1.41	0.0070
3B	*S. aureus*	**3**	pellet	1.76	0.0088
3C	*S. aureus*	**3**	supernatant	<0.5	<0.0025
3D	*S. aureus*	**3**	supernatant	<0.5	<0.0025
4A	*S. aureus*	**4**	pellet	0.50	0.0025
4B	*S. aureus*	**4**	pellet	0.56	0.0028
4C	*S. aureus*	**4**	supernatant	<0.5	<0.0025
4D	*S. aureus*	**4**	supernatant	<0.5	<0.0025
5A	*S. aureus*	**5**	pellet	1.74	0.0087
5B	*S. aureus*	**5**	pellet	1.44	0.0072
5C	*S. aureus*	**5**	supernatant	<0.5	<0.0025
5D	*S. aureus*	**5**	supernatant	<0.5	<0.0025
6A	*S. aureus*	**6**	pellet	106.70	0.5335
6B	*S. aureus*	**6**	pellet	103.40	0.5170
6C	*S. aureus*	**6**	supernatant	<0.5	<0.0025
6D	*S. aureus*	**6**	supernatant	<0.5	<0.0025

Sample ID	Bacteria	Complex	Sample	[Ru] (μg/L)	m(Ru) (μg)
8A	*E. coli*	blank	pellet	<0.5	<0.0025
8B	*E. coli*	blank	pellet	<0.5	<0.0025
8C	*E. coli*	blank	supernatant	<0.5	<0.0025
8D	*E. coli*	blank	supernatant	<0.5	<0.0025
9A	*E. coli*	**2**	pellet	1.43	0.0071
9B	*E. coli*	**2**	pellet	1.36	0.0068
9C	*E. coli*	**2**	supernatant	<0.5	<0.0025
9D	*E. coli*	**2**	supernatant	<0.5	<0.0025
10A	*E. coli*	**3**	pellet	<0.5	<0.0025
10B	*E. coli*	**3**	pellet	<0.5	<0.0025
10C	*E. coli*	**3**	supernatant	<0.5	<0.0025
10D	*E. coli*	**3**	supernatant	<0.5	<0.0025
11A	*E. coli*	**4**	pellet	<0.5	<0.0025
11B	*E. coli*	**4**	pellet	<0.5	<0.0025
11C	*E. coli*	**4**	supernatant	<0.5	<0.0025
11D	*E. coli*	**4**	supernatant	<0.5	<0.0025
12A	*E. coli*	**5**	pellet	0.96	0.0048
12B	*E. coli*	**5**	pellet	0.95	0.0047
12C	*E. coli*	**5**	supernatant	<0.5	<0.0025
12D	*E. coli*	**5**	supernatant	<0.5	<0.0025
13A	*E. coli*	**6**	pellet	82.97	0.4149
13B	*E. coli*	**6**	pellet	81.92	0.4096
13C	*E. coli*	**6**	supernatant	<0.5	<0.0025
13D	*E. coli*	**6**	supernatant	<0.5	<0.0025
15A	HNO3	blank	--	<0.5	<0.0025
15B	HNO3	blank	--	<0.5	<0.0025

3. Supplementary Figures

Figure S1. Intermolecular hydrogen bonds observed in compound **5**.

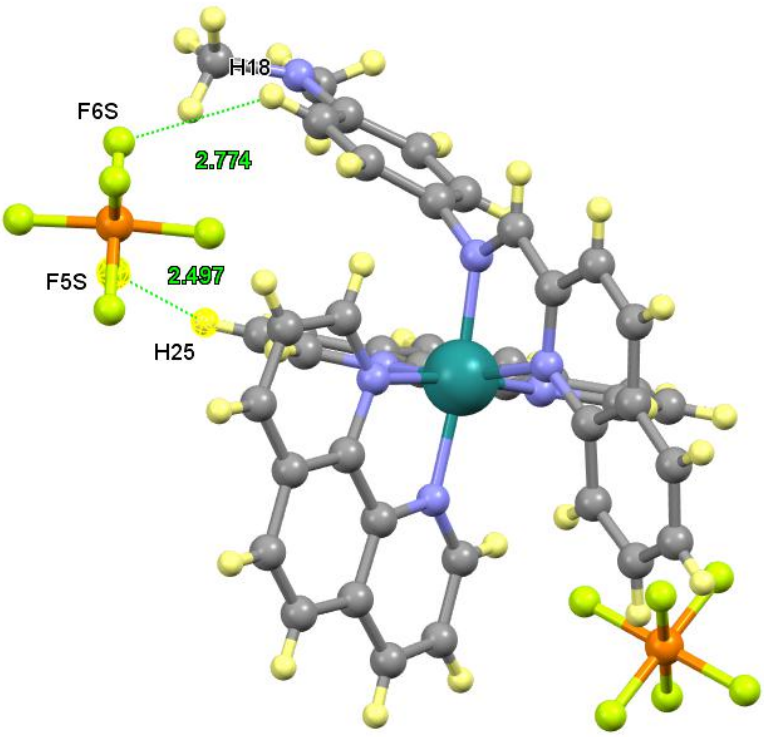

Figure S2. Packing of *rac*-**2** along a) *b* and b) *c* axis; packing of *rac*-**3** along c) *b* and d) *c* axis; packing of *rac*-**4** along e) *a* axis; packing of *rac*-**5** along f) *b* axis.

a b

c d

e f

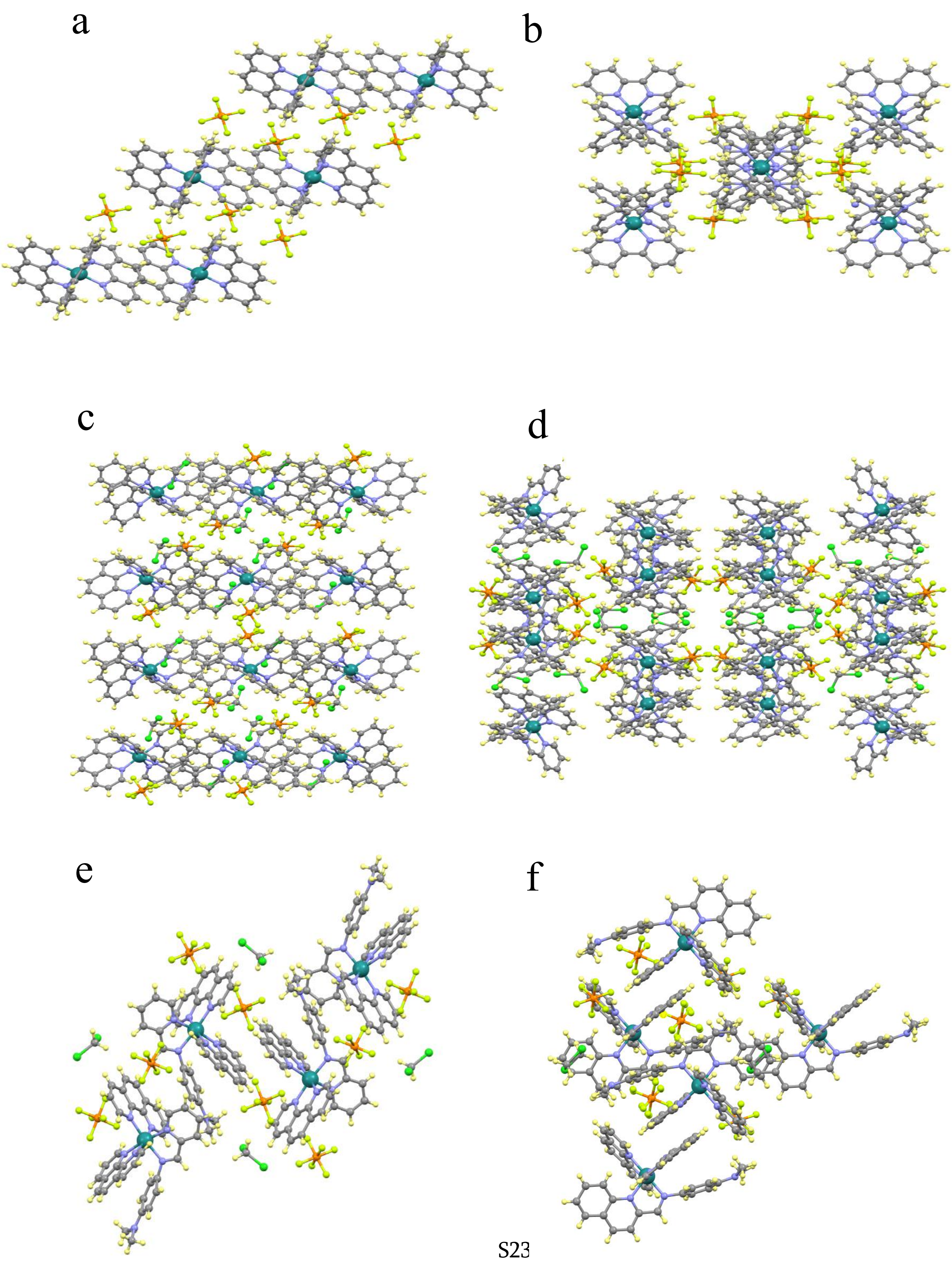

Figure S3. Geometries of the ruthenium clusters under analysis (without counterions), optimized at ZORA-BLYP-D3(BJ)/TZ2P level in methanol (COSMO). Bond lengths Ru-N are also enclosed (in Å).

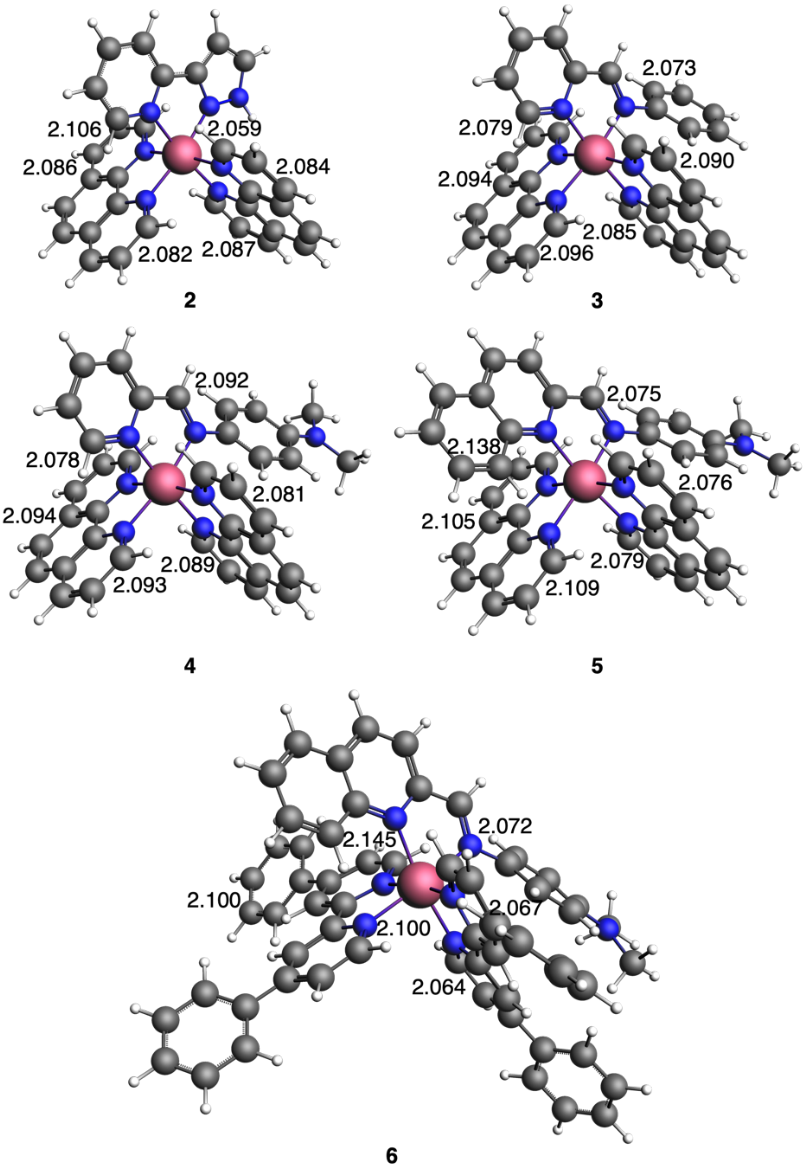

Figure S4. Spectra of Ru(II) complexes **2-6**.

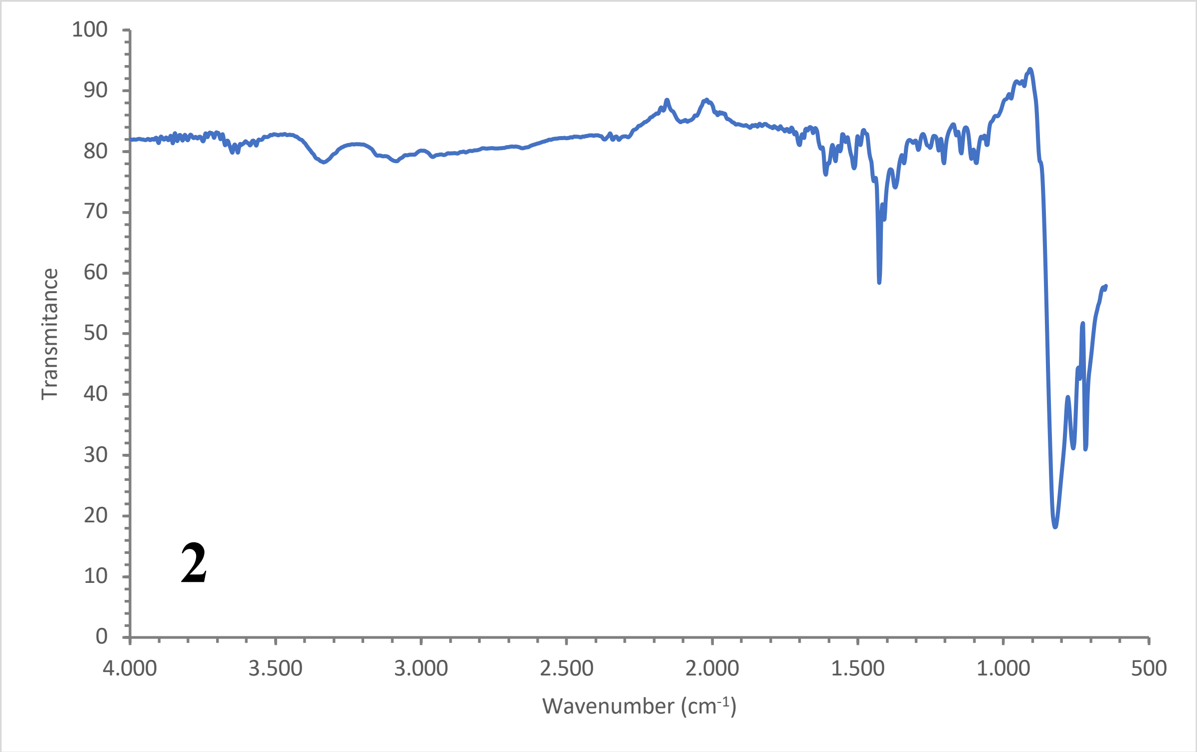

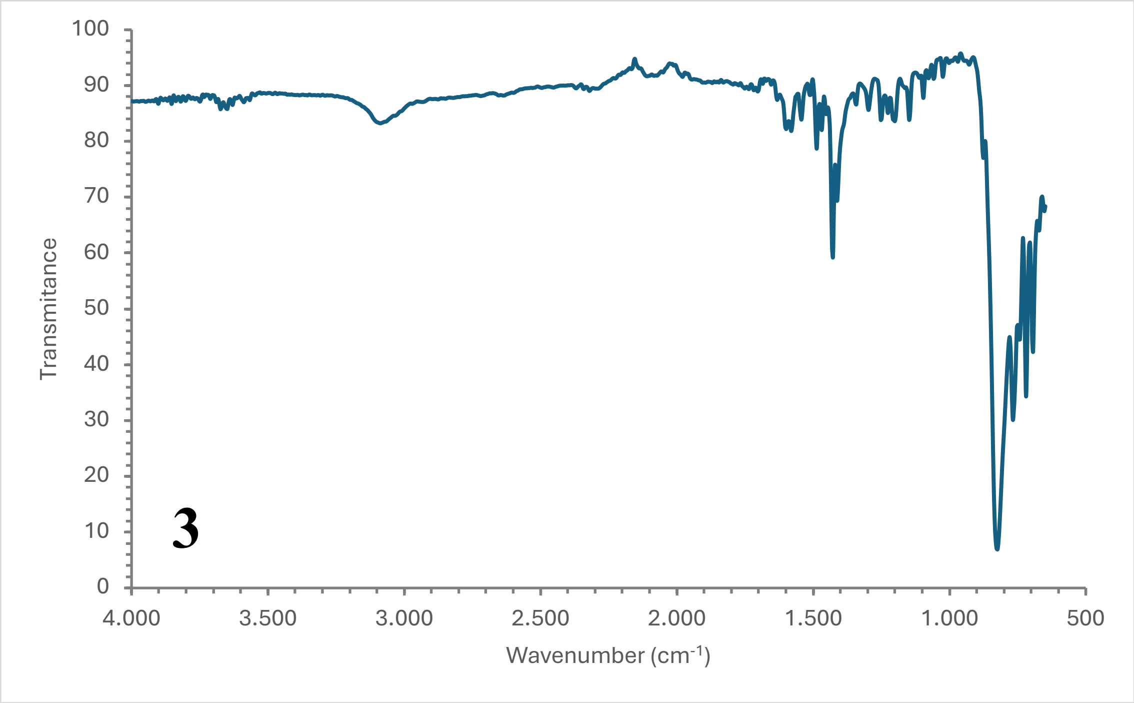

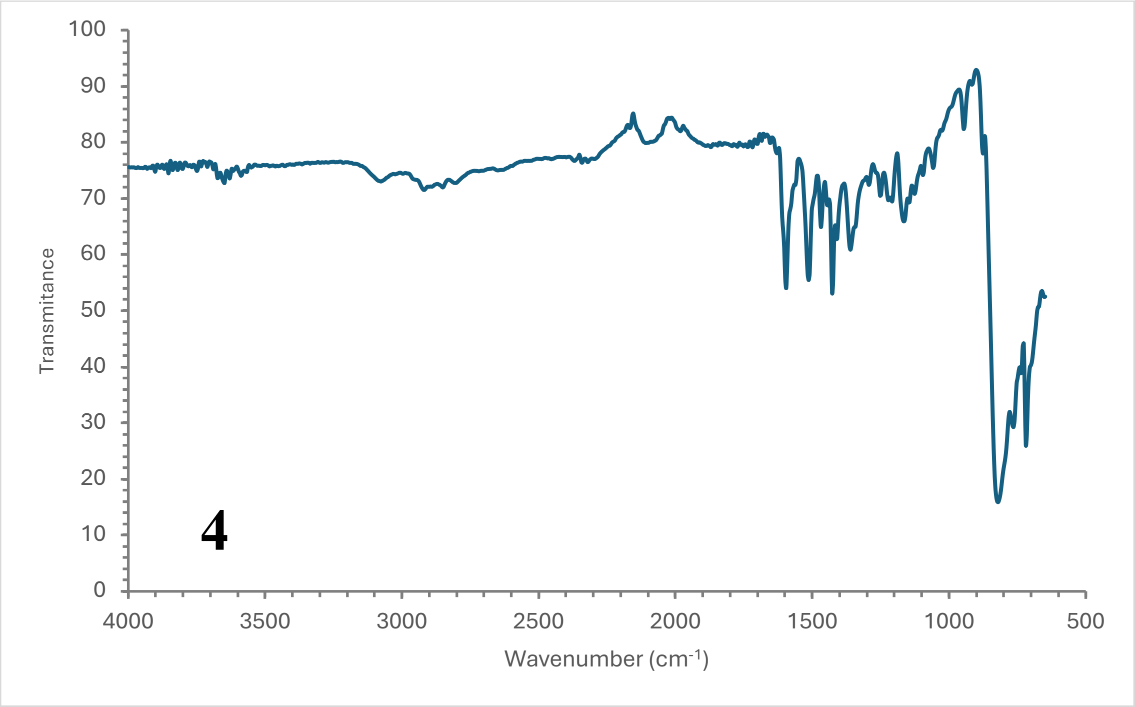
100
90
80
70
60
50
40
30
20
10
0
Transmitance
4
4000
3500
3000
2500
2000
1500
1000
500
Wavenumber (cm-1)

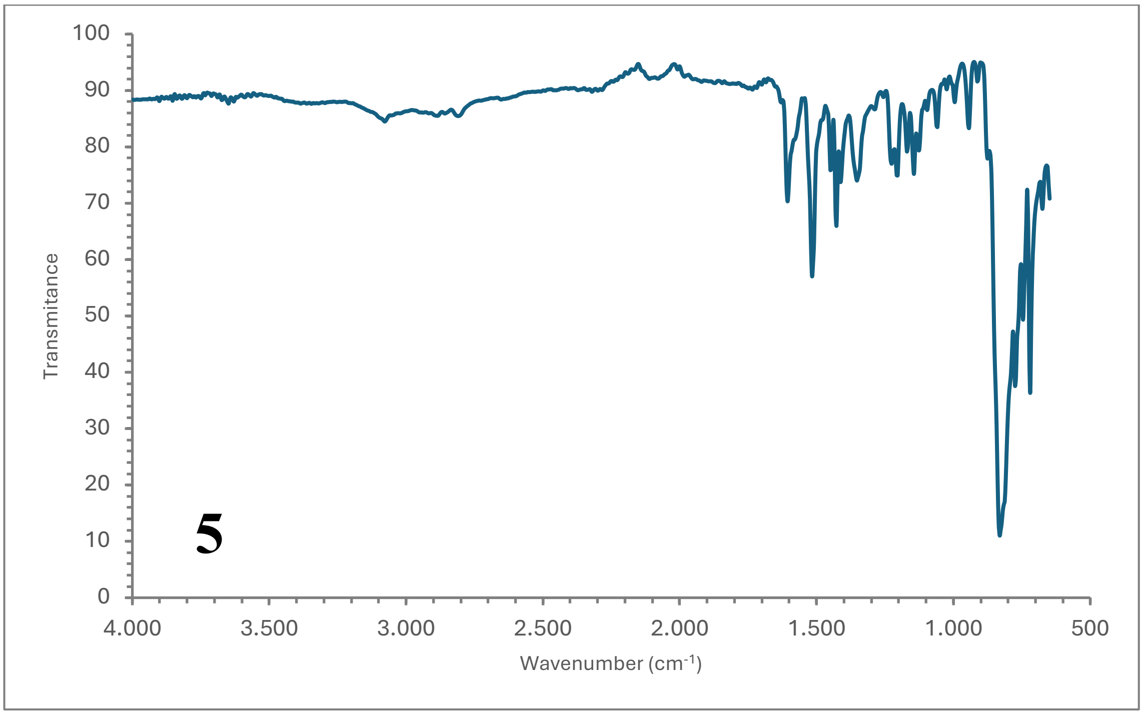
100
90
80
70
60
50
40
30
20
10
0
Transmitance
5
4.000
3.500
3.000
2.500
2.000
1.500
1.000
500
Wavenumber (cm-1)

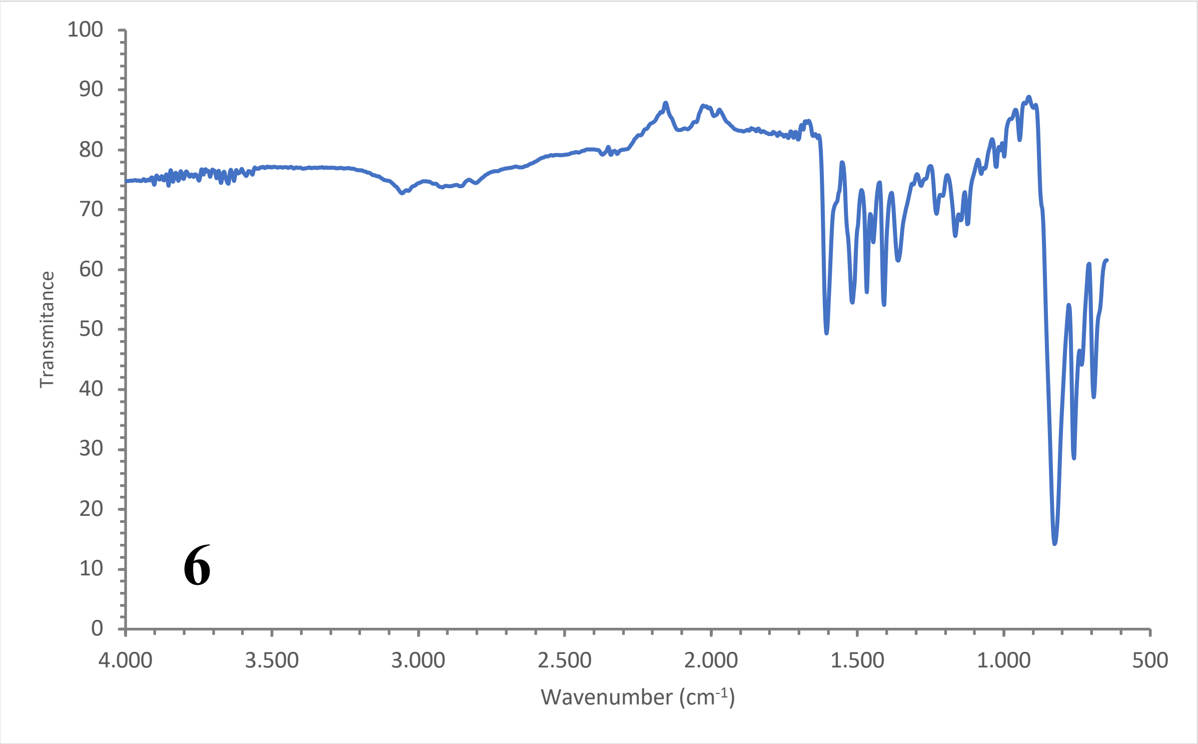

100
90
80
70
60
50
40
30
20
10
0
Transmitance
6
4.000
3.500
3.000
2.500
2.000
1.500
1.000
500
Wavenumber (cm-1)

Figure S5. NMR spectra of Ru(II) complex **2**, 400 MHz, CD_2Cl_2: a) ^{1}H-NMR; b) COSY; c) NOESY; d) ^{1}H-^{13}C HSQC; e) ^{1}H-^{13}C HMBC.

a)

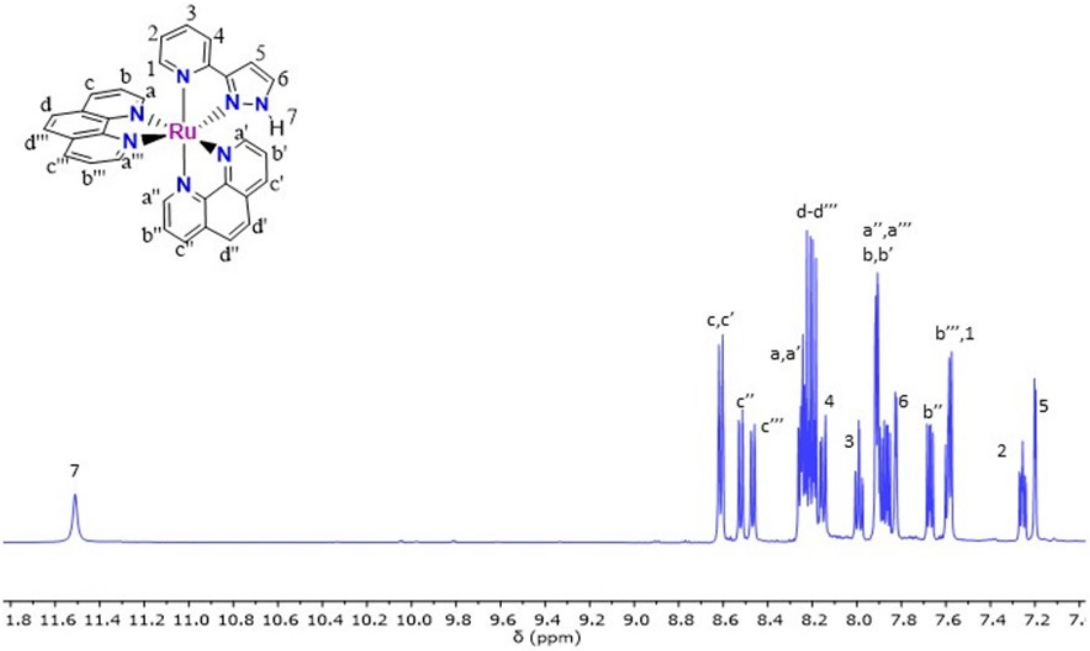

b)

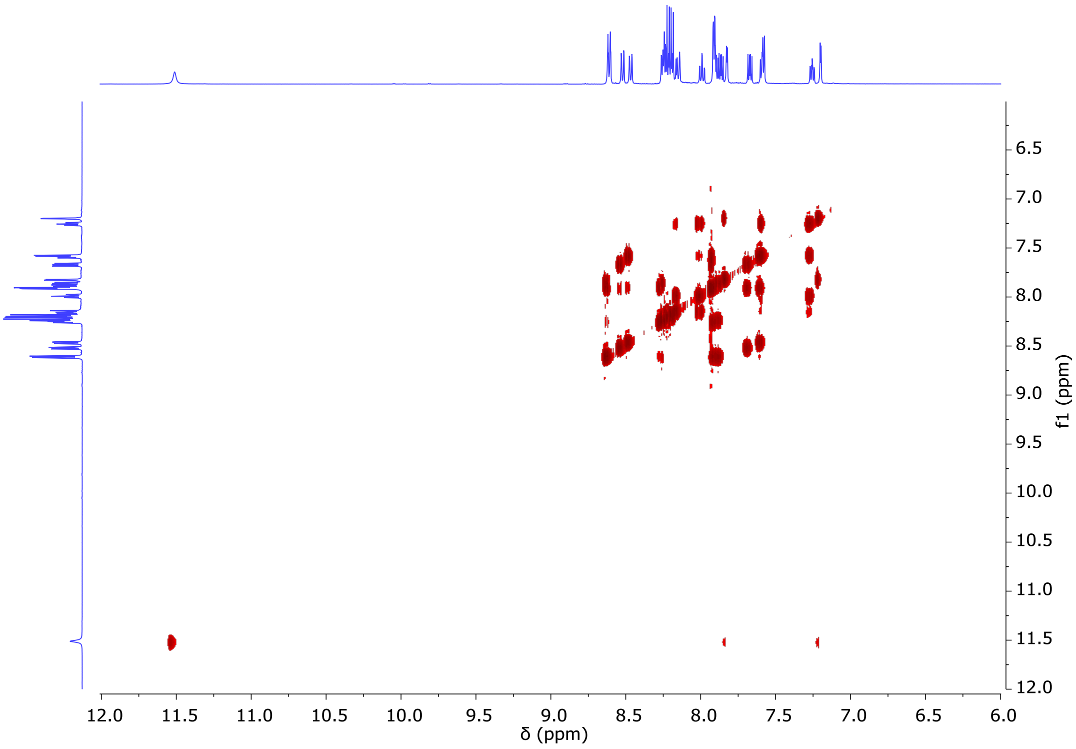
6.5
7.0
7.5
8.0
8.5
9.0
9.5
10.0
10.5
11.0
11.5
12.0
f1 (ppm)
12.0 11.5 11.0 10.5 10.0 9.5 9.0 8.5 8.0 7.5 7.0 6.5 6.0
δ (ppm)

c)

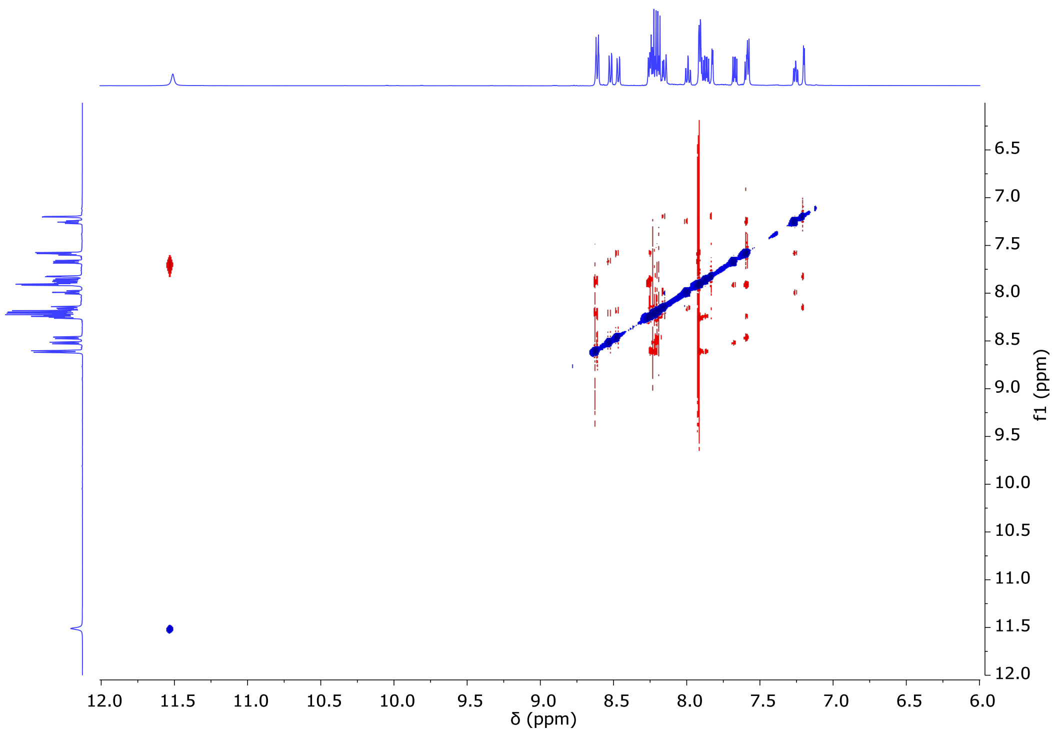
6.5
7.0
7.5
8.0
8.5
9.0
9.5
10.0
10.5
11.0
11.5
12.0
f1 (ppm)
12.0 11.5 11.0 10.5 10.0 9.5 9.0 8.5 8.0 7.5 7.0 6.5 6.0
δ (ppm)

d)

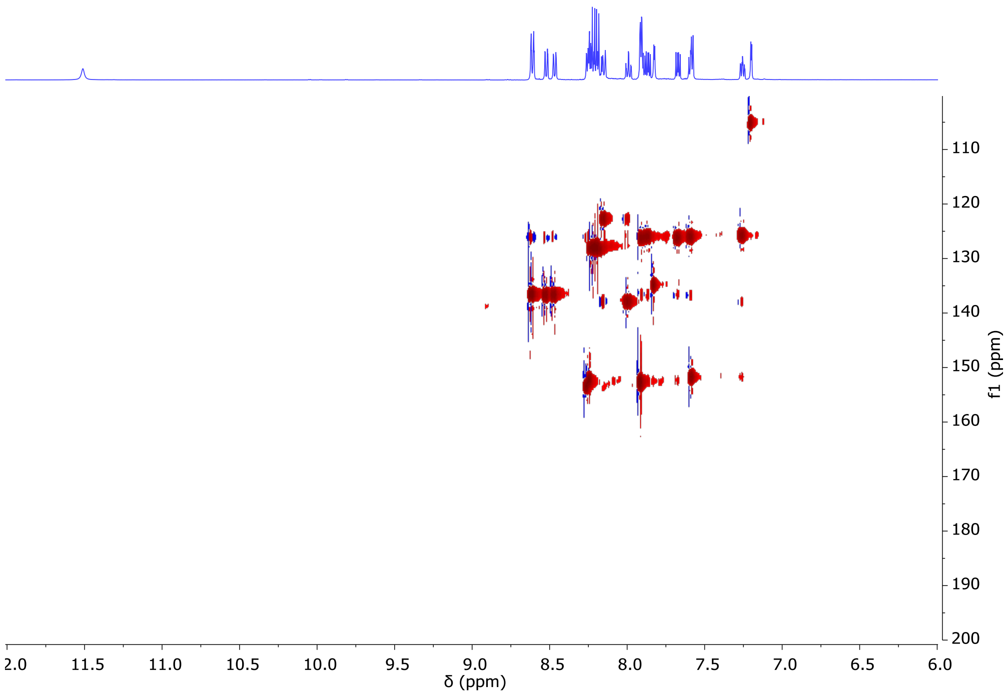
110
120
130
140
150
160
170
180
190
200
f1 (ppm)
2.0
11.5
11.0
10.5
10.0
9.5
9.0
8.5
8.0
7.5
7.0
6.5
6.0
δ (ppm)

e)

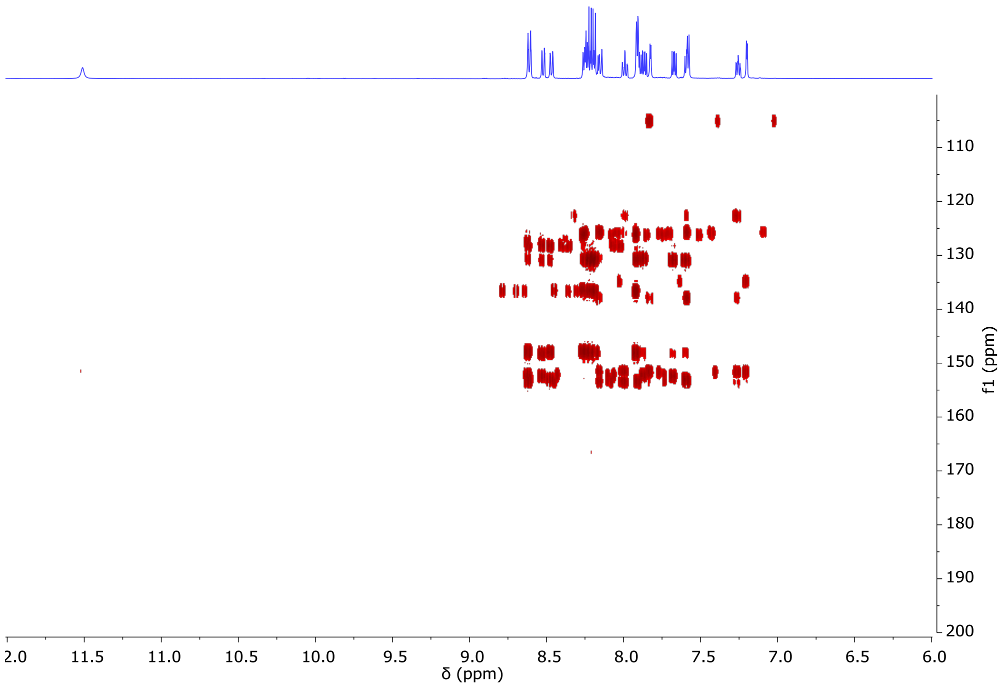
110
120
130
140
150
160
170
180
190
200
f1 (ppm)
2.0
11.5
11.0
10.5
10.0
9.5
9.0
8.5
8.0
7.5
7.0
6.5
6.0
δ (ppm)

Figure S6. NMR spectra of Ru(II) complex **3,** 400 MHz, CD_2Cl_2: a) ^{1}H-NMR b) ^{13}C-NMR; c) COSY; d) NOESY; e) ^{1}H-^{13}C HSQC; f) ^{1}H-^{13}C HMBC.

a)

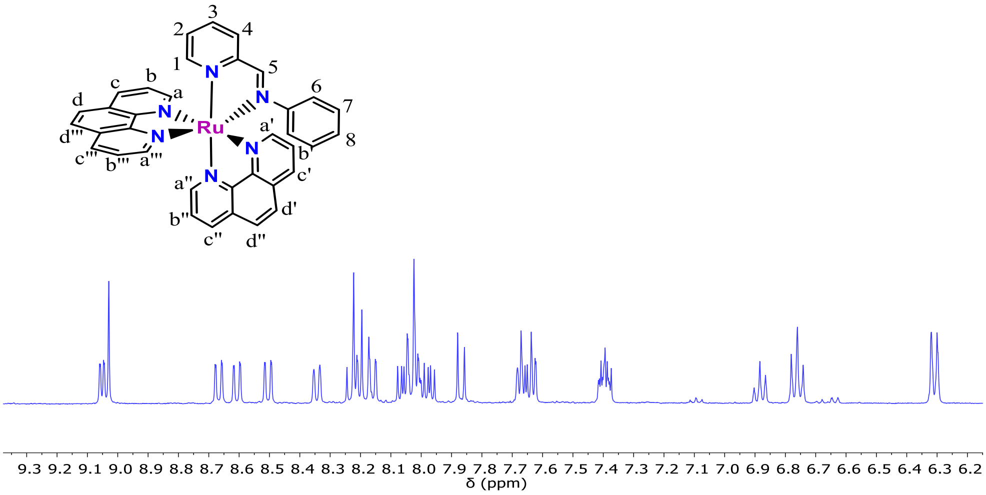

b)

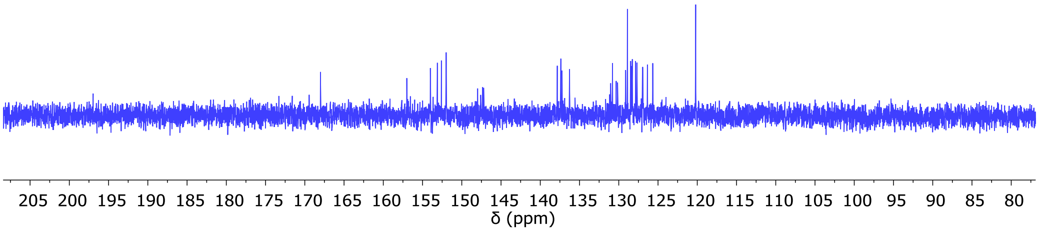

c)

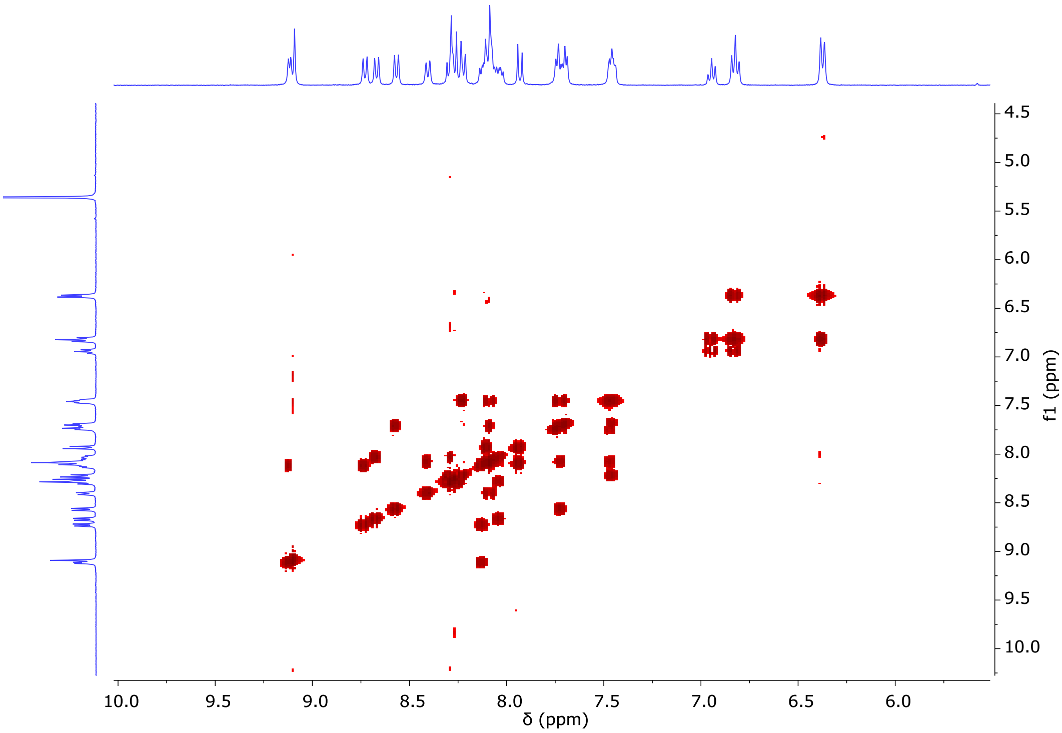

4.5
5.0
5.5
6.0
6.5
7.0
7.5
8.0
8.5
9.0
9.5
10.0
f1 (ppm)
10.0
9.5
9.0
8.5
8.0
7.5
7.0
6.5
6.0
δ (ppm)

d)

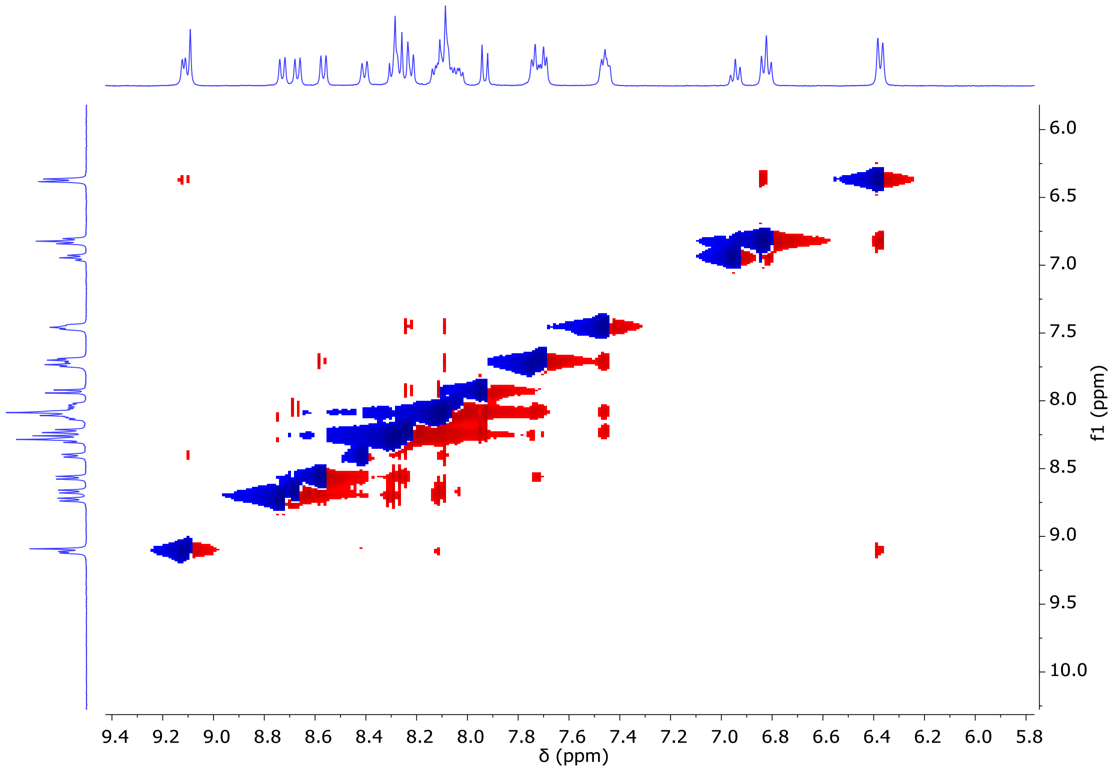

6.0
6.5
7.0
7.5
8.0
8.5
9.0
9.5
10.0
f1 (ppm)
9.4 9.2 9.0 8.8 8.6 8.4 8.2 8.0 7.8 7.6 7.4 7.2 7.0 6.8 6.6 6.4 6.2 6.0 5.8
δ (ppm)

e)

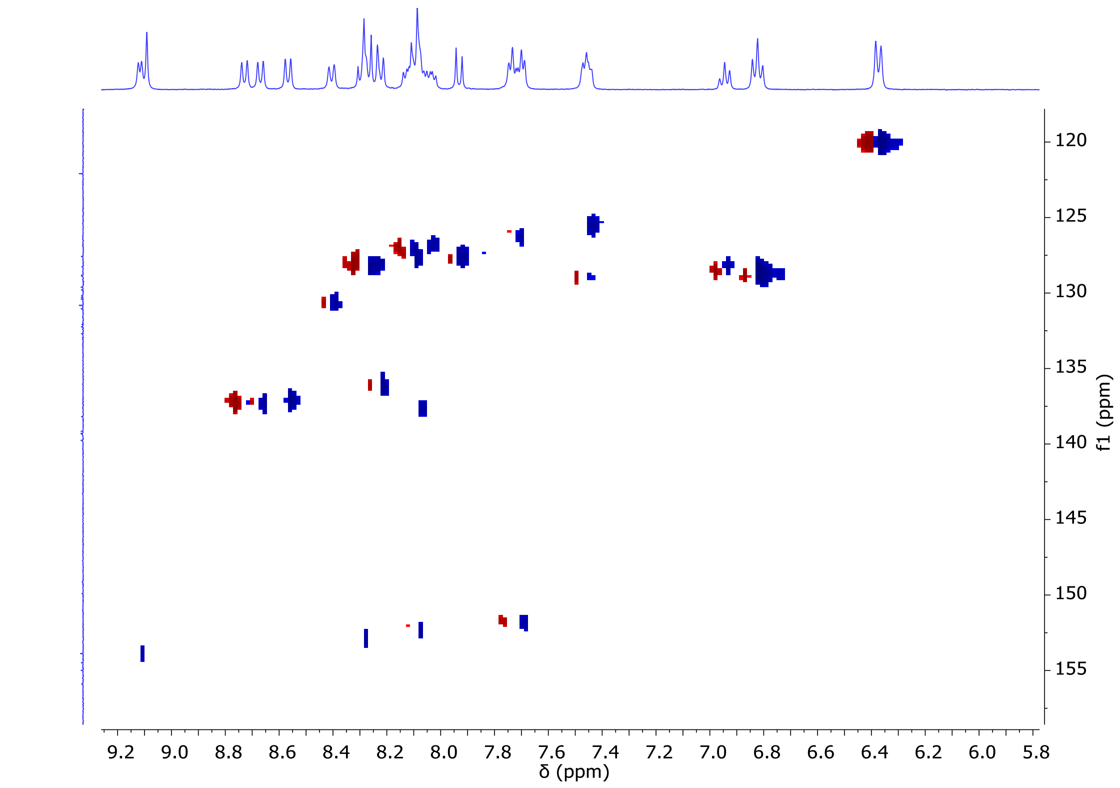

120
125
130
135
140
145
150
155
f1 (ppm)
9.2 9.0 8.8 8.6 8.4 8.2 8.0 7.8 7.6 7.4 7.2 7.0 6.8 6.6 6.4 6.2 6.0 5.8
δ (ppm)

f)

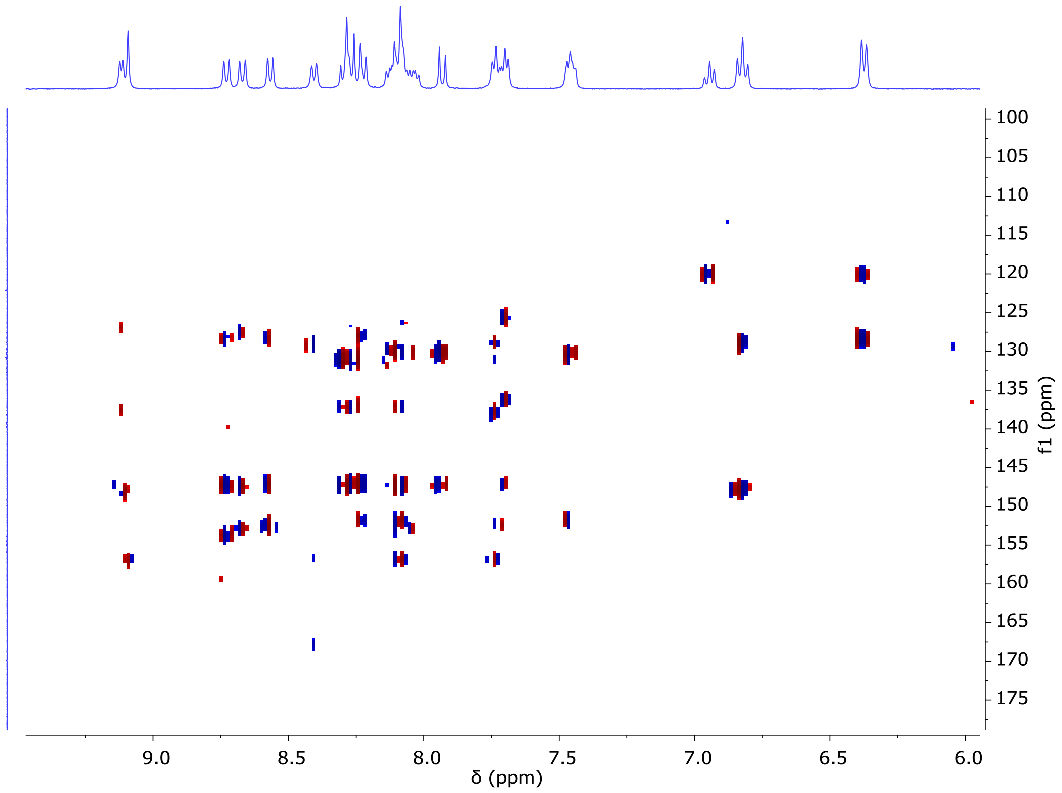
f1 (ppm)
δ (ppm)
100
105
110
115
120
125
130
135
140
145
150
155
160
165
170
175
9.0
8.5
8.0
7.5
7.0
6.5
6.0

Figure S7. NMR spectra of Ru(II) complex **4,** 400 MHz, CD_2Cl_2: a) ^{1}H-NMR b) ^{13}C-NMR; c) COSY; d) NOESY; e) ^{1}H-^{13}C HSQC; f) ^{1}H-^{13}C HMBC.

a)

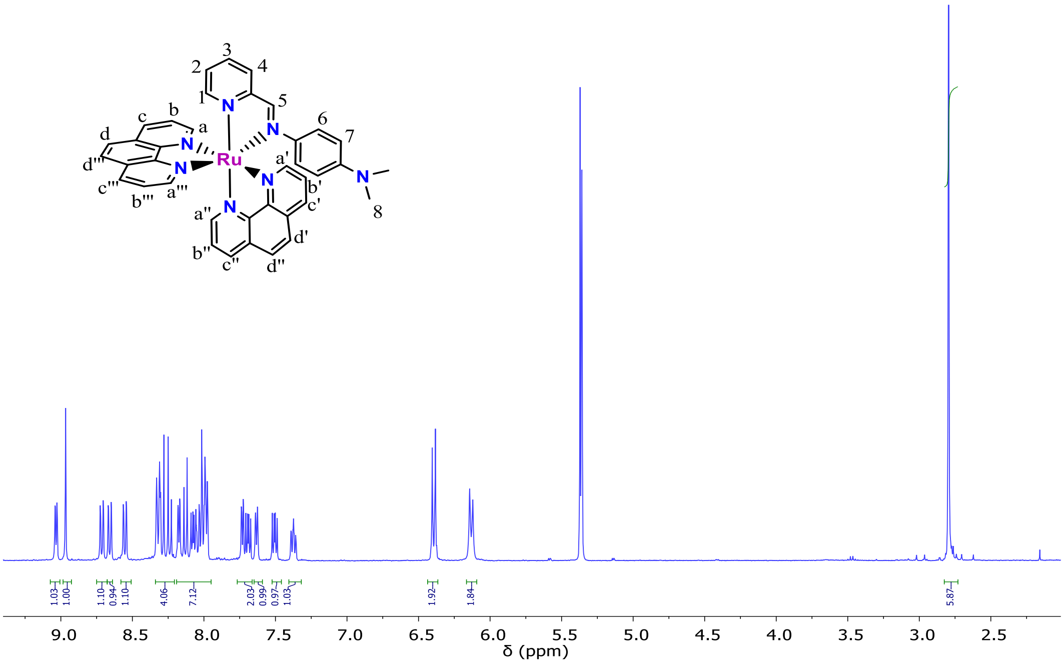

b)

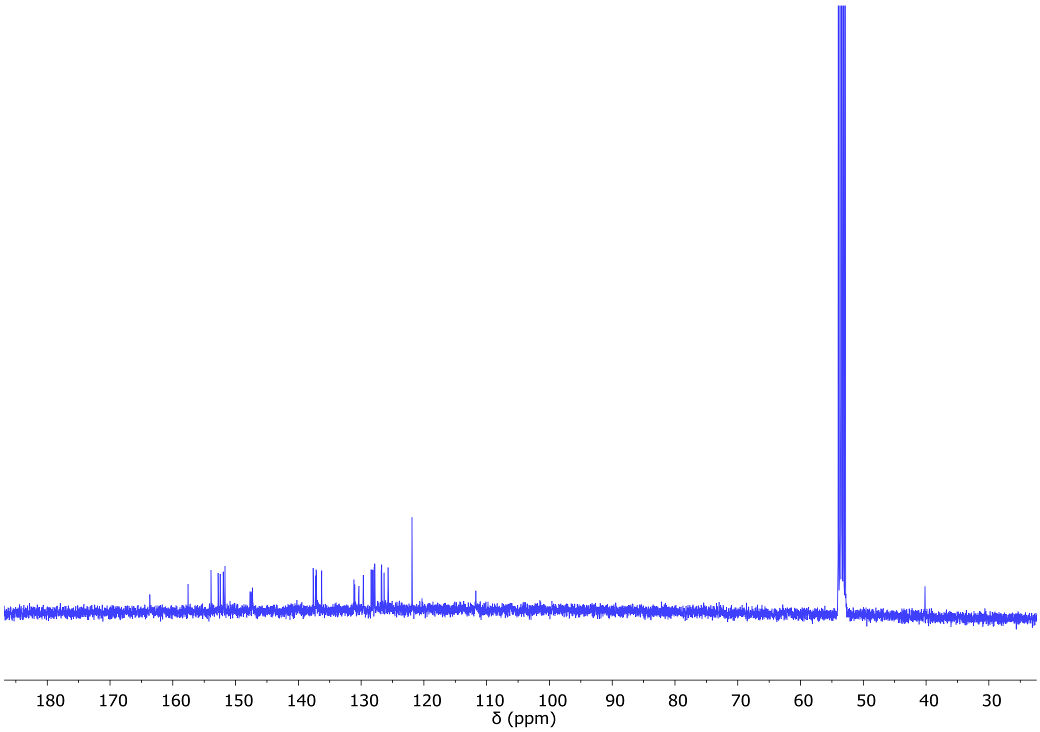

c)

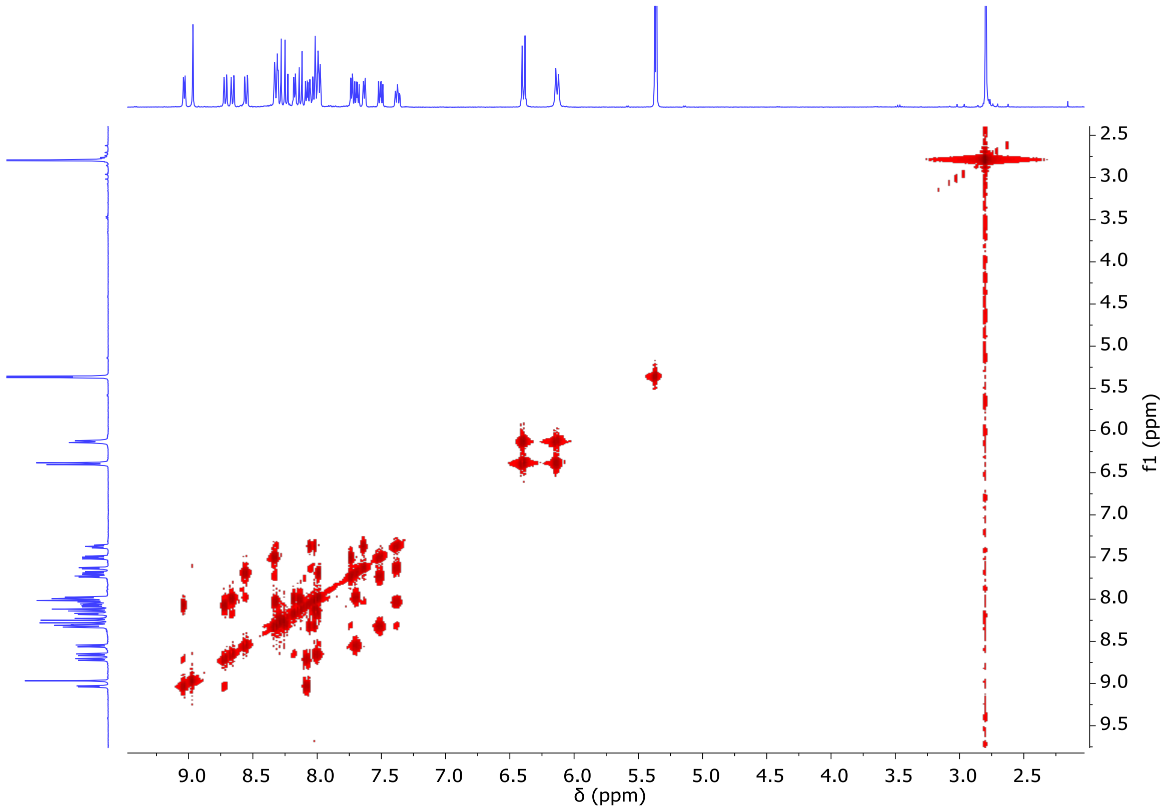
9.0
8.5
8.0
7.5
7.0
6.5
6.0
5.5
5.0
4.5
4.0
3.5
3.0
2.5
δ (ppm)
f1 (ppm)

d)

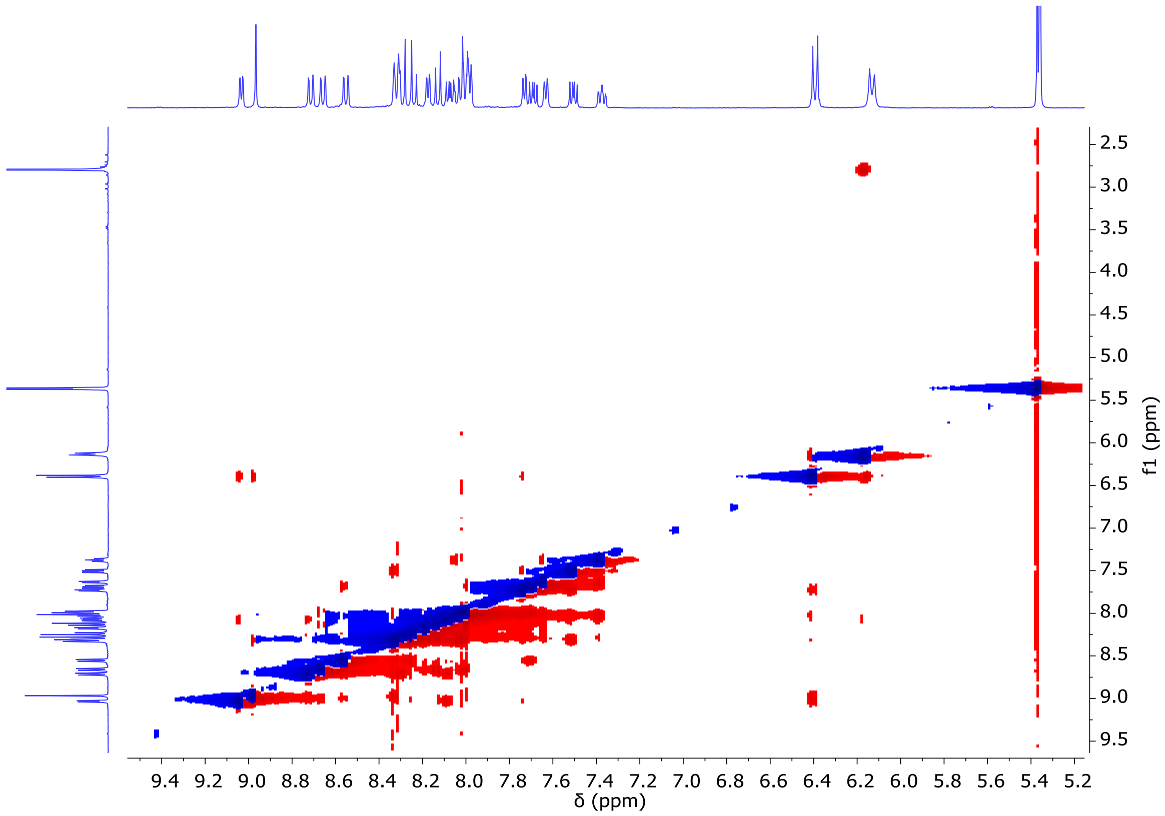
9.4
9.2
9.0
8.8
8.6
8.4
8.2
8.0
7.8
7.6
7.4
7.2
7.0
6.8
6.6
6.4
6.2
6.0
5.8
5.6
5.4
5.2
δ (ppm)
f1 (ppm)

e)

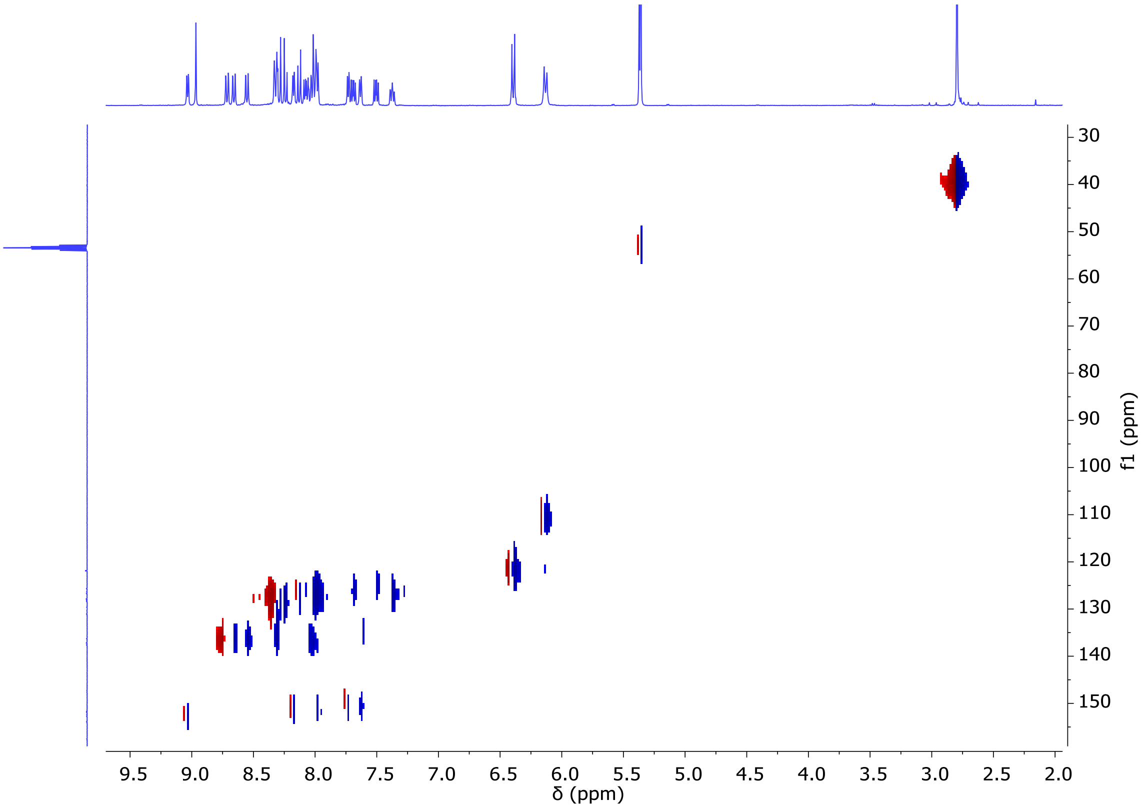

9.5 9.0 8.5 8.0 7.5 7.0 6.5 6.0 5.5 5.0 4.5 4.0 3.5 3.0 2.5 2.0
δ (ppm)
30 40 50 60 70 80 90 100 110 120 130 140 150
f1 (ppm)

f)

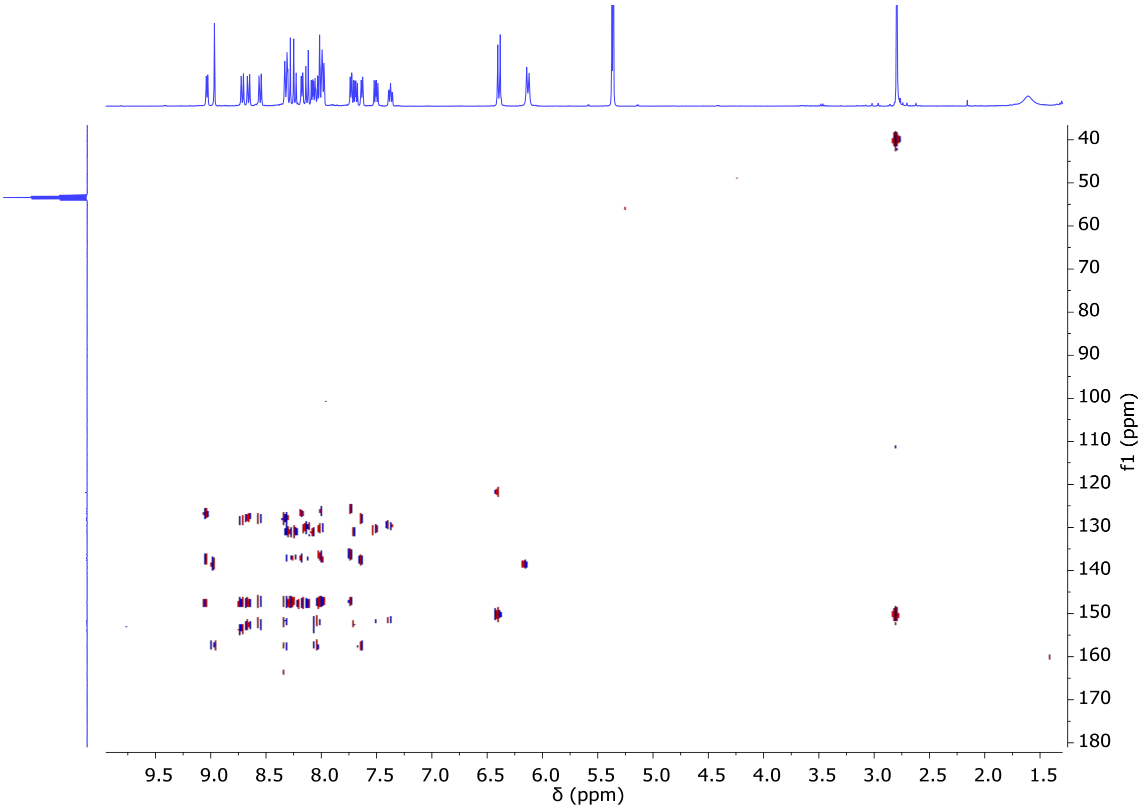

9.5 9.0 8.5 8.0 7.5 7.0 6.5 6.0 5.5 5.0 4.5 4.0 3.5 3.0 2.5 2.0 1.5
δ (ppm)
40 50 60 70 80 90 100 110 120 130 140 150 160 170 180
f1 (ppm)

Figure S8. NMR spectra of Ru(II) complex **5,** 400 MHz, CD_2Cl_2: a) ^{13}C-NMR; b) COSY; c) NOESY; d) ^{1}H-^{13}C HSQC; e) ^{1}H-^{13}C HMBC.

a)

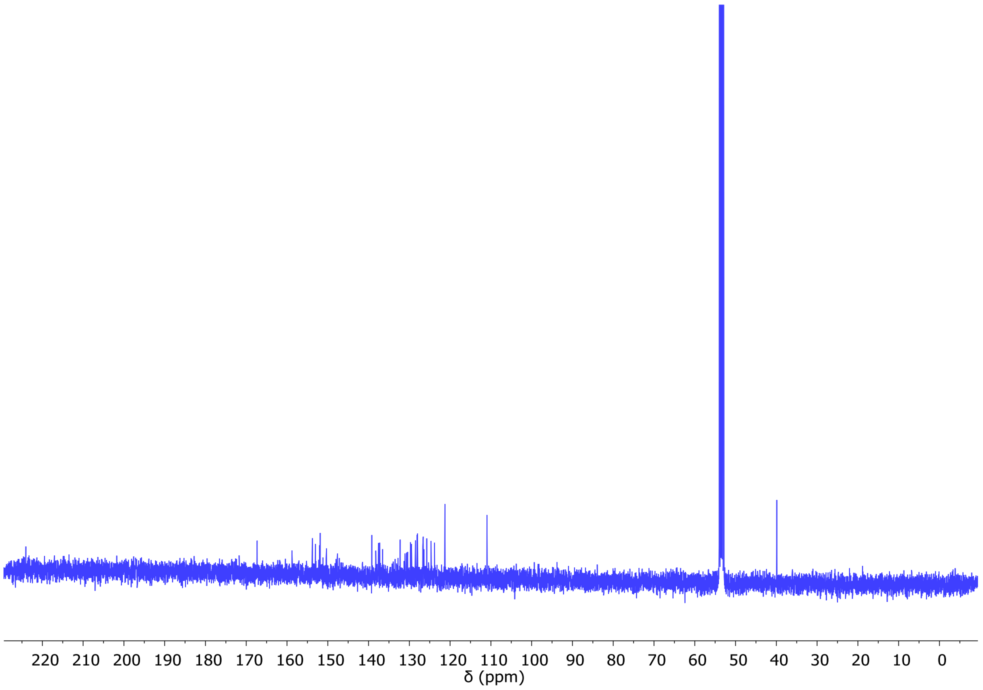

b)

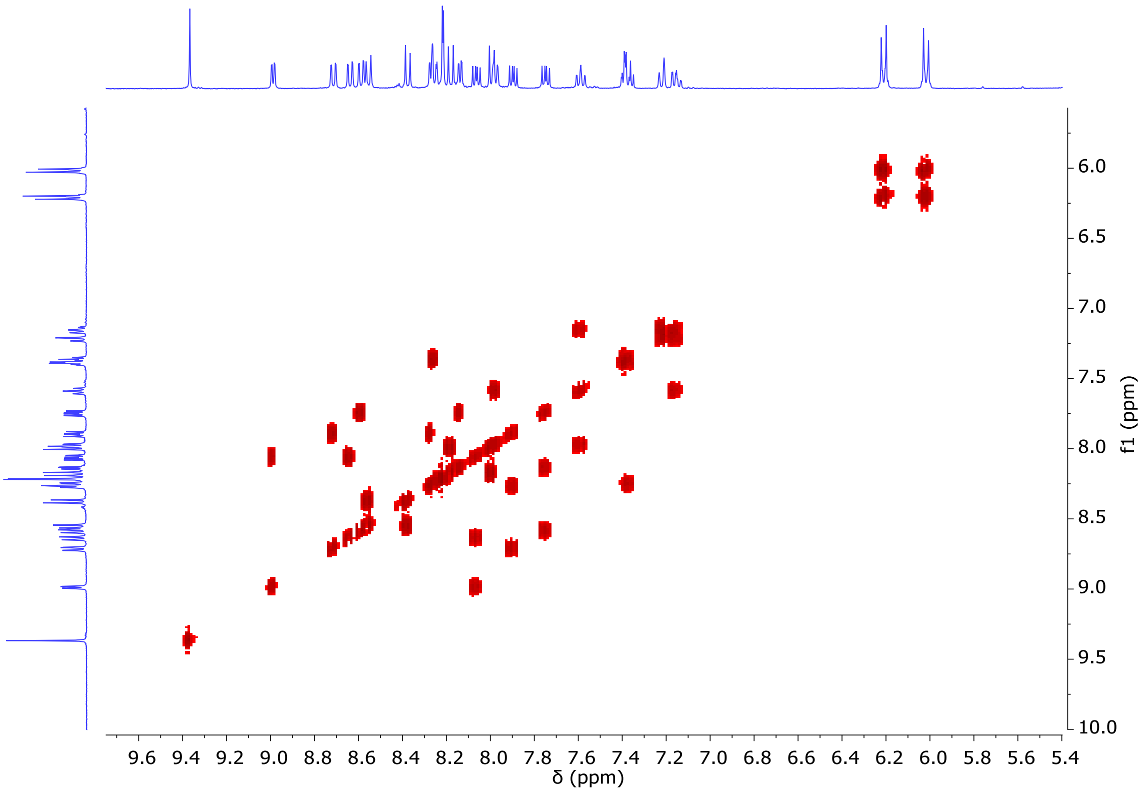

c)

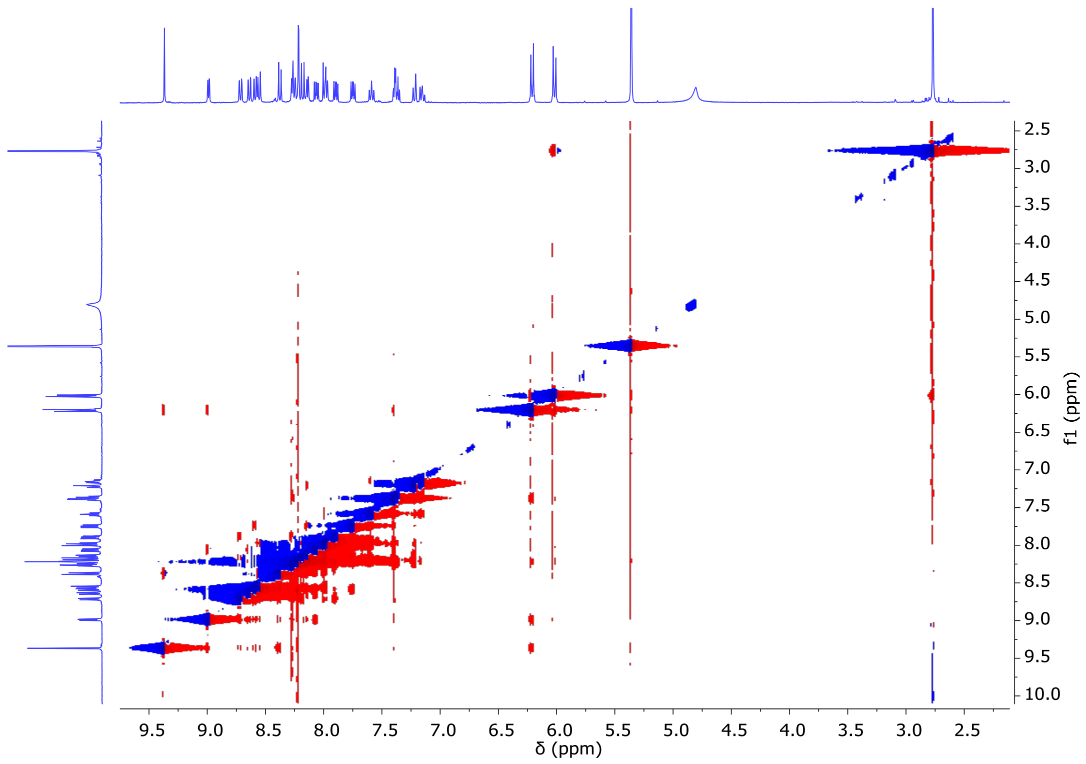

d)

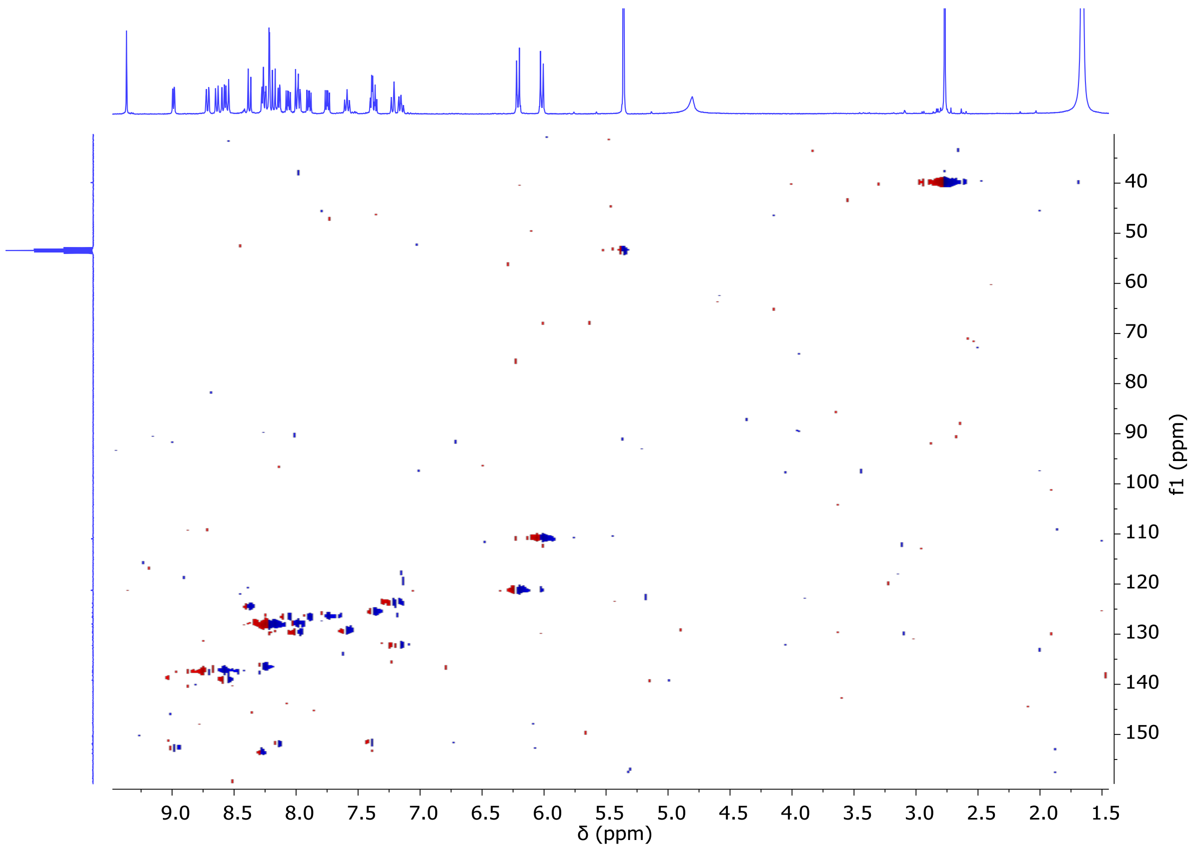

e)

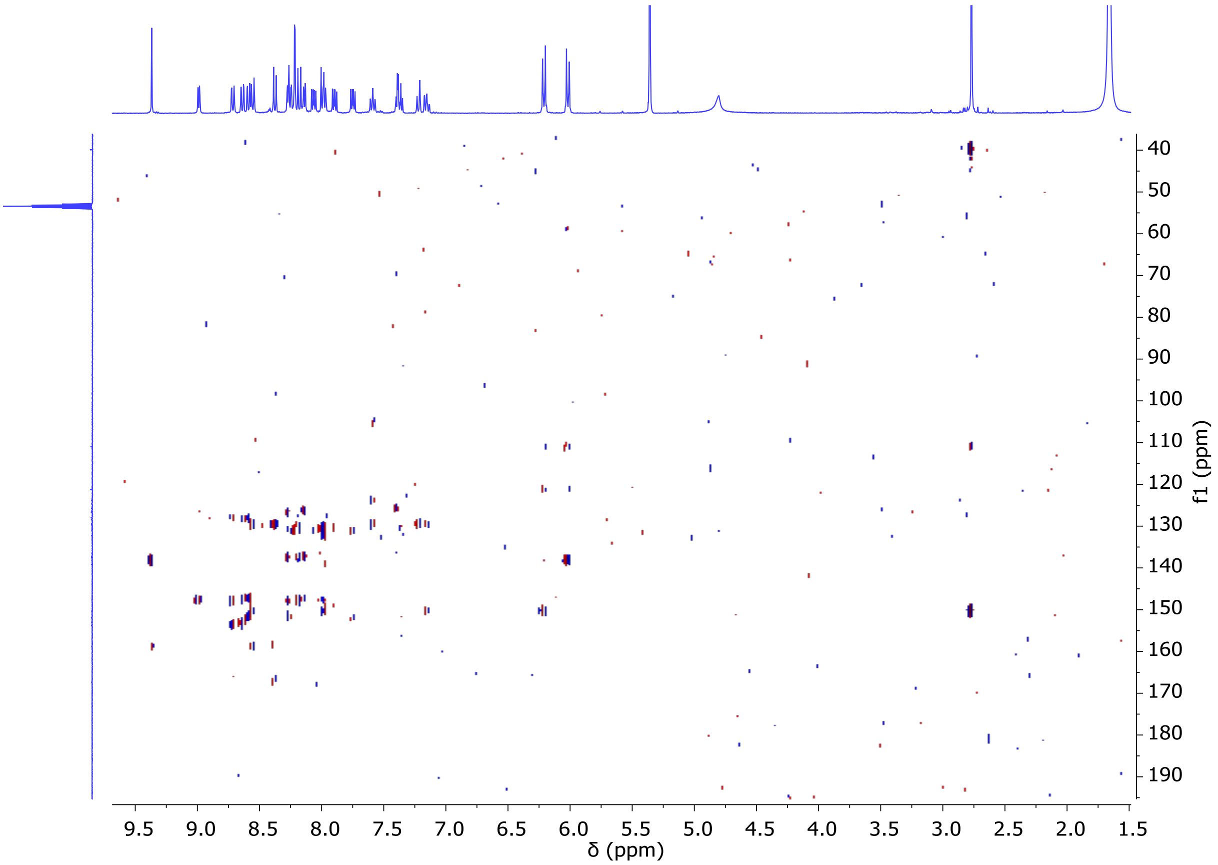

9.5
9.0
8.5
8.0
7.5
7.0
6.5
6.0
5.5
5.0
4.5
4.0
3.5
3.0
2.5
2.0
1.5
δ (ppm)
40
50
60
70
80
90
100
110
120
130
140
150
160
170
180
190
f1 (ppm)

Figure S9. NMR spectra of Ru(II) complex **6,** 400 MHz, CH_3CN: a) ^{1}H-NMR b) ^{13}C-NMR; c) COSY; d) NOESY; e) ^{1}H-^{13}C HSQC; f) ^{1}H-^{13}C HMBC.

a)

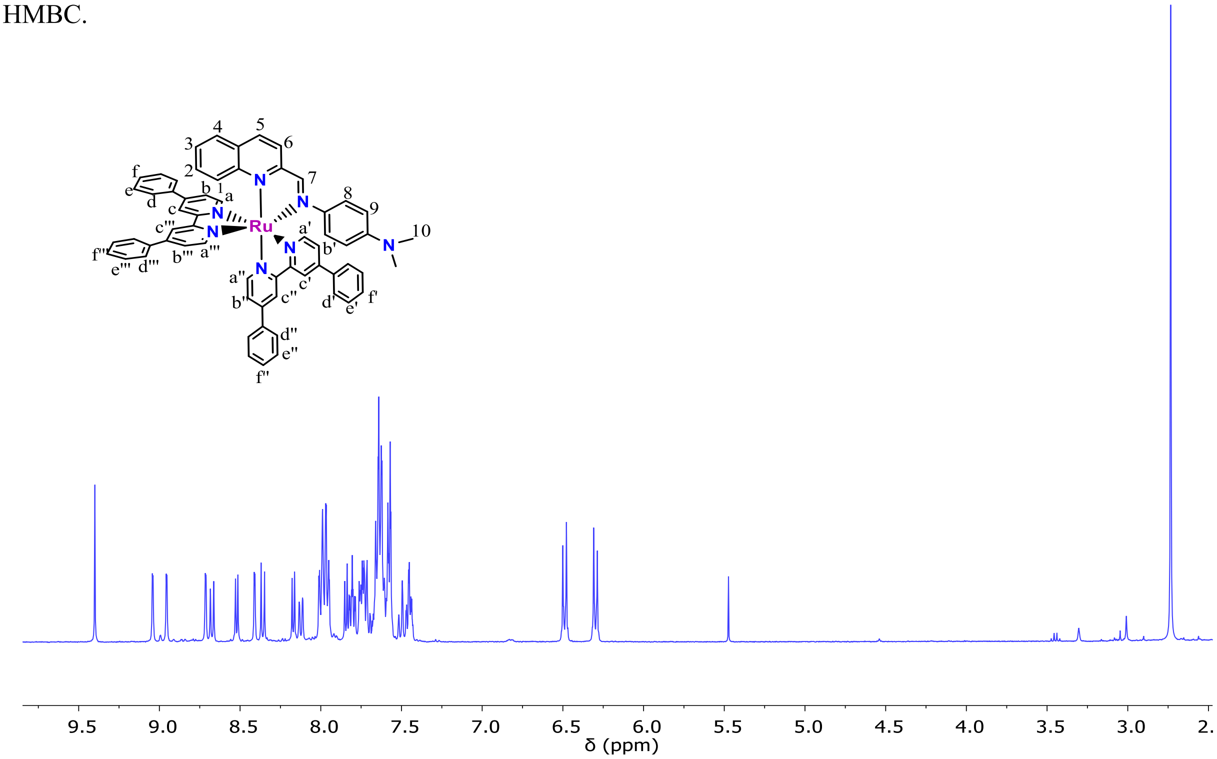

b)

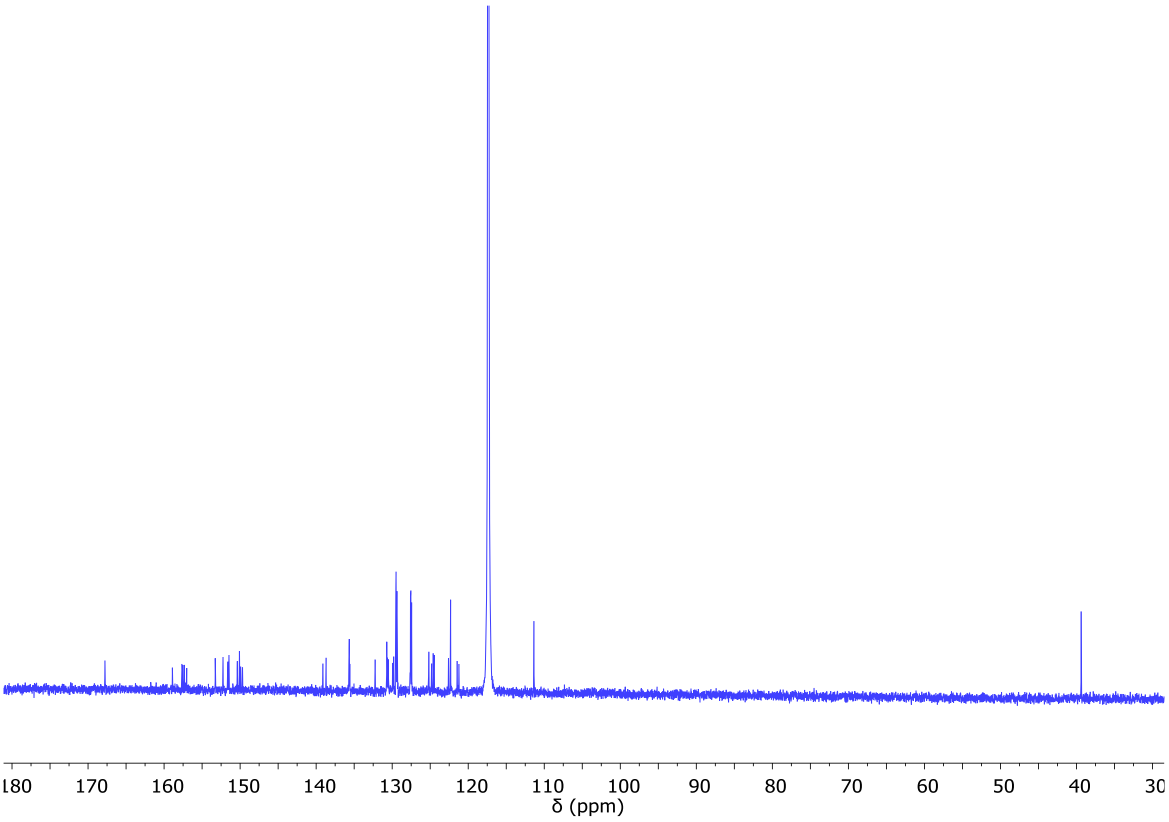

c)

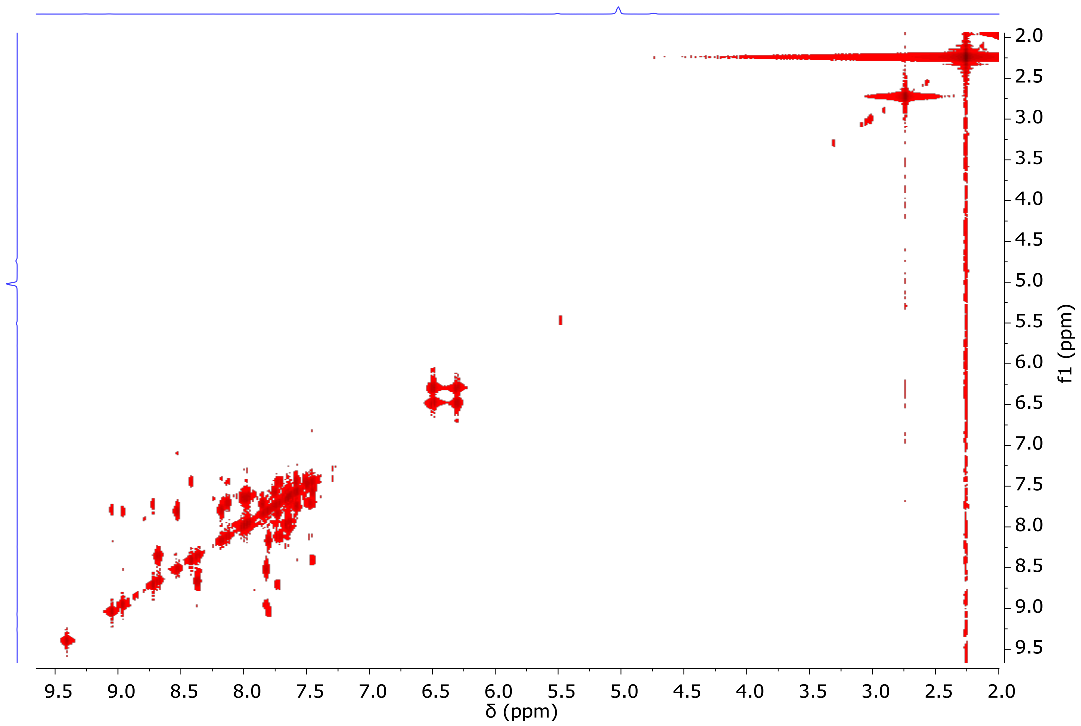

d)

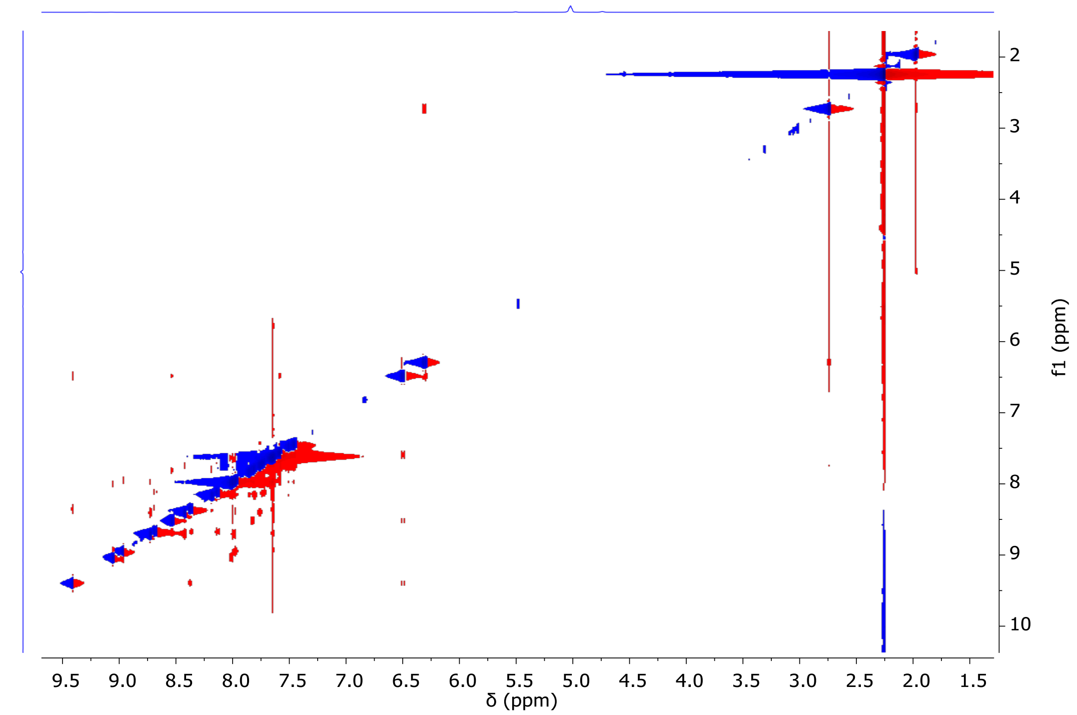

e)

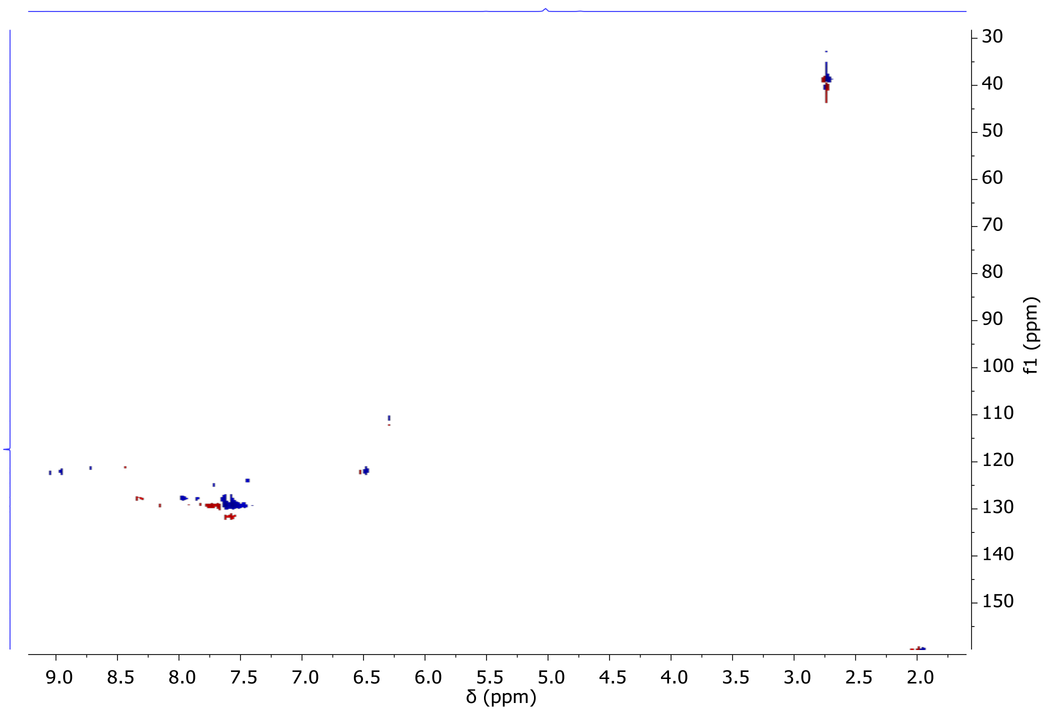

30
40
50
60
70
80
90
100
110
120
130
140
150
f1 (ppm)
9.0
8.5
8.0
7.5
7.0
6.5
6.0
5.5
5.0
4.5
4.0
3.5
3.0
2.5
2.0
δ (ppm)

f)

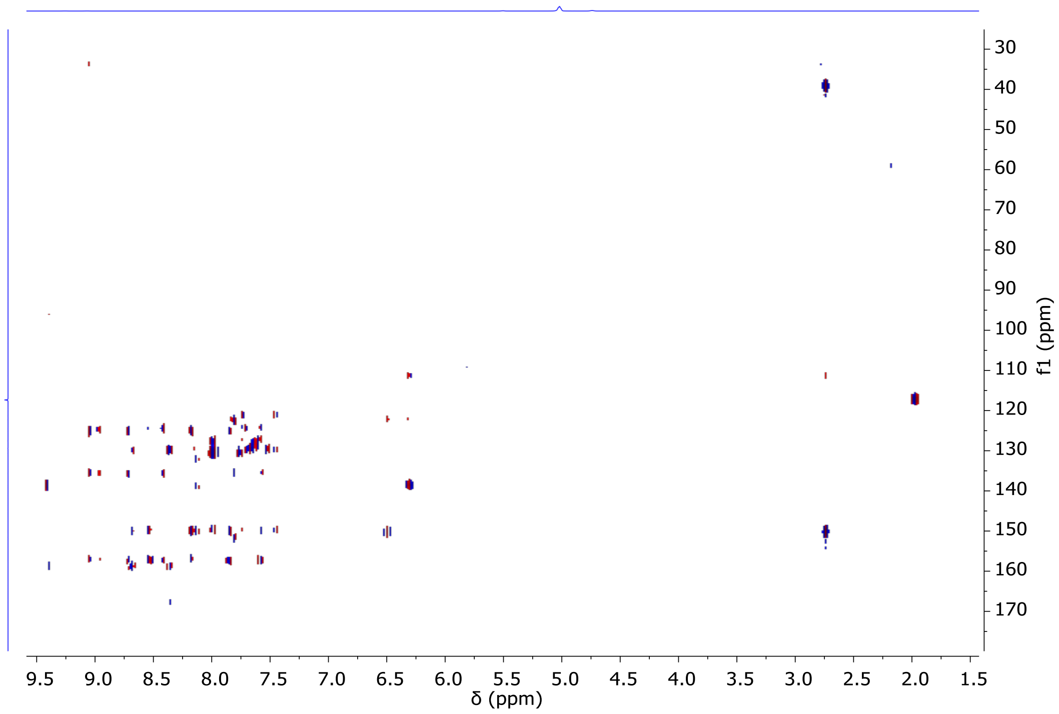

30
40
50
60
70
80
90
100
110
120
130
140
150
160
170
f1 (ppm)
9.5
9.0
8.5
8.0
7.5
7.0
6.5
6.0
5.5
5.0
4.5
4.0
3.5
3.0
2.5
2.0
1.5
δ (ppm)

Figure S10. ESI-MS spectra of complexes, a) **2**; b) **3**; c) **4**; d) **5**; e) **6**.

a)

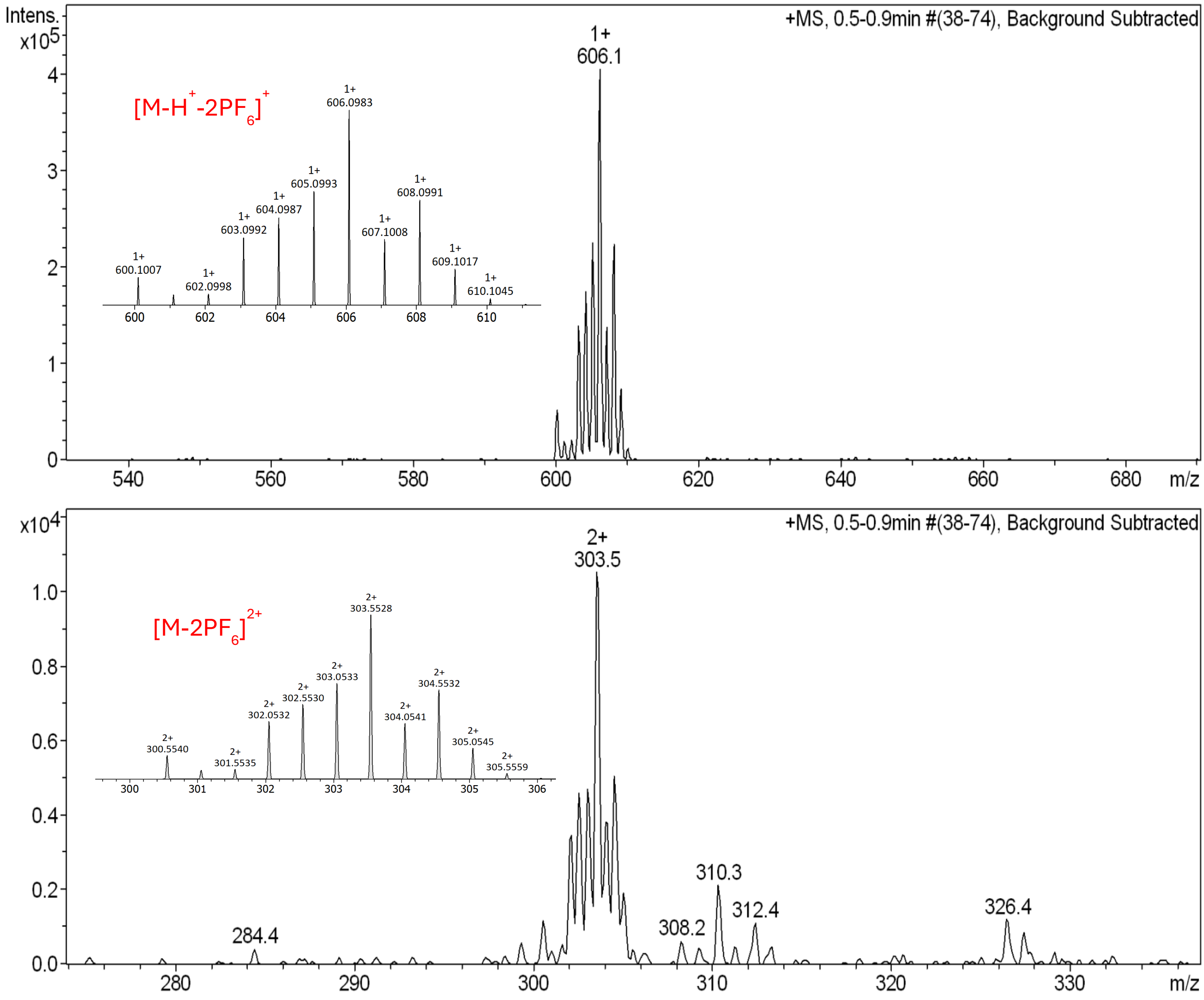

b)

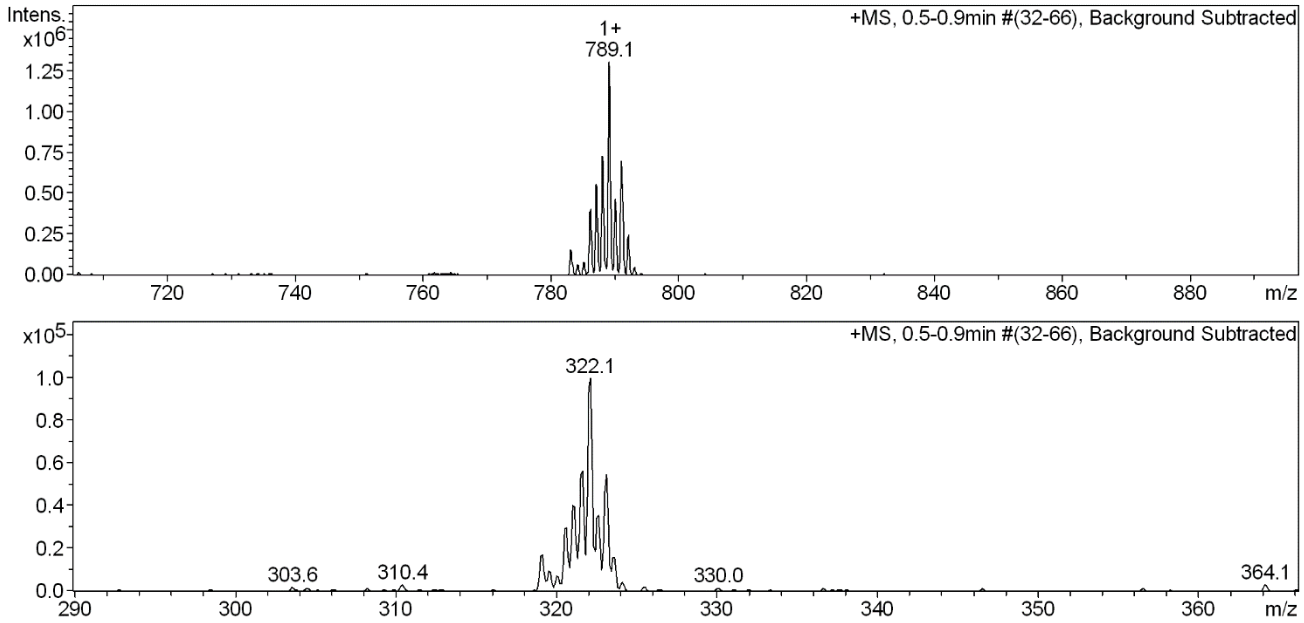
Intens.
x10^6
+MS, 0.5-0.9min #(32-66), Background Subtracted
1+
789.1
m/z
x10^5
+MS, 0.5-0.9min #(32-66), Background Subtracted
322.1
303.6
310.4
330.0
364.1
m/z

c)

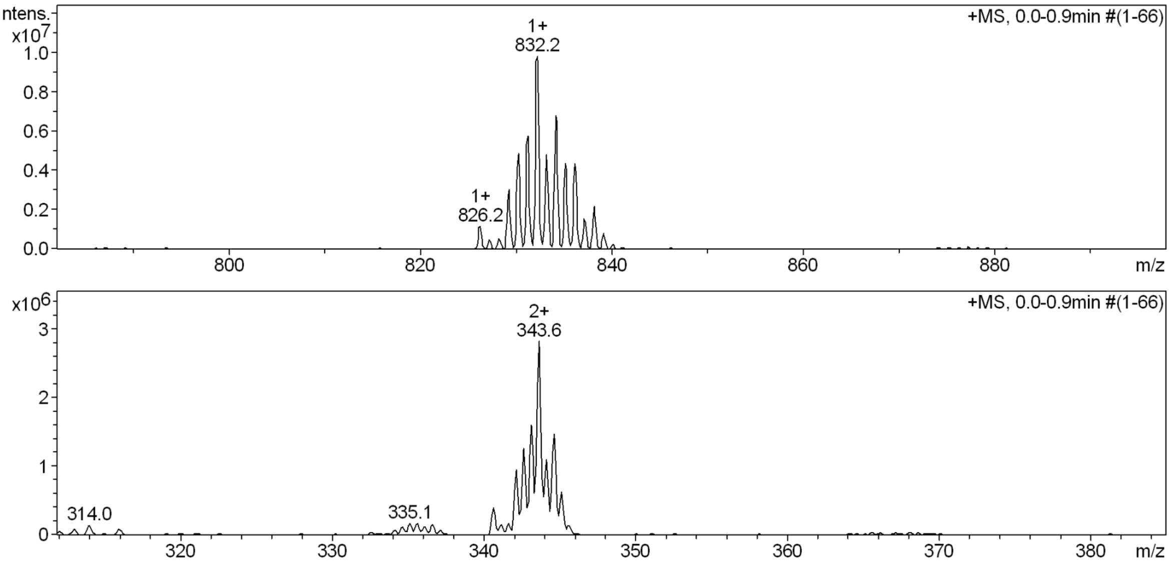
Intens.
x10^7
+MS, 0.0-0.9min #(1-66)
1+
832.2
1+
826.2
m/z
x10^6
+MS, 0.0-0.9min #(1-66)
2+
343.6
314.0
335.1
m/z

d)

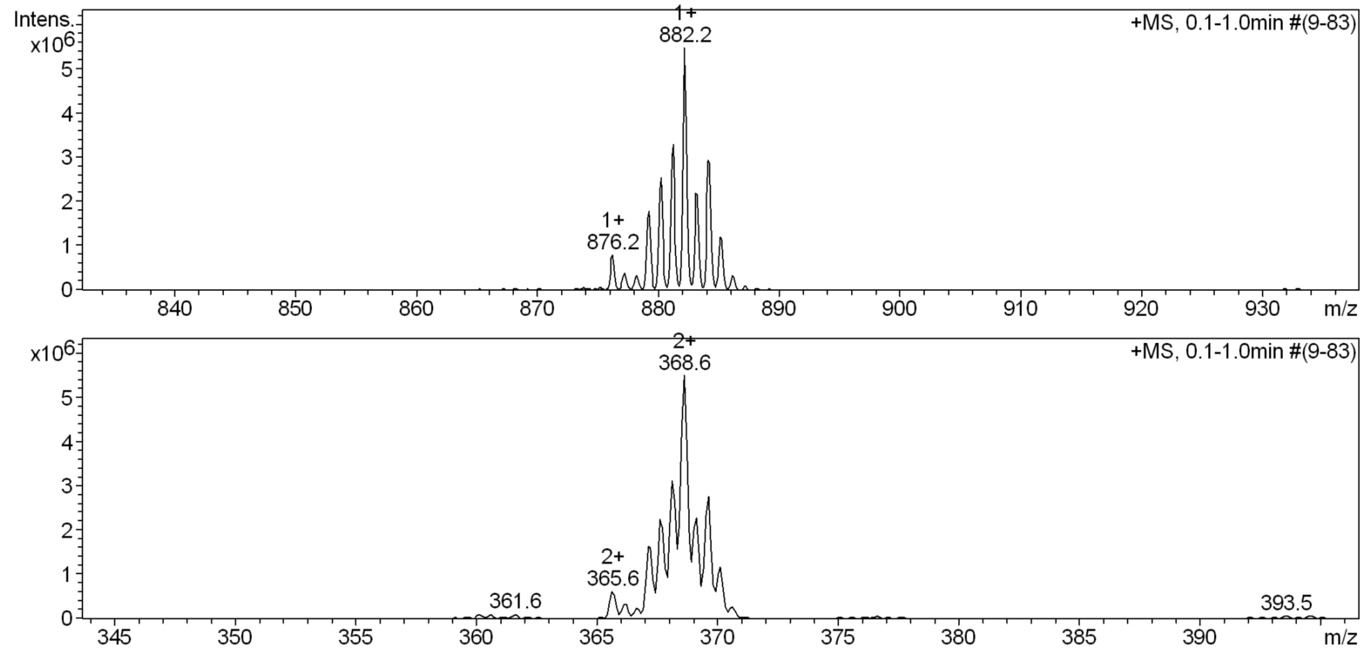
Intens.
x10^6
+MS, 0.1-1.0min #(9-83)
1+
882.2
1+
876.2
m/z
x10^6
+MS, 0.1-1.0min #(9-83)
2+
368.6
2+
365.6
361.6
393.5
m/z

e)

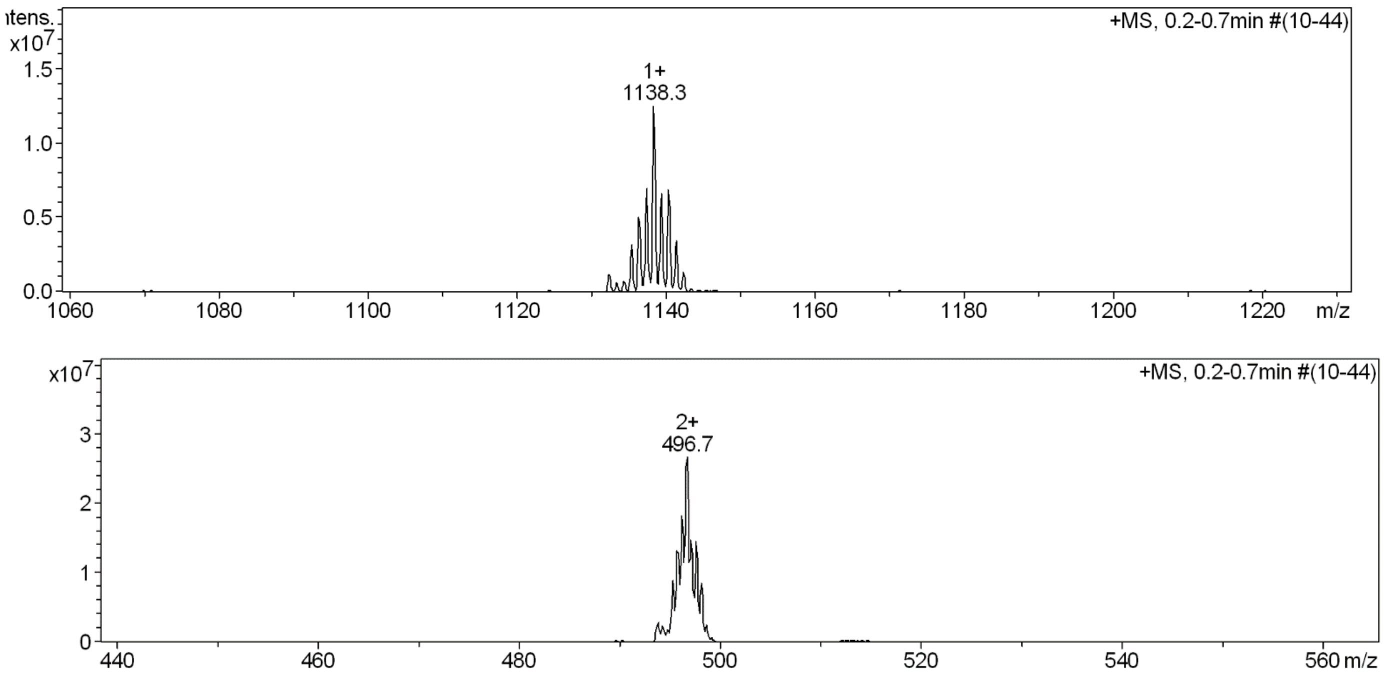
Intens.
x10^7
+MS, 0.2-0.7min #(10-44)
1+
1138.3
m/z
x10^7
+MS, 0.2-0.7min #(10-44)
2+
496.7
m/z

Figure S11. Normalized absorption and emission spectra of complexes **2–6** in air-saturated solvents: methanol (top), dichloromethane (middle), and acetonitrile (bottom).

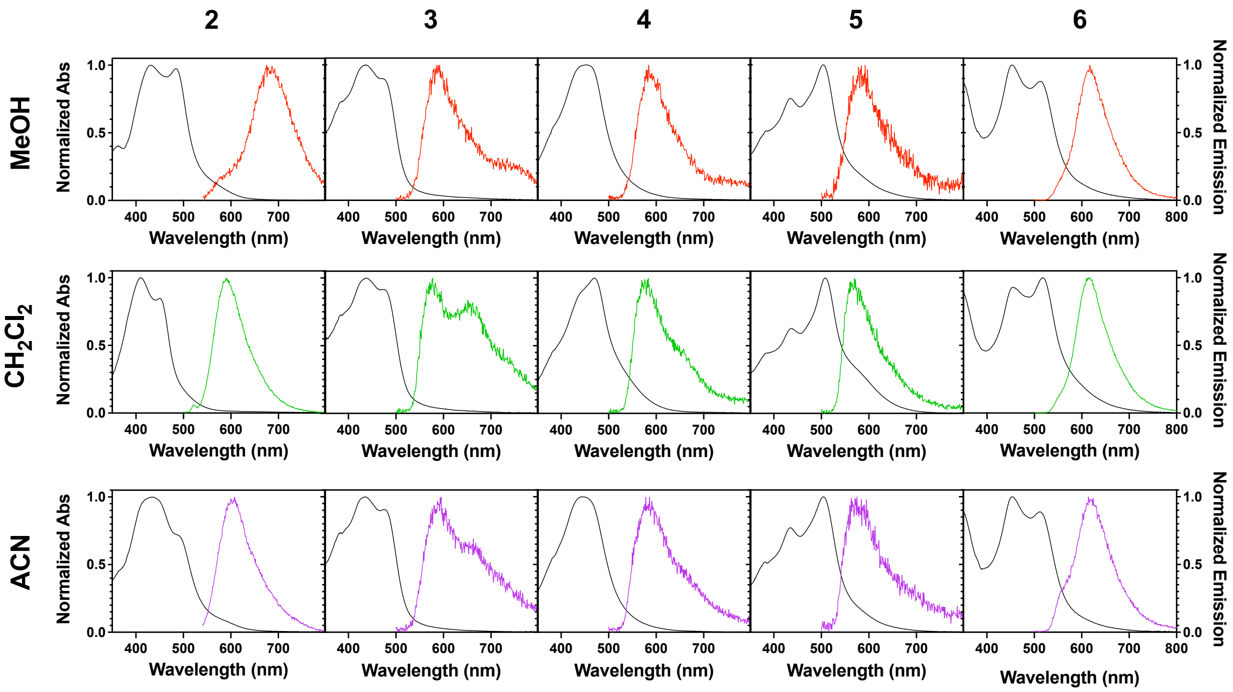

Figure S12. Frontier molecular orbitals of complexes **2** and **3**, including their energies (in eV).

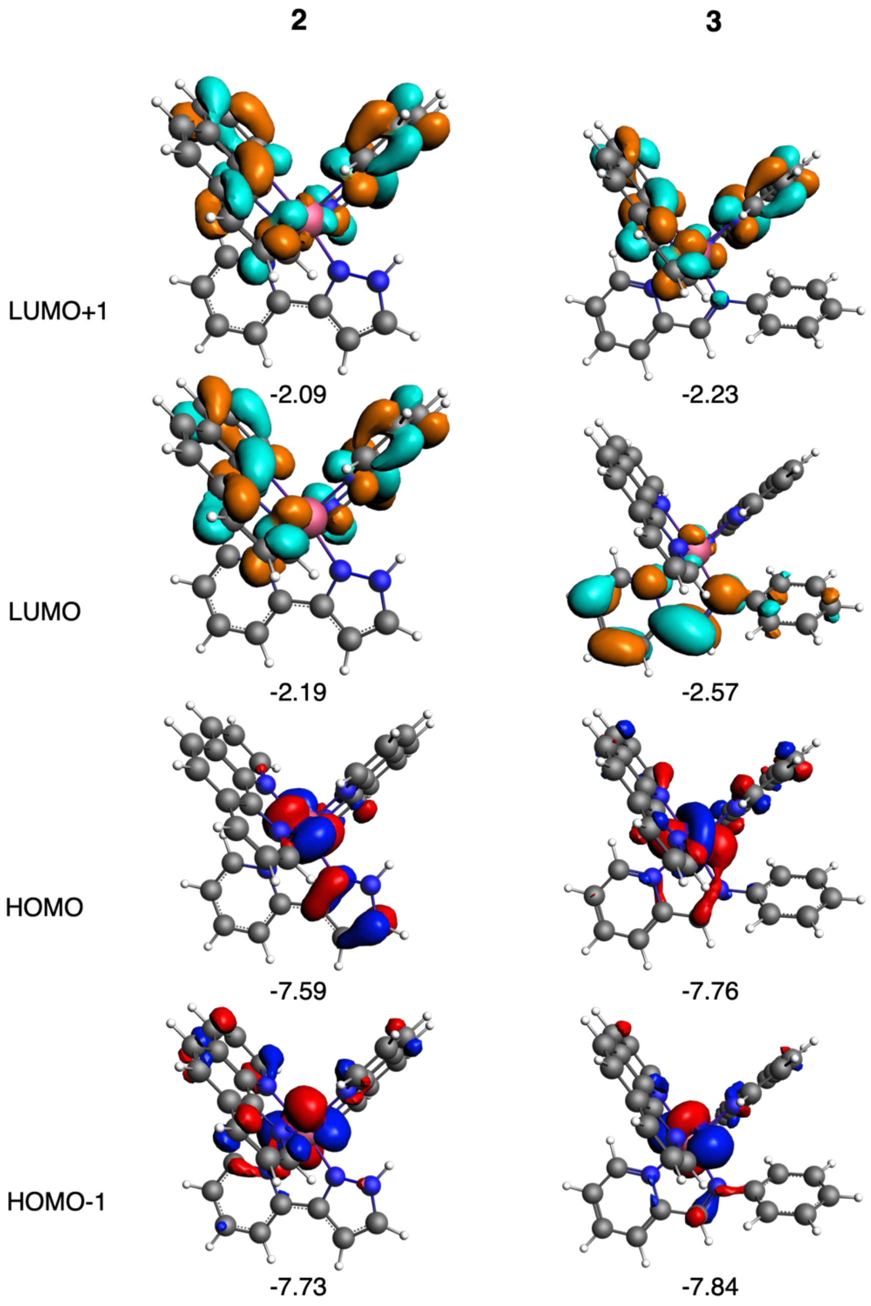

Figure S13. Computed UV-Vis spectra of **2** and **3** at CAMY-B3LYP/TZ2P level in DCM (COSMO). Involved molecular orbitals in the main absorption bands are also included, together with their energies (in eV), oscillator strengths, and their contributions of single orbital transitions.

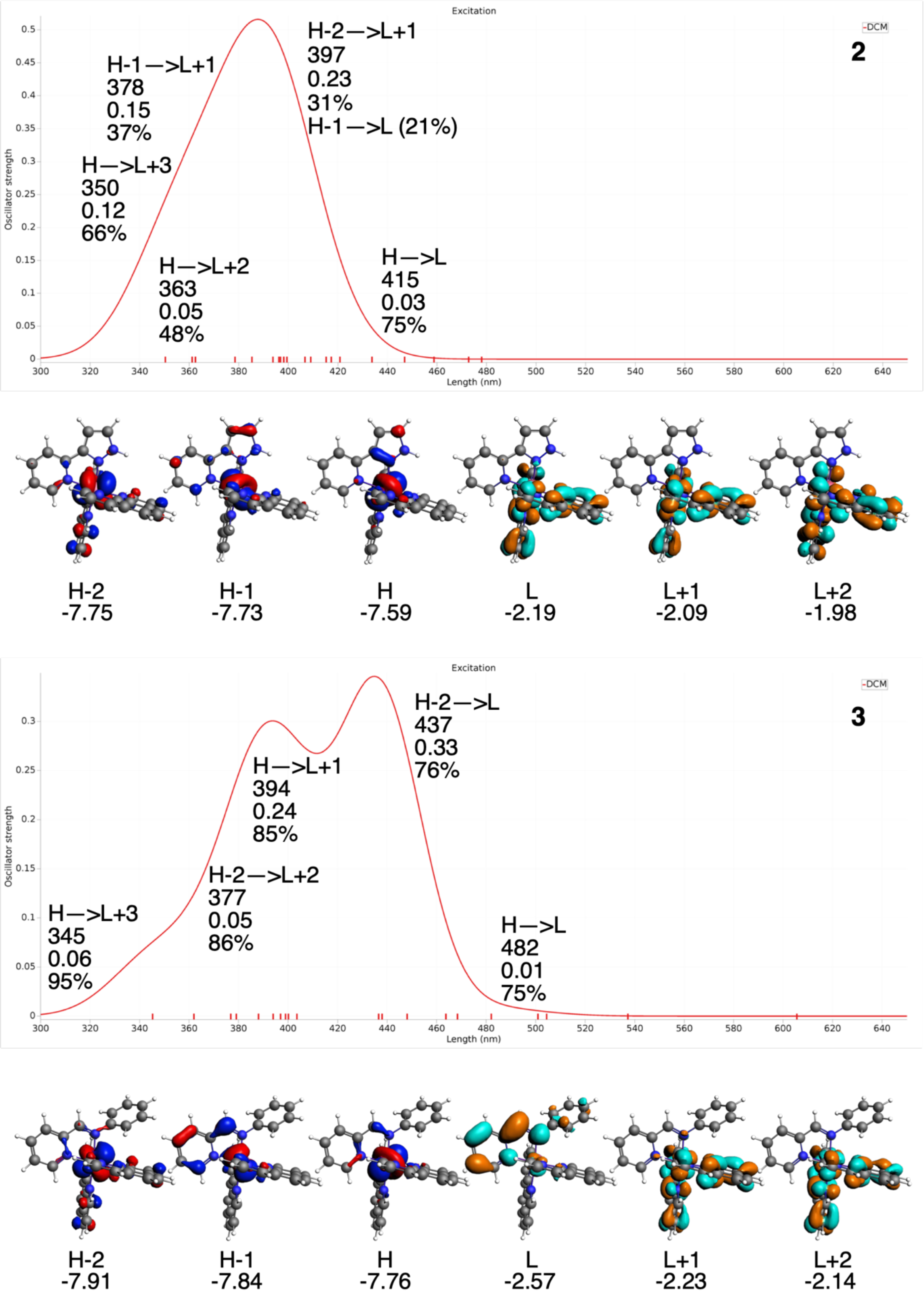

Figure S14. Computed UV-Vis spectra of **4** and **5** at CAMY-B3LYP/TZ2P level in DCM (COSMO). Involved molecular orbitals in the main absorption bands are also included, together with their energies (in eV), oscillator strengths, and their contributions of single orbital transitions.

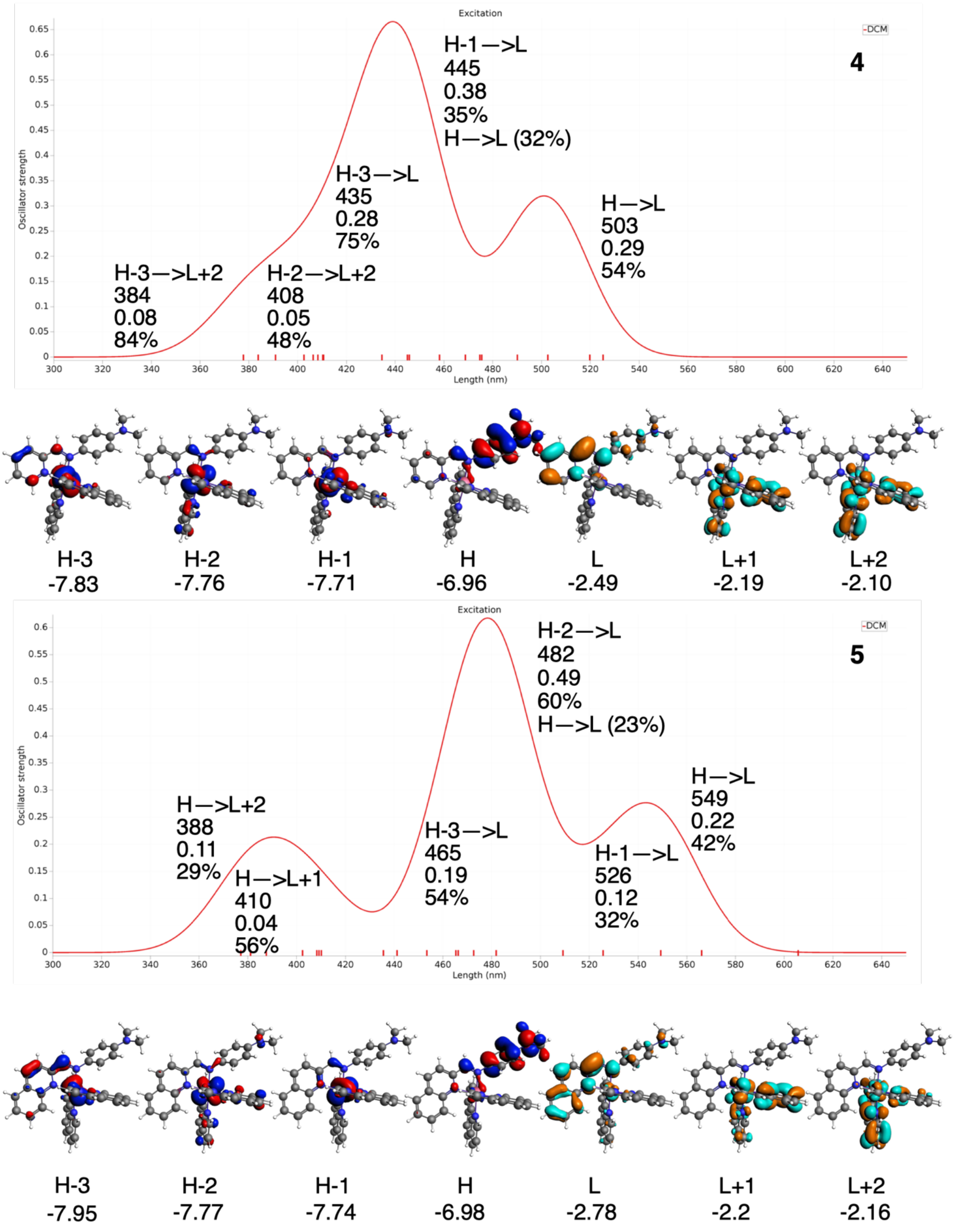

Figure S15. Computed UV-Vis spectra of **6** at CAMY-B3LYP/TZ2P level in DCM (COSMO). Involved molecular orbitals in the main absorption bands are also included, together with their energies (in eV), oscillator strengths, and their contributions of single orbital transitions. As a reference, HOMO-LUMO gaps amount to 5.4, 5.2, 4.5, 4.2 and 4.2 eV for **2**, **3**, **4**, **5** and **6**, respectively, computed at the same level of theory, being HOMO destabilized (-7.58, -7.76, -6.96, -6.98, -6.99 eV from **2** to **6**) and LUMO stabilized (-2.19, -2.57, -2.49, -2.78, -2.75 eV from **2** to **6**) from **2** to **6**.

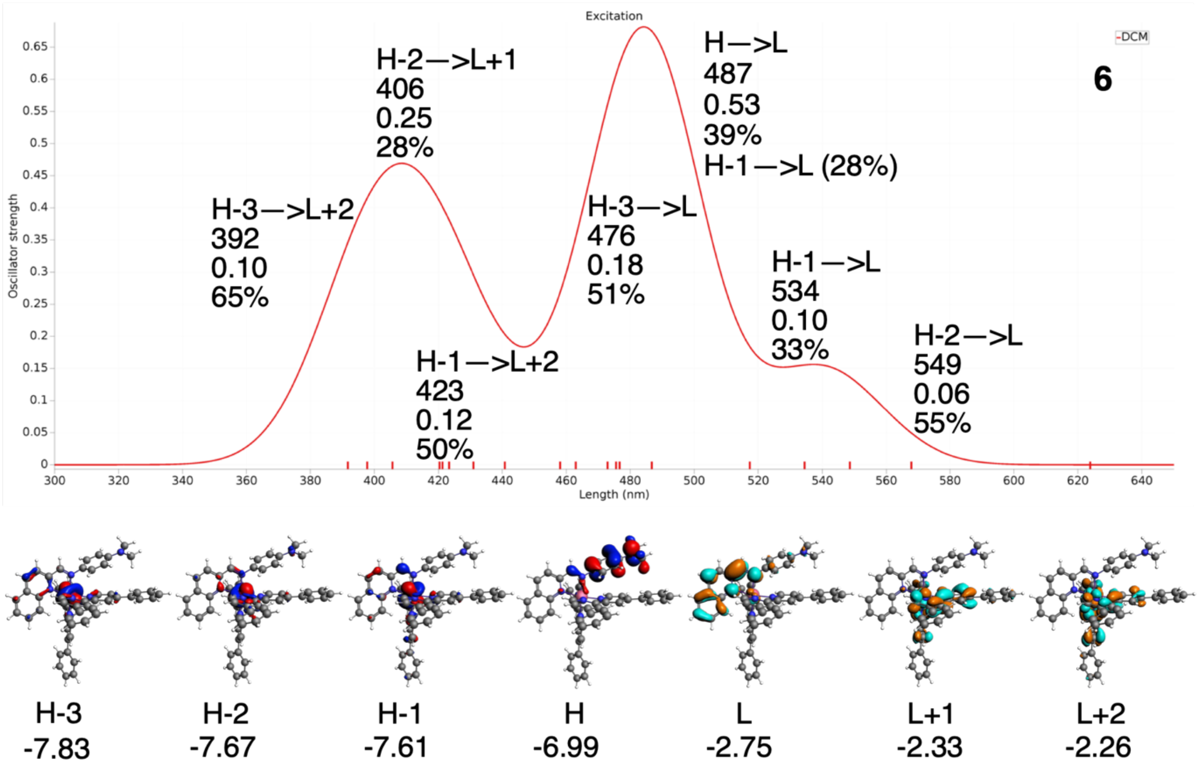

Figure S16. HOMO and LUMO orbitals of compound **2 – 6** together with their energies (in eV), computed at CAMY-B3LYP/TZ2P level in DCM (COSMO). HOMO-LUMO gaps amount to 5.40, 5.20, 4.48, 4.20 and 4.24 eV for **2**, **3**, **4**, **5**, and **6**, respectively, being HOMO destabilized (-7.58, -7.76, -6.96, -6.98, -6.99 eV from **2** to **6**) and LUMO stabilized (-2.19, -2.57, -2.49, -2.78, -2.75 eV from **2** to **6**) from **2** to **6**.

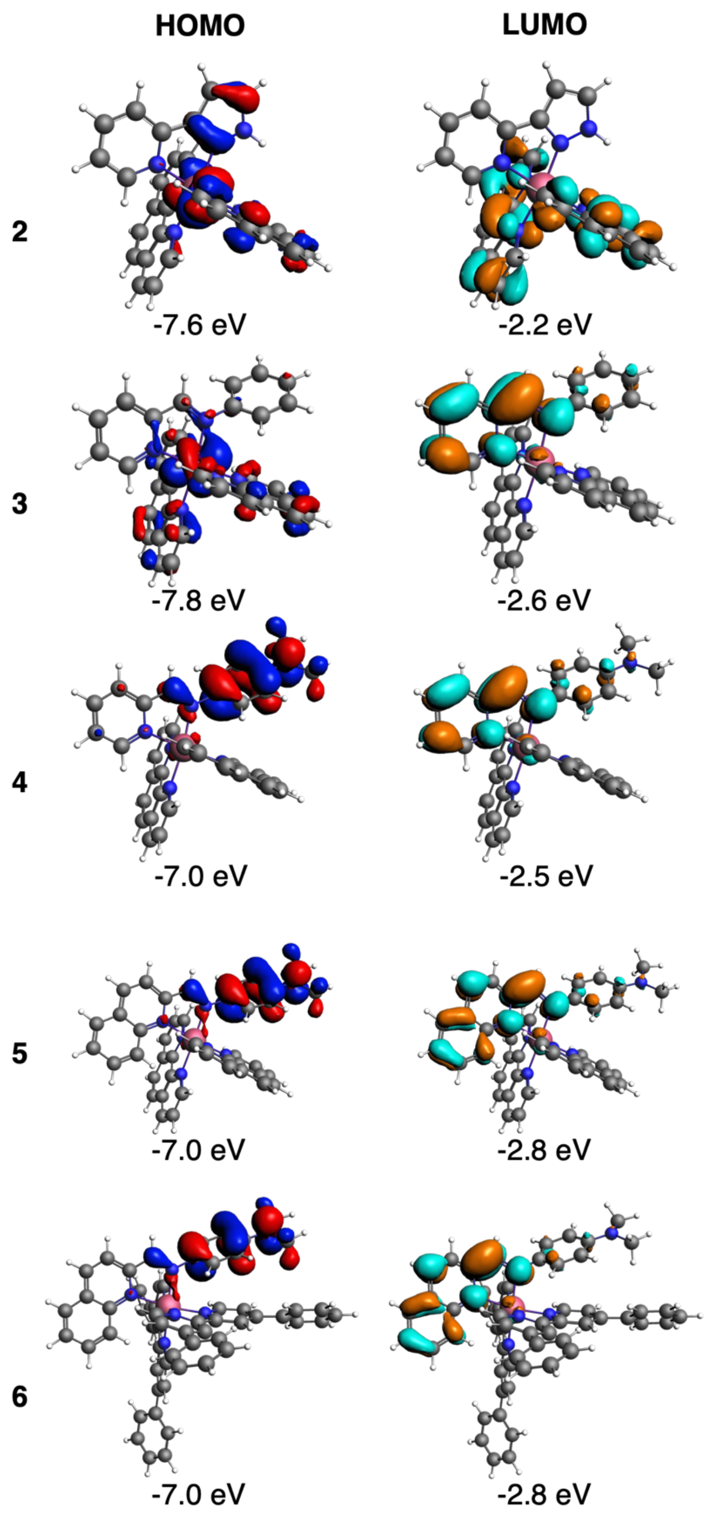

Figure S17. Photodegradation of Ru(II) complexes **2-6** in CH_2Cl_2 under increasing blue-light fluence (0–45 J cm^{-2}). Panels: (A) complex **2**, (B) complex **3**, (C) complex **4**, (D) complex **5**, and (E) complex **6**. (F) Evolution of normalized absorbance versus blue-light fluence for all complexes.

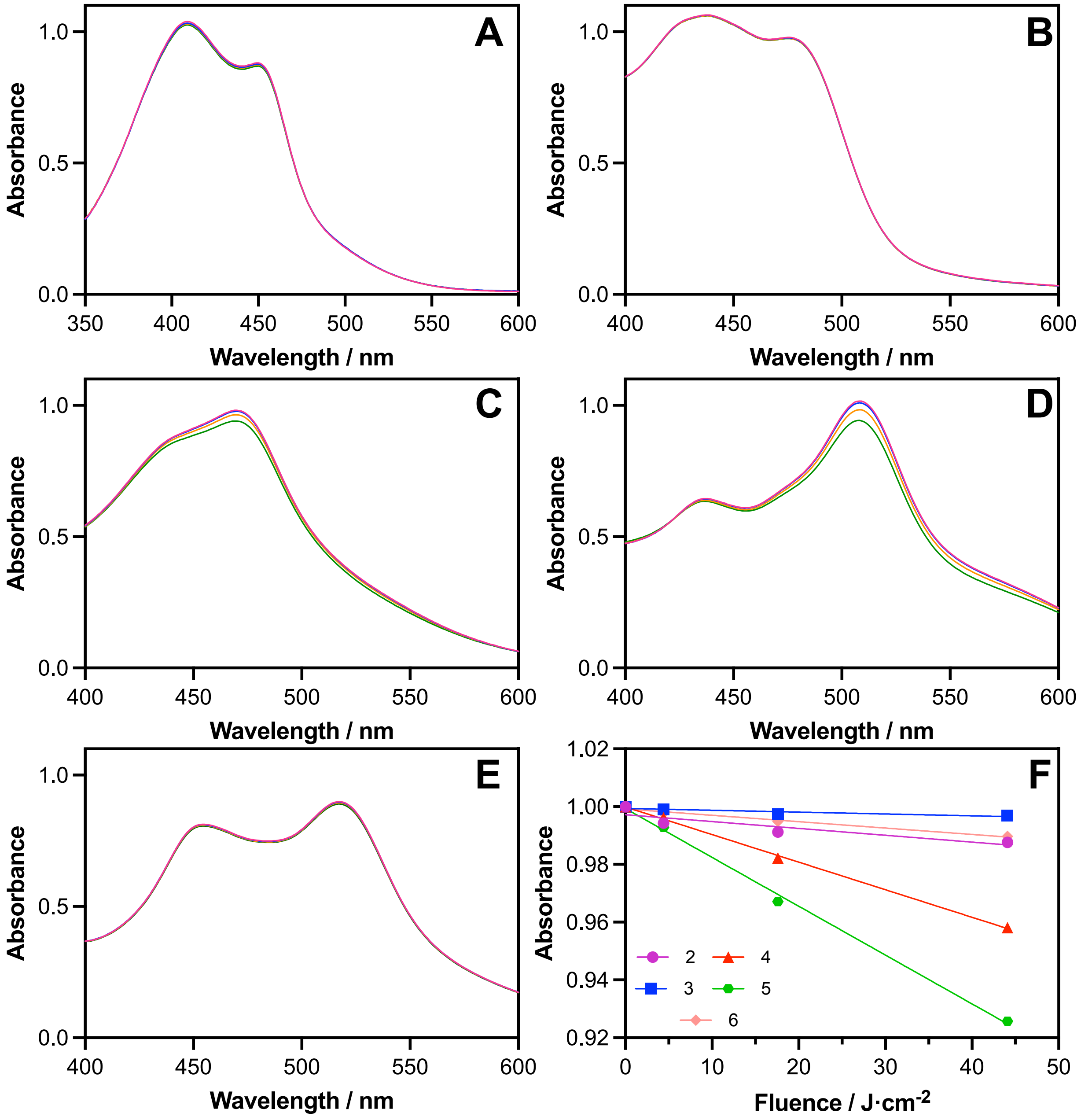

Figure S18. Photodegradation of Ru(II) complexes **2-6** in CH_3OH under increasing blue-light fluence (0–45 J cm^{-2}). Panels: (A) complex **2**, (B) complex **3**, (C) complex **4**, (D) complex **5**, and (E) complex **6**. (F) Evolution of normalized absorbance versus blue-light fluence for all complexes.

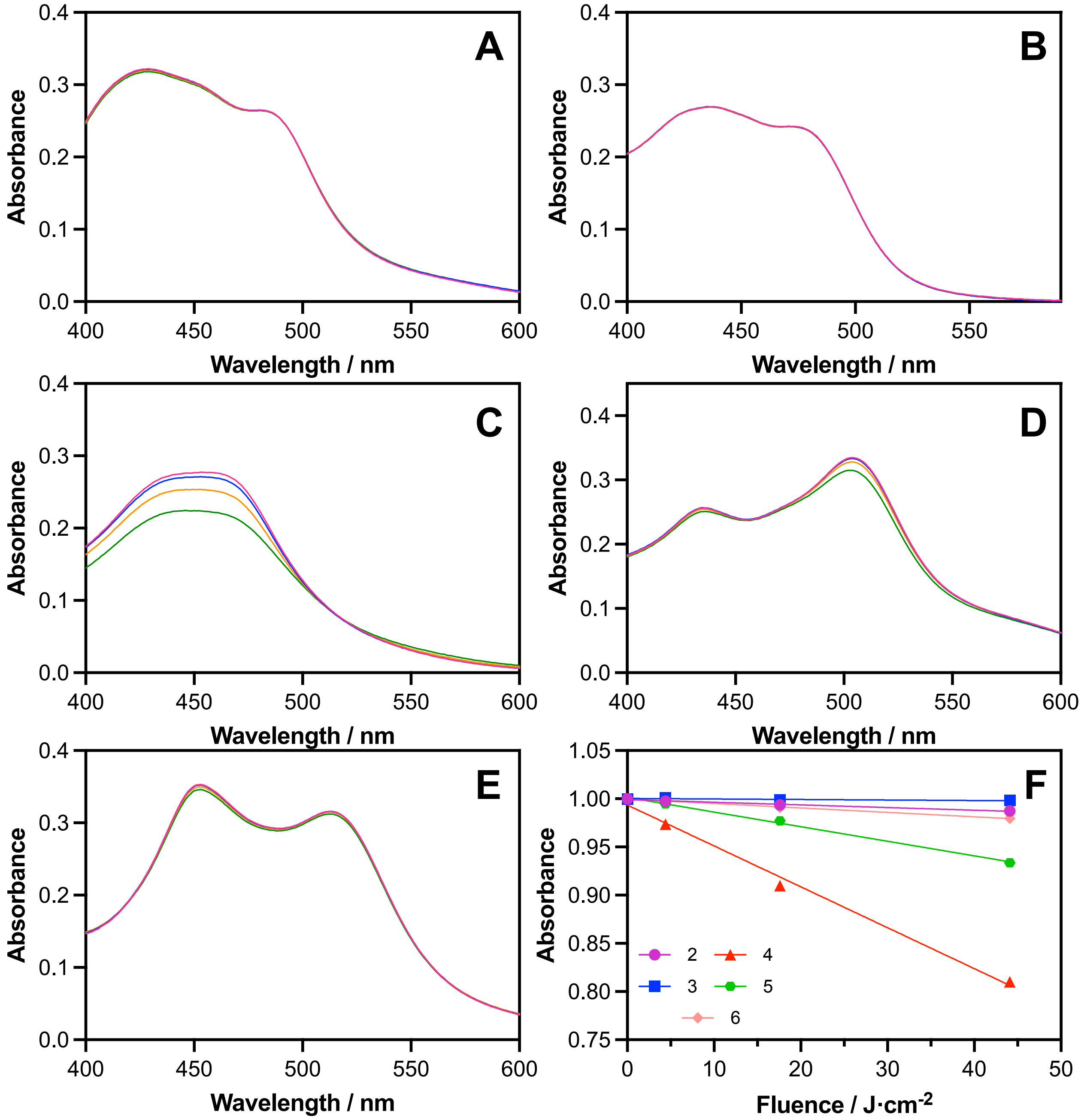

Figure S19. Emission spectra of complexes **2–6** in air-saturated dichloromethane (left) and methanol (right). All complexes were excited at 450 nm, where they have the same absorbance value. Quantum yield values were calculated from the areas under the spectra.

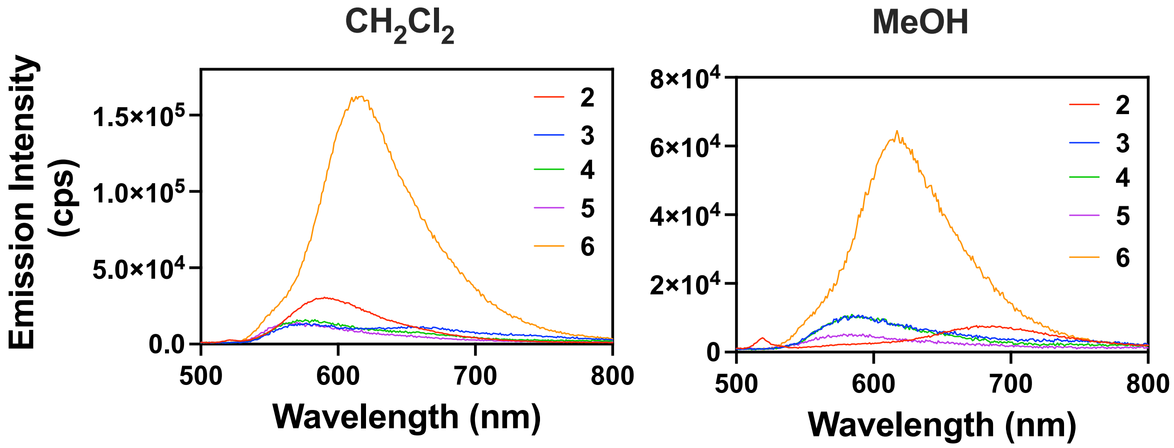

Figure S20. Involved molecular orbitals in the main emission bands (in nm) of the computed emission spectra of compounds **2 – 6** at TD-DFT BLYP-D3(BJ)/TZ2P level in DCM (COSMO).

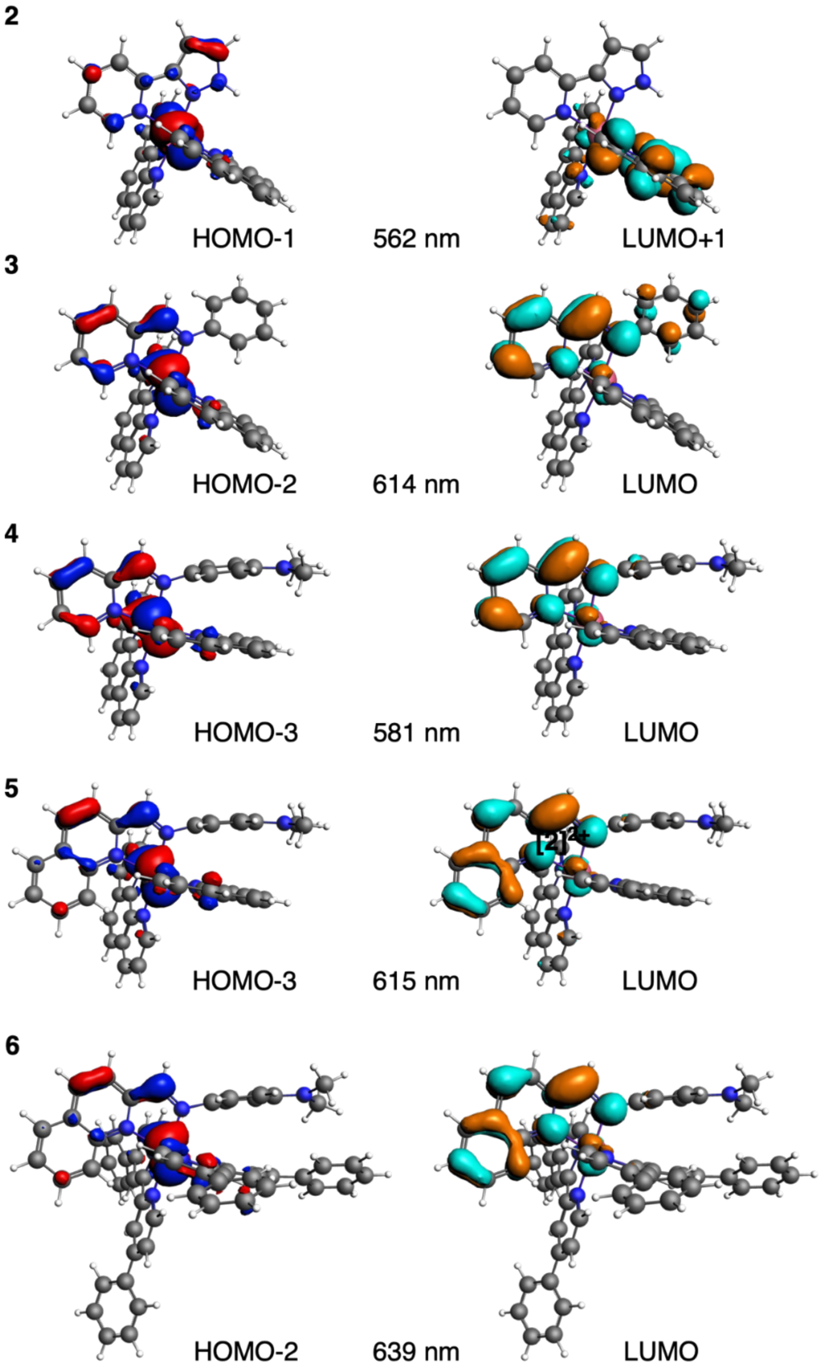

Complementary data for emission spectra (refer to Figure S20)
Singlet-singlet excitation energies. For each compound, first table encloses the energy, frequency, and electric dipole radiactive lifetime; whereas second table encloses for each excitation, the involved orbitals, its weight, and the contribution to the transition dipole moment (x,y,z).

Compound 2

no.	E/a.u.	E/eV	f	tau/s	Symmetry
1:	0.06136	1.66975	0.3517E-02	0.2350E-05	A
2:	0.06913	1.88111	0.1747E-02	0.3728E-05	A
3:	0.07221	1.96493	0.8769E-02	0.6807E-06	A
4:	0.07443	2.02525	0.6963E-02	0.8070E-06	A
5:	0.07488	2.03756	0.1270E-01	0.4371E-06	A
6:	0.07853	2.13681	0.4314E-01	0.1170E-06	A
**7:**	**0.08109**	**2.20664**	**0.1223**	**0.3869E-07**	**A**
8:	0.08276	2.25211	0.8232E-02	0.5519E-06	A
9:	0.08439	2.29632	0.5092E-01	0.8584E-07	A
10:	0.08578	2.33414	0.7669E-01	0.5516E-07	A

1: 153a	-> 154a	0.9942	-0.4310	-0.0530	-0.2344
2: 153a	-> 155a	0.9752	0.4585	-0.0169	-0.0172
3: 152a	-> 154a	0.8784	0.1045	0.4276	-0.5104
4: 153a	-> 156a	0.6285	-0.4391	0.1535	-0.2690
5: 153a	-> 157a	0.7232	-0.7459	0.1032	0.3172
6: 152a	-> 155a	0.4119	-0.5258	0.4190	-0.0355
**7: 152a(H-1)**	**-> 155a(L+1)**	**0.4718**	-0.5538	0.4413	-0.0374
8: 153a	-> 158a	0.8589	0.0452	-0.1220	0.3047
9: 152a	-> 156a	0.6823	-0.9980	-0.3517	0.0930
10: 152a	-> 157a	0.7330	-0.3313	0.2246	0.1080

HOMO: 153; LUMO: 154

Compound 3

no.	E/a.u.	E/eV	f	tau/s	Symmetry
1:	0.04998	1.36008	0.8525E-03	0.1461E-04	A
2:	0.06030	1.64088	0.1662E-01	0.5151E-06	A
3:	0.07113	1.93559	0.4153E-02	0.1481E-05	A
4:	0.07379	2.00783	0.6967E-02	0.8205E-06	A
**5:**	**0.07424**	**2.02020**	**0.1630**	**0.3465E-07**	**A**
6:	0.07766	2.11316	0.9560E-02	0.5399E-06	A
7:	0.07818	2.12741	0.2566E-02	0.1984E-05	A
8:	0.08270	2.25040	0.9418E-01	0.4832E-07	A
9:	0.08674	2.36040	0.1995E-01	0.2074E-06	A
10:	0.08835	2.40417	0.1879E-01	0.2122E-06	A

1: 163a	-> 164a	0.9925	-0.1908	0.0873	-0.0883
2: 162a	-> 164a	0.9775	0.3787	-0.8373	-0.3383
3: 163a	-> 165a	0.9844	-0.5351	-0.1824	-0.1238
4: 163a	-> 166a	0.9530	0.1382	0.0538	-0.1477
**5: 161a(H-2)**	**-> 164a(L)**	**0.8026**	0.2720	-1.6205	1.0764
6: 163a	-> 168a	0.6248	0.7320	-0.2135	-0.2009
7: 163a	-> 167a	0.5785	0.0716	0.1350	-0.3265
8: 162a	-> 165a	0.8318	-1.6289	0.5202	0.7105
9: 161a	-> 165a	0.5281	0.1795	0.1438	-0.3865
10: 162a	-> 167a	0.8164	-0.1382	0.4969	-0.9633

HOMO: 163; LUMO: 164

Compound 4

no.	E/a.u.	E/eV	f	tau/s	Symmetry
1:	0.02823	0.76825	0.1409E-02	0.2771E-04	A
2:	0.04784	1.30190	0.1593E-01	0.8534E-06	A
3:	0.05053	1.37493	0.1914E-02	0.6369E-05	A
4:	0.05386	1.46566	0.1929E-02	0.5563E-05	A
5:	0.05508	1.49880	0.5135E-02	0.1998E-05	A
6:	0.05567	1.51493	0.2671E-02	0.3759E-05	A
7:	0.06398	1.74101	0.1488E-01	0.5108E-06	A
8:	0.07419	2.01887	0.2320E-02	0.2437E-05	A
9:	0.07685	2.09129	0.1167E-01	0.4516E-06	A
**10:**	**0.07839**	**2.13323**	**0.1811**	**0.2797E-07**	**A**

1: 175a	-> 176a	0.9995	0.1165	0.3325	0.0852
2: 175a	-> 177a	0.9964	-0.3660	0.4749	0.6997
3: 175a	-> 178a	0.9925	-0.2852	0.1751	0.0178
4: 175a	-> 179a	0.9945	0.0912	0.1927	-0.2881
5: 175a	-> 180a	0.9596	-0.2272	-0.3432	-0.4005
6: 174a	-> 176a	0.9330	-0.1749	0.4982	-0.2884
7: 173a	-> 176a	0.9818	-0.4280	0.2795	0.3678
8: 174a	-> 177a	0.9735	-0.2320	-0.3266	-0.1867
9: 174a	-> 178a	0.9242	-0.0754	0.1523	0.0580
**10: 172a(H-3)**	**-> 176(L)**	**0.7189**	0.2241	-1.7931	0.9904

HOMO: 175; LUMO: 176

Compound 5

no.	E/a.u.	E/eV	f	tau/s	Symmetry
1:	0.02491	0.67787	0.7381E-03	0.6795E-04	A
2:	0.04861	1.32270	0.8109E-02	0.1624E-05	A
3:	0.05027	1.36794	0.2120E-02	0.5810E-05	A
4:	0.05214	1.41868	0.2991E-02	0.3829E-05	A
5:	0.05443	1.48113	0.1487E-02	0.7065E-05	A
6:	0.05480	1.49105	0.9677E-03	0.1071E-04	A
7:	0.06111	1.66289	0.1402E-01	0.5943E-06	A
**8:**	**0.07409**	**2.01613**	**0.2010**	**0.2820E-07**	**A**
9:	0.07444	2.02552	0.2003E-01	0.2805E-06	A
10:	0.07608	2.07030	0.4613E-02	0.1166E-05	A

1: 188a	-> 189a	0.9997	0.0969	0.2384	0.0670
2: 188a	-> 190a	0.9949	0.2718	-0.3440	-0.4844
3: 188a	-> 191a	0.9867	0.1961	-0.2175	-0.0140
4: 187a	-> 189a	0.9686	0.0825	-0.5479	0.0174
5: 188a	-> 192a	0.9966	0.0273	-0.0061	-0.2854
6: 188a	-> 193a	0.9959	-0.1892	-0.2564	-0.0865
7: 186a	-> 189a	0.9856	0.2459	0.3219	-0.6981
**8: 185a(H-3)**	**-> 189a(L)**	**0.8063**	-0.4003	2.0277	-0.8458
9: 187a	-> 190a	0.9389	0.3884	0.2333	0.1236
10: 187a	-> 191a	0.9707	-0.0124	0.2106	-0.0185

HOMO: 188; LUMO: 189

Compound 6

1:	0.02385	0.64906	0.2644E-03	0.2069E-03	A
2:	0.04406	1.19882	0.2027E-02	0.7912E-05	A
3:	0.04641	1.26278	0.6045E-03	0.2391E-04	A
4:	0.05015	1.36456	0.4001E-02	0.3093E-05	A
5:	0.05698	1.55058	0.2720E-01	0.3524E-06	A
6:	0.06537	1.77874	0.3796E-02	0.1919E-05	A
7:	0.06904	1.87873	0.4731E-02	0.1380E-05	A
8:	0.06922	1.88355	0.1174E-01	0.5534E-06	A
9:	0.07012	1.90803	0.8017E-03	0.7896E-05	A
**10:**	**0.07113**	**1.93546**	**0.2295**	**0.2680E-07**	**A**

1: 256a	-> 257a	0.9999	-0.1180	-0.1017	-0.0481
2: 256a	-> 258a	0.9973	-0.2175	0.1259	0.2098
3: 256a	-> 259a	0.9962	-0.1427	0.0143	-0.1071
4: 255a	-> 257a	0.9765	-0.2670	0.4604	-0.1769
5: 254a	-> 257a	0.9832	-0.4663	-0.1899	0.7601
6: 256a	-> 260a	0.9964	0.2117	0.2374	0.2764
7: 256a	-> 261a	0.9555	-0.0865	-0.1685	-0.1815
8: 255a	-> 258a	0.9416	0.4759	0.4572	0.3378
9: 256a	-> 262a	0.9967	0.0973	0.0033	-0.1887
**10: 253(H-3)**	**-> 257(L)**	**0.8436**	-0.6107	2.0055	-1.0249

HOMO: 256; LUMO: 257

Figure S21. Time-resolved emission kinetic traces of Ru(II) complexes **2–6** in dichloromethane (left) and methanol (right), measured under argon-saturated (red lines) and air-saturated (blue lines) conditions. λ_{exc} = 520 nm; λ_{obs} = 620 nm.

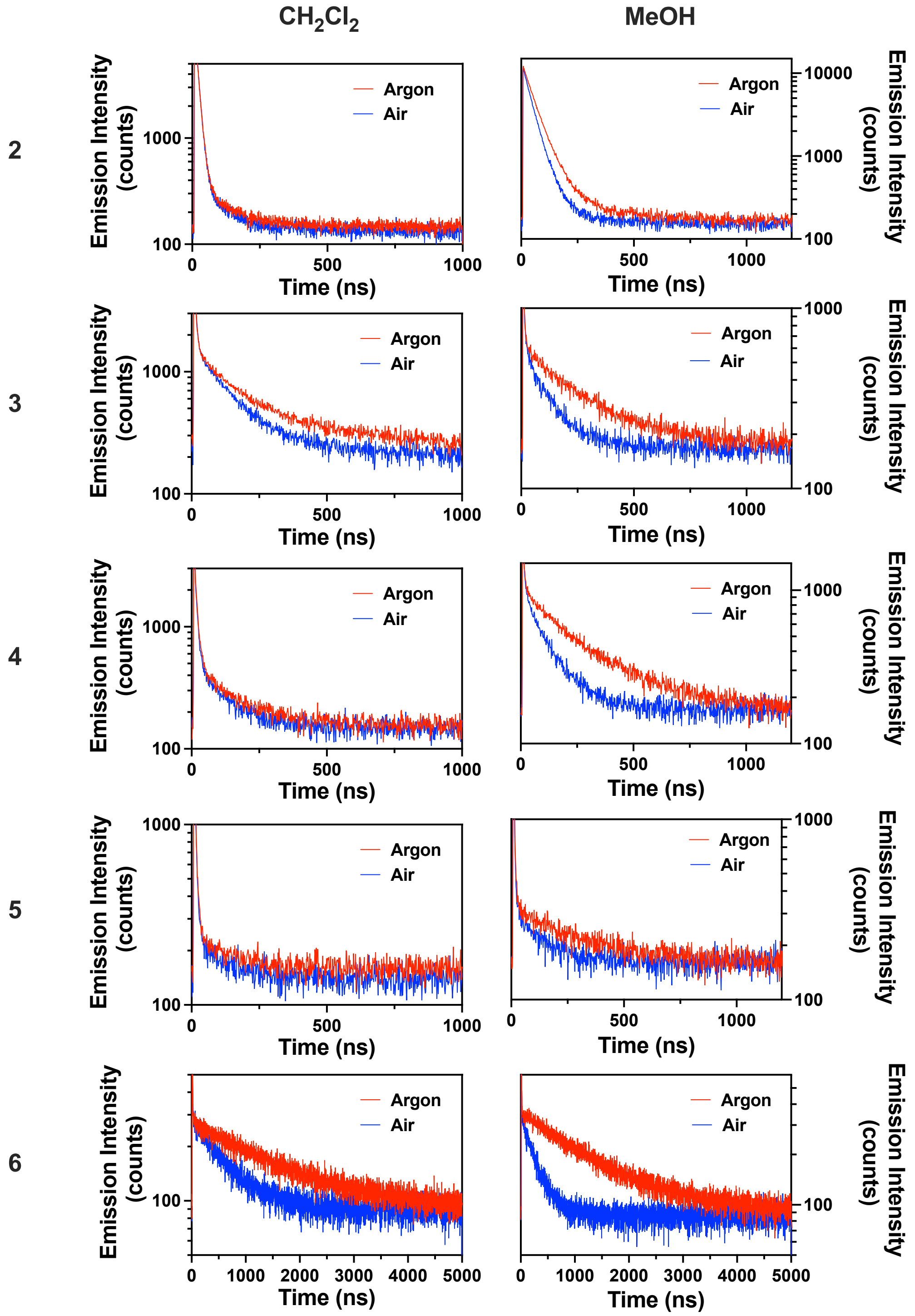

Figure S22. Determination of $^1O_2$ formation quantum yield (Φ_Δ). Time-resolved emission signals at 1275 nm, corresponding to $^1O_2$ phosphorescence, recorded in air-saturated dichloromethane (left) and methanol (right) for complexes **2–6** and phenalenone (used as the $^1O_2$ reference standard; $\Phi_\Delta = 1$). All solutions were adjusted to have identical absorbance at the excitation wavelength of 355 nm.

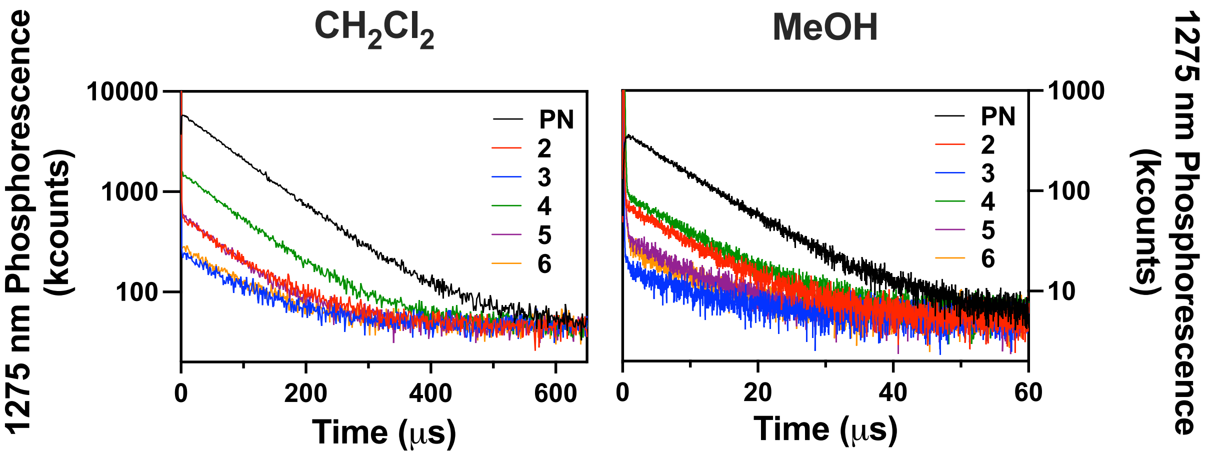

Figure S23. Detection of superoxide radical anion ($O_2^{\bullet-}$) sensitized by complexes **2-6** under blue-light irradiation. (A): Fluorescence increase of dihydroethidium (DHE) sensitized by complexes **2-6** and the reference tris(2,2′-bipyridine)ruthenium(II) ($[Ru(bpy)_3]^{2+}$) as a function of blue light fluence. Photoconversion of DHE in absence of any photosensitizer was carried out as negative control and oxidation of DHE by 0.5 mM KO_2 in darkness was carried out as positive control. All samples were adjusted to similar absorbance in the blue. (B): $O_2^{\bullet-}$ formation quantum yield ($\Phi_{O_2^{\bullet-}}$) relative to ($[Ru(bpy)_3]^{2+}$) $O_2^{\bullet-}$ photosensitization ability.

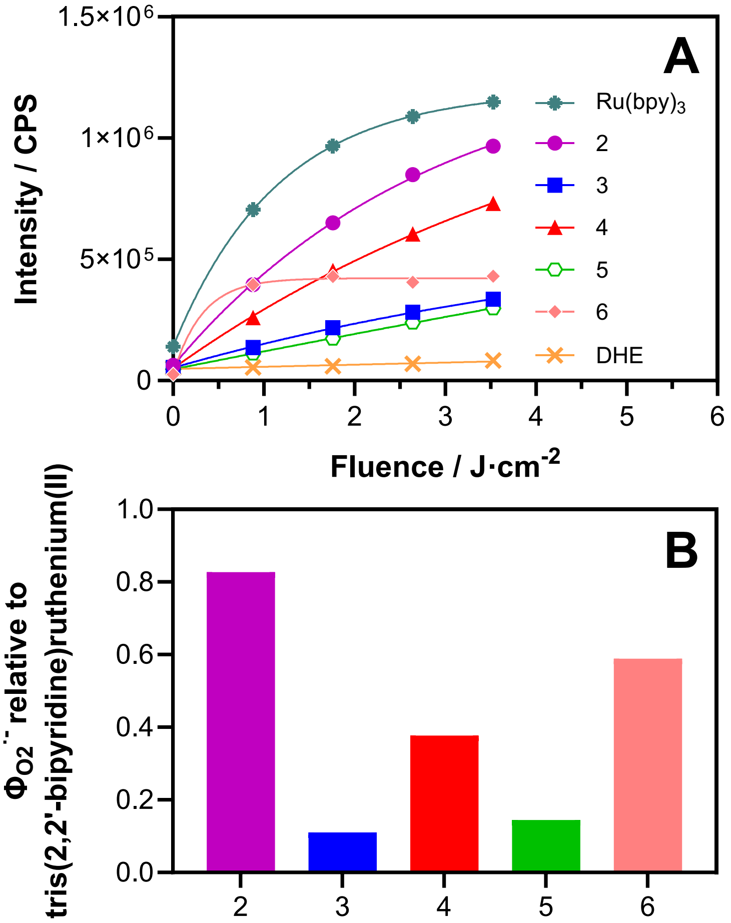

<u>**Note:**</u> the $\Phi_{O_2^{\bullet-}}$ values were determined from the ratio of slopes of DHE fluorescence increase for each Ru complex relate to the ($[Ru(bpy)_3]^{2+}$) reference photosensitizer, evaluated at low conversion (< 2 J/cm^2; Eq. S1). Because a non-monochromatic light source was used, a photon-absorption correction factor (CF) was applied to compensate for small differences in the number of photons absorbed by sample and reference (Eq. S2).

$$\phi_{O_2{}^{\cdot-};Sample} = \frac{Slope_{Sample}}{Slope_{Ref}} \cdot CF \qquad Eq.S1$$

$$CF = \frac{\#Absorbed\ photons\ Ref}{\#Absorbed\ photons\ sample} = \frac{\int_0^\infty I_{exc;\lambda} \cdot \left(1 - 10^{-A_{Ref;\lambda}}\right) \cdot d\lambda}{\int_0^\infty I_{exc;\lambda} \cdot \left(1 - 10^{-A_{Sample;\lambda}}\right) \cdot d\lambda} \qquad Eq.S2$$

Where $I_{exc;\lambda}$ is the fraction of photons emitted by the irradiation source at each excitation wavelength; and $(1 - 10^{-A_{Ref;\lambda}})$ is the fraction of photons absorbed by the sample or the reference for each excitation wavelength.

Figure S24. Jablonski diagrams for different excited states in the set of ruthenium complexes.

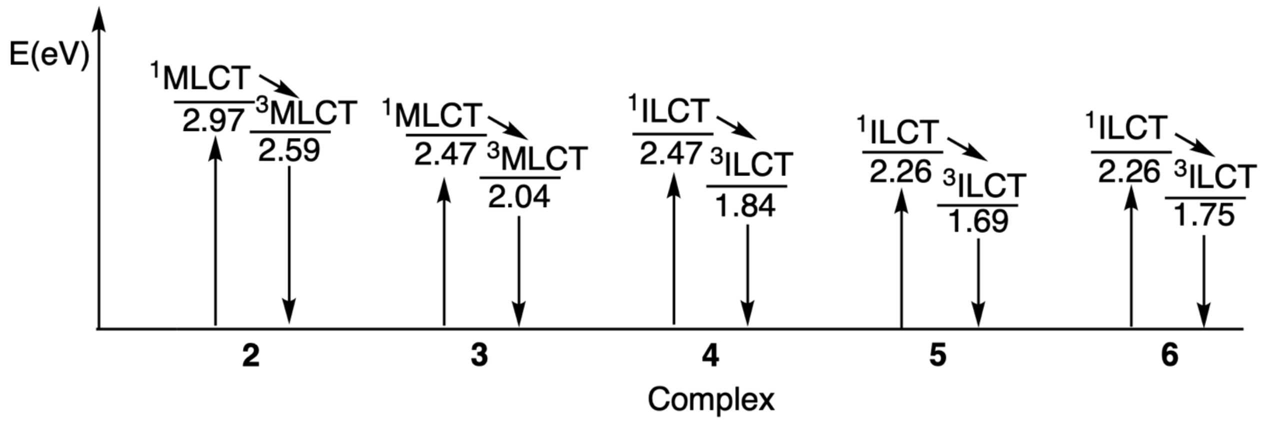

Figure S25. Normalized absorption spectra of the Ru(II) complexes (**2**-**6**) in the absence and presence of 50 µg/mL calf thymus DNA (ctDNA).

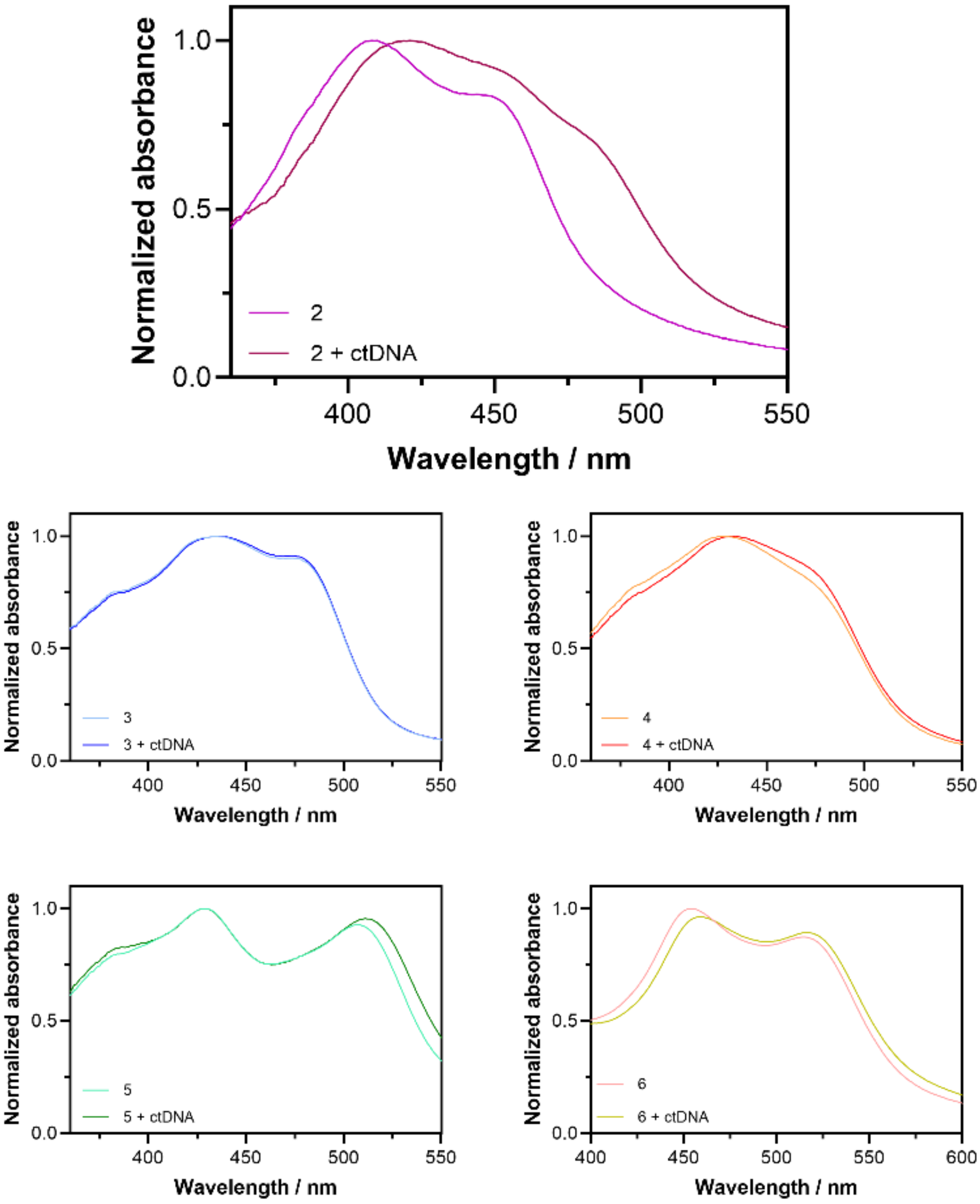

Figure S26. Photodegradation of the Ru(II) complexes (**2**-**6**) in the absence and presence of 50 μg/mL ctDNA upon irradiation with blue light (40 J/cm^2).

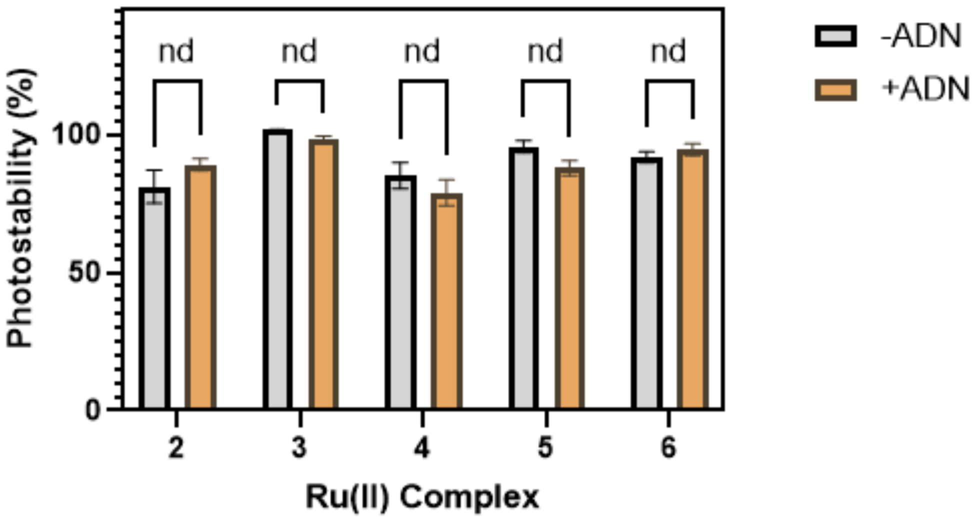

Supplement: Supplementary file 1 [file ic6c00420_si_001.pdf]
